# Synthesis, characterization and biological research of novel 2-(quinoline-4-carbonyl)hydrazide-acrylamide hybrids as potential anticancer agents on MCF-7 breast carcinoma cells by targeting EGFR-TK†

**DOI:** 10.1039/d4ra03963g

**Published:** 2024-07-26

**Authors:** Hany M. Abd El-Lateef, Duaa Bafail, Noura Hamdi Yousef Alhalees, Eslam E. M. Toson, Ali H. Abu Almaaty, Elsherbiny H. Elsayed, Islam Zaki, Magdy M. Youssef

**Affiliations:** a Department of Chemistry, College of Science, King Faisal University Al-Ahsa 31982 Saudi Arabia hmahmed@kfu.edu.sa; b Department of Chemistry, Faculty of Science, Sohag University Sohag 82524 Egypt; c Department of Clinical Pharmacology, Faculty of Medicine, King Abdulaziz University Jeddah Saudi Arabia; d Ministry of Health, King Abdullah Medical Complex Jeddah Saudi Arabia; e Chemistry Department, Faculty of Science, Port Said University Port Said 42526 Egypt; f Zoology Department, Faculty of Science, Port Said University Port Said 42526 Egypt; g Pharmaceutical Organic Chemistry Department, Faculty of Pharmacy, Port Said University Port Said Egypt Eslam.Zaki@pharm.psu.edu.eg; h Biochemistry Division, Chemistry Department, Faculty of Science, Mansoura University Mansoura Egypt

## Abstract

Novel derivatives of the 2-(quinoline-4-carbonyl)hydrazide scaffold carrying the acrylamide moiety were synthesized and tested for their cytotoxic efficacy against the breast carcinoma MCF-7 cell line. The most active members 6a, 6b and 6h revealed significant antiproliferative action with an IC_50_ value of 3.39, 5.94 and 2.71 μM, respectively, which were more potent than the reference drug Dox (IC_50_ = 6.18 μM). Aiming to enlighten the antiproliferative activity, compounds 6a and 6h were examined for their inhibitory potential against EGFR kinase. The results demonstrated that compound 6h displayed potent inhibitory activity, as concluded from the IC_50_ value (IC_50_ = 0.22 μM) compared to the standard drug Lapatinib (IC_50_ value of 0.18 μM). Compound 6h was found to induce significant cellular cycle arrest at the G1 phase and provoke apoptosis. Besides, compound 6h triggered apoptosis *via* upregulating p53 and initiator caspase 9 by 7.4- and 8.7-fold, respectively, compared to DMSO controls.

## Abstract 2

A sequence of novel 2-(quinoline-4-carbonyl)hydrazide scaffolds carrying the acrylamide moiety have been synthesized and evaluated for *in vitro* cytotoxicity against an MCF-7 breast cancer cell line.

## Graphical abstract

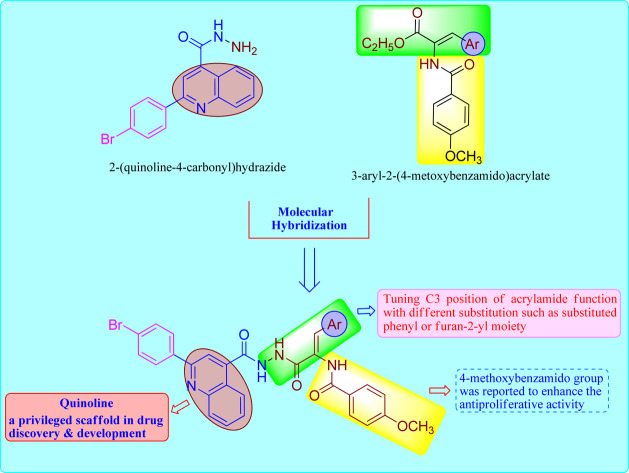

## 1. Introduction

Cancer continues to be the most difficult illness to cure and the second-largest cause of death in the world.^1^ Despite current therapeutic protocols, the majority of patients acquire drug resistance, develop tumor recurrence metastasis and have low survival rates.^2,3^ Therefore, the establishment of novel therapies that are both less harmful and more effective is one of the most intensely pursued goals of contemporary medicinal chemistry.^4,5^ Understanding the molecular mechanisms that regulate growth and survival of tumor cells would provide insights into potential targets for drug design and synthesis.^6,7^ Epidermal growth factor receptor (EGFR) is known to be the key regulator of many complex biological processes, including cell motility, cell cycle regulation as well as apoptosis activation and metastasis.^8,9^ The aberrant expression of EGFR has been correlated with cancer progression.^10^ Recently, reports have shown evidence supporting the overexpression of EGFR in many cancer patients, which indicates that inhibition of EGFR could be beneficial in cancer treatment.^11,12^ In addition, many tumors exhibited increased expression of anti-apoptotic factors and decreased expression of apoptotic mediators.^13^ For example, caspase 9; an initiator of tumor-mediated apoptosis is expressed at low levels in many tumor cell lines.^14^ The modulation of anti-apoptotic and pro-apoptotic gene expression contributes to increased proliferation and survival resulting in tumor growth, suggesting potential as druggable treatment targets.^15^

The biological importance of quinoline derivatives has drawn a lot of attention to their chemistry.^16–18^ Quinoline based derivatives displayed cancer fighting properties in many tumor types through EGFR signalling inhibition.^19^ In addition, it is well documented that quinoline compounds shown to deplete the proliferation and prompted caspase-dependent death of MCF-7 cell *in vitro*.^20^ It also enhances the expression of p53 before apoptosis.^21^ These findings point to the quinoline moiety as promising agent for the establishment for the creation of cancer therapy.

In recent years, synthesis of acrylamide derivatives has been regarded of great interest to organic chemistry owing to their therapeutic properties.^22^ Acrylamide scaffold exhibited various biological properties including antitumor *via* EGFR tyrosine kinase inhibition.^23^ Furthermore, it is also evidenced from literature that quinoline moiety linked to acrylamide function with different substitution pattern possess a wide range of anticancer properties.^24^ Many acrylamide containing derivatives were evaluated for their cytotoxic capacity *via* inhibition of EGFR-TK signalling. In addition, acrylamide-based anticancer agents showed great potential for the treatment of cancer by induction of cell cycle arrest, differentiation, and cellular apoptosis.^25,26^ Afatinib, an acrylamide-containing drug, is an EGFR kinase inhibitor indicated for the treatment of adult patients with metastatic, non-small lung cancer.^27^ It covalently binds to the kinase domain of EGFR and irreversibly inhibits tyrosine kinase autophosphorylation, resulting in downregulation of EGFR downstream signaling.^28^

**Fig. 1:**
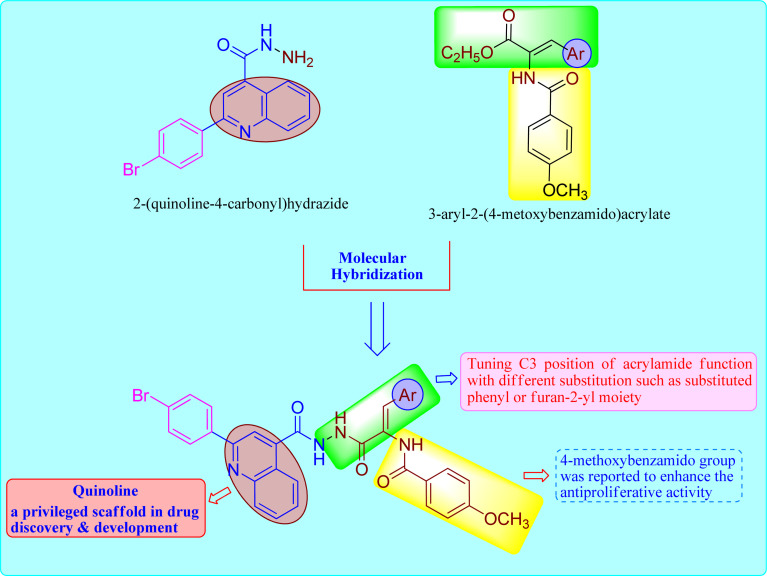
The design concept used for the synthesis of the title compounds 5 and 6a–i.

Based on these observations, we designed the synthesis of new set of quinoline-acrylamide hybrids 5 and 6a–i to be checked for their cytotoxic potential and EGFR kinase inhibition. The target derivatives will incorporate both quinoline-4-carbohydrazide structure as apoptosis inducer^29^ connected to 3-arylacrylamide moiety, which was reported to efficiently inhibit kinase receptor^30,31^ (Fig. 1). Furthermore, cell cycle and apoptosis activation will be determined for the most active hybrid to give idea about the phase at which these new hybrids halt the growth of cancer cells. Lastly, to further confirm apoptosis induction by the most active member, the treated cancer cells were subjected to qRT-PCR measurements to quantify the level of apoptotic markers such p53 and effector caspase 9 compared to DMSO controls.

## 2. Results and discussion

### 2.1. Chemistry

**Scheme 1:**
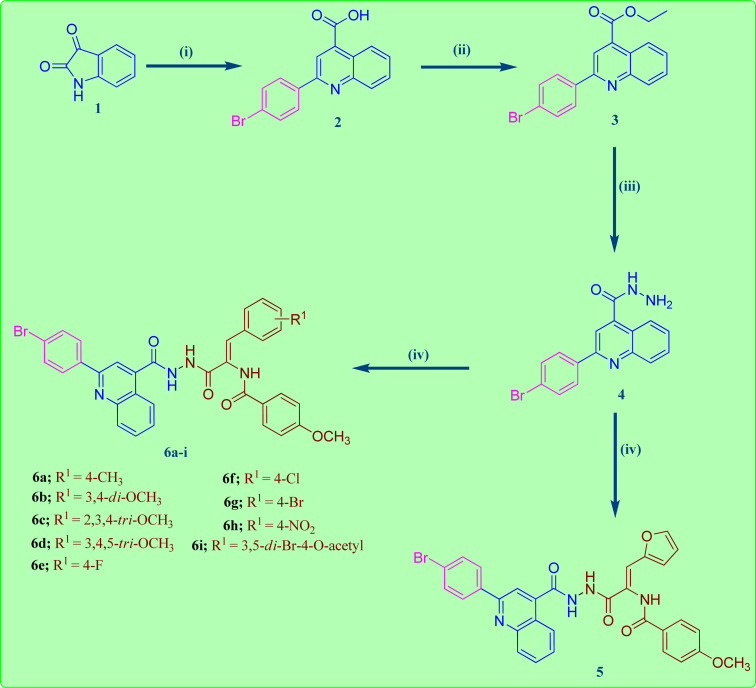
Synthesis of the target 2-(quinoline-4-carbonyl)hydrazide-acrylamide hybrids 5 and 6a–i. Reagents: (i) 4-Br-C_6_H_4_COCH_3_, 33% KOH, C_2_H_5_OH, reflux 12 h; (ii) C_2_H_5_OH, Conc. H_2_SO_4_, reflux 12 h; (iii) NH_2_NH_2_, C_2_H_5_OH, reflux 7 h; (iv) respective 2-(4-methoxybenzamido)carboxylate, NaOAc, C_2_H_5_OH, reflux 18 h.

The reaction sequences employed for the preparation of the target quinoline-4-carbohydrazide–acrylamide hybrids are illustrated in Scheme 1. Commercially available Istatin molecule 1 was utilized for the synthesis of quinoline-4-carboxylic acid 2*via* its reaction with 4-bromoacetophenone and 33% potassium hydroxide following a previously reported procedure by heating the reactants at reflux in the existence of ethanol.^32^ The key ethyl quinoline-4-carboxylate was prepared by reaction of quinoline-4-carboxylic acid 2 and ethanol in the existence of catalytic amount of Conc. H_2_SO_4_.^33^ In the present study, the quinoline-4-carbohydrazinyl intermediate 4 was prepared through condensation of ethyl quinoline-4-carboxylate with hydrazine *via* heating the reactants at reflux in the existence of ethanol.^34^ The target 2-(quinoline-4-carbonyl)hydrazide-acrylamide hybrids 5 and 6a–i were synthesized through the reaction of quinoline-4-carbohydrazinyl intermediate 4 with the respective ethyl(*Z*)-3-aryl-2-(4-methoxybenzamido)acrylate derivative in the existence of ethanol and catalytic quantities of anhydrous sodium acetate. The structures of novel 2-(quinoline-4-carbonyl)hydrazide-acrylamide hybrids 5 and 6a–i were authenticated by using spectral techniques (^1^H-NMR and ^13^C-NMR). For instance, the ^1^H-NMR spectrum of 2-(quinoline-4-carbonyl)hydrazide-3-(4-chlorophenyl)acrylamide hybrid 6f as example, confirmed the presence of three amide NH groups through the existence of three broad singlet peaks at *δ*_H_ 10.81, 10.54 and 9.98 ppm. In addition to the presence of singlet peak at *δ*_H_ 7.36 ppm attributed to C2–H of acrylamide group. Moreover, the ^1^H-NMR spectrum of 2-(quinoline-4-carbonyl)hydrazide-3-(4-chlorophenyl)acrylamide 6f showed a characteristic doublet signal at *δ*_H_ 8.44 ppm for the C5–H proton. Additionally, the presence of two triplet peaks at *δ*_H_ 7.88 and 7.71 ppm with integration of one proton each, disclosing to C7–H and C6–H of quinoline moiety, respectively. Also, the ^1^H-NMR spectrum of 2-(quinoline-4-carbonyl)hydrazide-3-(4-chlorophenyl)acrylamide 6f indicated a singlet signals of three protons at *δ*_H_ 3.86 ppm pointing to the existence of methoxy group of the 4-methoxybenzamide moiety. Also, the structure of 2-(quinoline-4-carbonyl)hydrazide-3-(4-chlorophenyl)acrylamide 6f was authenticated by ^13^C-NMR technique which showed the presence of new shielded peak at *δ*_c_ 55.93 ppm attributed to the methoxy carbon (OCH_3_). In addition, the aromatic and acrylamide carbon atoms showed up in the aromatic region at 114.02–148.30 ppm. In addition, the characteristic C2 quinoline carbon was displayed in the ^13^C-NMR spectrum at *δ*_c_ 155.01 ppm. Moreover, the three amide carbonyls of quinolinecarbohydrazide, acrylamide and 4-methoxybenzamide moieties were appeared in the ^13^C-NMR spectrum of 2-(quinoline-4-carbonyl)hydrazide-3-(4-chlorophenyl)acrylamide 6f at *δ*_c_ 165.10, 165.98 and 166.13 ppm, respectively.

### 2.2. Biological evaluation

#### 2.2.1. Cytotoxic activity against MCF-7 cells

In this study, all targeted 2-(quinoline-4-carbonyl)hydrazide-acrylamide hybrids 5 and 6a–i have been designed and their cytotoxic potential was examined *in vitro* against MCF-7 breast carcinoma cell line. The MTT colorimetric technique was used to evaluate the cytotoxic nature of these hybrids, with the widely utilized chemotherapeutic agent Doxorubicin (Dox) as reference control. The obtained IC_50_ results were compared with Dox, as presented in Table 1. The collected results demonstrated that all the tested 2-(quinoline-4-carbonyl)hydrazide-acrylamide hybrids 5 and 6a–i hybrids except 6c showed modest to significant cytotoxic impact on the tested breast carcinoma with IC_50_ that are ranged from 2.71–39.13 μM. In general, four hybrids 6a, 6b, 6h and 6i displayed significant cytotoxic effect against the examined cell line with IC_50_ that are ranged from 2.71–8.77 μM; in particular 2-(quinoline-4-carbonyl)hydrazide-3-(4-nitrophenyl)acrylamide 6h was the most potent hybrid with IC_50_ value of 2.71 μM compared to DOX (IC_50_ = 6.18 μM). Considering acrylamide moiety bearing phenyl group with halogen substituent at C3 position of acrylamide, a substantial drop in the cytotoxic activity was showed in compounds 6e, 6f and 6g which contain fluorine, chlorine and bromine group, which demonstrated modest IC_50_ value of 21.18, 26.01 and 39.13 μM, respectively against the tested cell line compared to DOX (IC_50_ = 6.18 μM). In addition, the replacement of the 4-Br substituent with 3,5-di-Br-4-acetoxyphenyl moiety 6i (IC_50_ = 8.77 μM) resulted in an increase of the biological activity. Furthermore, the activity intensified to became potent with the aryl group carrying electron donating substituent at C3 position of acrylamide moiety in 2-(quinoline-4-carbonyl)hydrazide-3-(4 methylphenyl)acrylamide 6a (IC_50_ = 3.39 μM) and 2-(quinoline-4-carbonyl)hydrazide-3-(3,4- dimethoxyphenyl)acrylamide 6b (IC_50_ = 5.94 μM) having 4-methyl and 3,4-dimethoxyphenyl group, respectively. On the other hand, compound 6d (IC_50_ = 17.10 μM) with 3,4,5-trimethoxyphenyl moiety resulted in a decrease in activity with one third the cytotoxic activity as Dox (IC_50_ = 6.18 μM). Alternatively, 2-(quinoline-4-carbonyl)hydrazide-acrylamide hybrid 5 (IC_50_ = 12.47 μM) bearing furan ring on C3 of acrylamide moiety elicited half the cytotoxic activity as Dox (IC_50_ = 6.18 μM).

**Table 1:** Antiproliferative activity of synthesized 2-(quinoline-4-carbonyl)hydrazide-acrylamide hybrids 5 and 6a–i*versus* MCF-7 cell line. Results indicate mean ± SD, *n* = 3

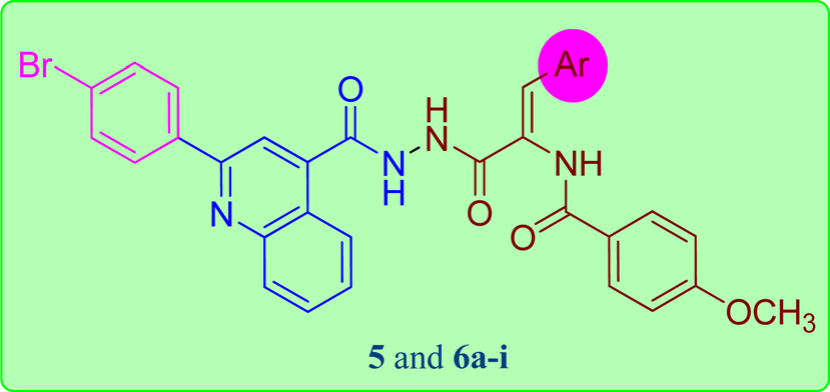		
Comp. no.	Ar	IC_50_ (μM)
		MCF-7
5	Furan-2-yl	12.47 ± 0.40
6a	4-CH_3_–C_6_H_4_–	3.39 ± 0.11
6b	3,4-Di–OCH_3_–C_6_H_3_–	5.94 ± 0.17
6c	2,3,4-Tri–OCH_3_–C_6_H_2_–	51.77 ± 1.54
6d	3,4,5-Tri–OCH_3_–C_6_H_2_–	17.10 ± 0.55
6e	4-F–C_6_H_4_––	21.18 ± 0.68
6f	4-Cl–C_6_H_4_––	26.01 ± 0.84
6g	4-Br–C_6_H_4_––	39.13 ± 1.26
6h	4-NO_2_–C_6_H_4_–	2.71 ± 0.09
6i	3,5-Di-Br-4-OAcetyl–C_6_H_2_––	8.77 ± 0.28
Dox	—	6.18 ± 0.20

#### 2.2.2. EGFR-TK inhibition assay

**Fig. 2:**
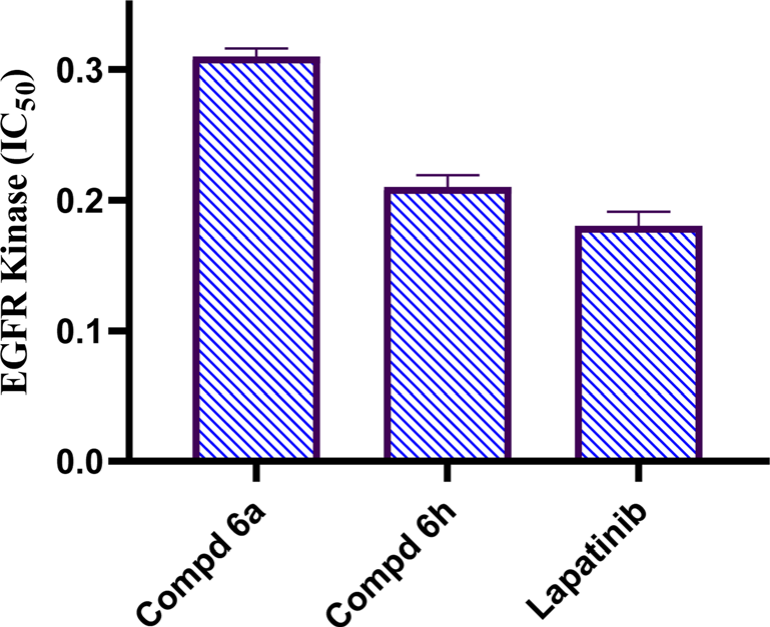
Results of EGFR-TK inhibition test as IC_50_ result (μM) of tested 2-(quinoline-4-carbonyl)hydrazide-acrylamide derivatives 6a, 6h and lapatinib.

The EGFR regulates information for signal transduction in a variety of biological processes, including cell proliferation, during division, and apoptosis activation.^35^ Inhibitors of small molecules are useful for targeting EGFR-TK signalling in the initiation and advancement of cancer disease.^36^ Therefore, EGFR-TK inhibitors have emerged as a promising area for drug creation and research study.^37^ To provide mechanistic understanding of the anticancer potential of the most active hybrids, 2-(quinoline-4-carbonyl)hydrazide-acrylamide hybrids; 6a and 6h revealing the highest cytotoxic activity against breast carcinoma cell line, they were examined for their EGFR-TK inhibition activities. Results obtained as an IC_50_ (μM) are presented in Fig. 2. 2-(Quinoline-4-carbonyl)hydrazide-3-(4-nitrophenyl)acrylamide 6h was the strongest inhibitor against EGFR with an IC_50_ value of 0.22 μM which is nearly equipotent to standard drug Lapatinib (IC_50_ value of 0.18 μM). The other compound; 2-(quinoline-4-carbonyl)hydrazide-3-(4-methylphenyl)acrylamide 6a possessed mild inhibitory activity against EGFR with IC_50_ value of 0.31 μM. Depending on the outputs of cytotoxic activity and EGFR kinase inhibition, 2-(quinoline-4-carbonyl)hydrazide-3-(4-nitrophenyl)acrylamide 6h was selected for further mechanistic investigations.

#### 2.2.3. Cell cycle analysis

**Fig. 3:**
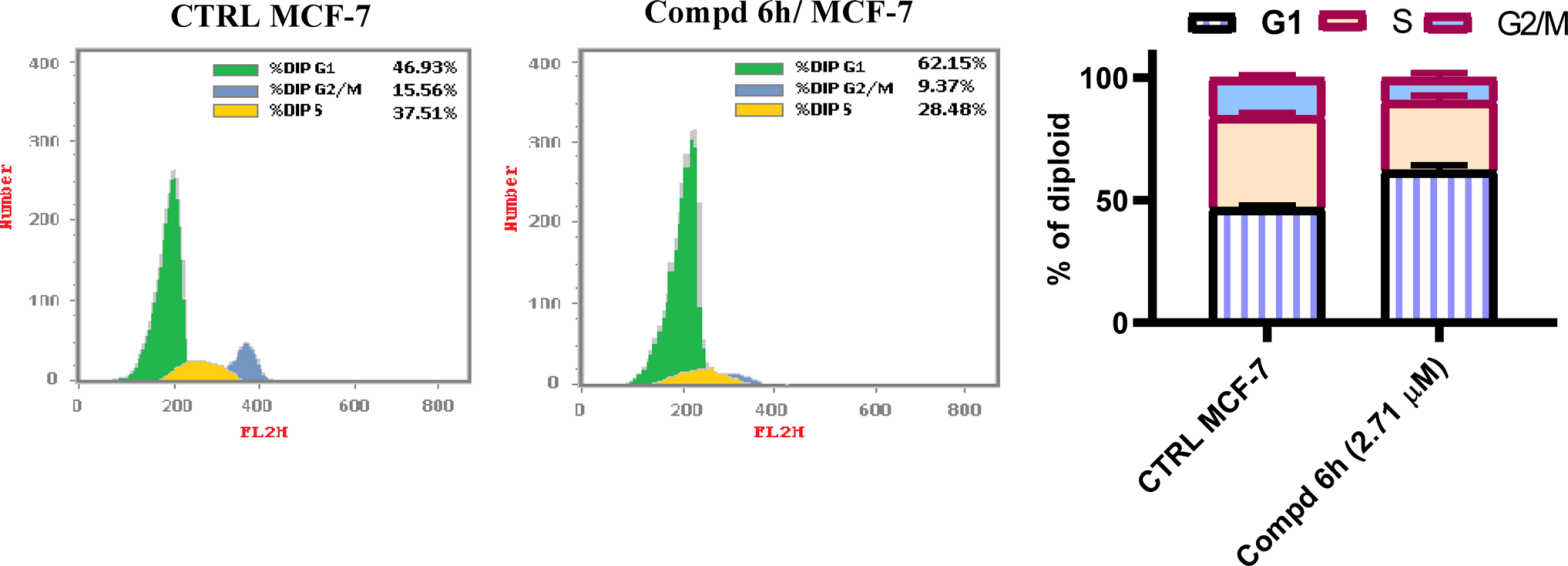
2-(Quinoline-4-carbonyl)hydrazide-3-(4-nitrophenyl)acrylamide 6h alters cellular cycle distribution in MCF-7 cells treated with 2.71 μM of compound 6h and compared to DMSO controls.

It is well documented that most tyrosine kinase inhibitors exert strong antiproliferative and pro-apoptotic effect.^38^ In this regard, to evaluate the molecular mechanism of action, the cellular and biochemical effects of 2-(quinoline-4-carbonyl)hydrazide-acrylamide hybrid with highest cytotoxicity were studied; *i.e.*6h bear 4-nitrophenyl moiety at the position 3 of acrylamide moiety. Flow cytometric assessments of the life cycle in MCF-7 cells that have been treated with 2-(quinoline-4-carbonyl)hydrazide-3-(4-nitrophenyl)acrylamide 6h at the IC_50_ concentration (IC_50_ = 2.71 μM) revealed a significant alterations in cellular cycle distribution, with considerable accumulation of cells in G1 phase and a decrease of both S and G2/M phases populations. It is notable that, the proportion of G1 phase was increased from 46.93% to 62.15% compared to DMOS untreated control group (Fig. 3). On the other hand, the percentage of S and G2/M phases was decreased from 37.51 and 15.56% to 28.48 and 9.37%, respectively for DMSO untreated control group. These findings are in agreement with the previously observed for EGFR-TK structural analogues.^39^

#### 2.2.4. Apoptosis staining assay

**Fig. 4:**
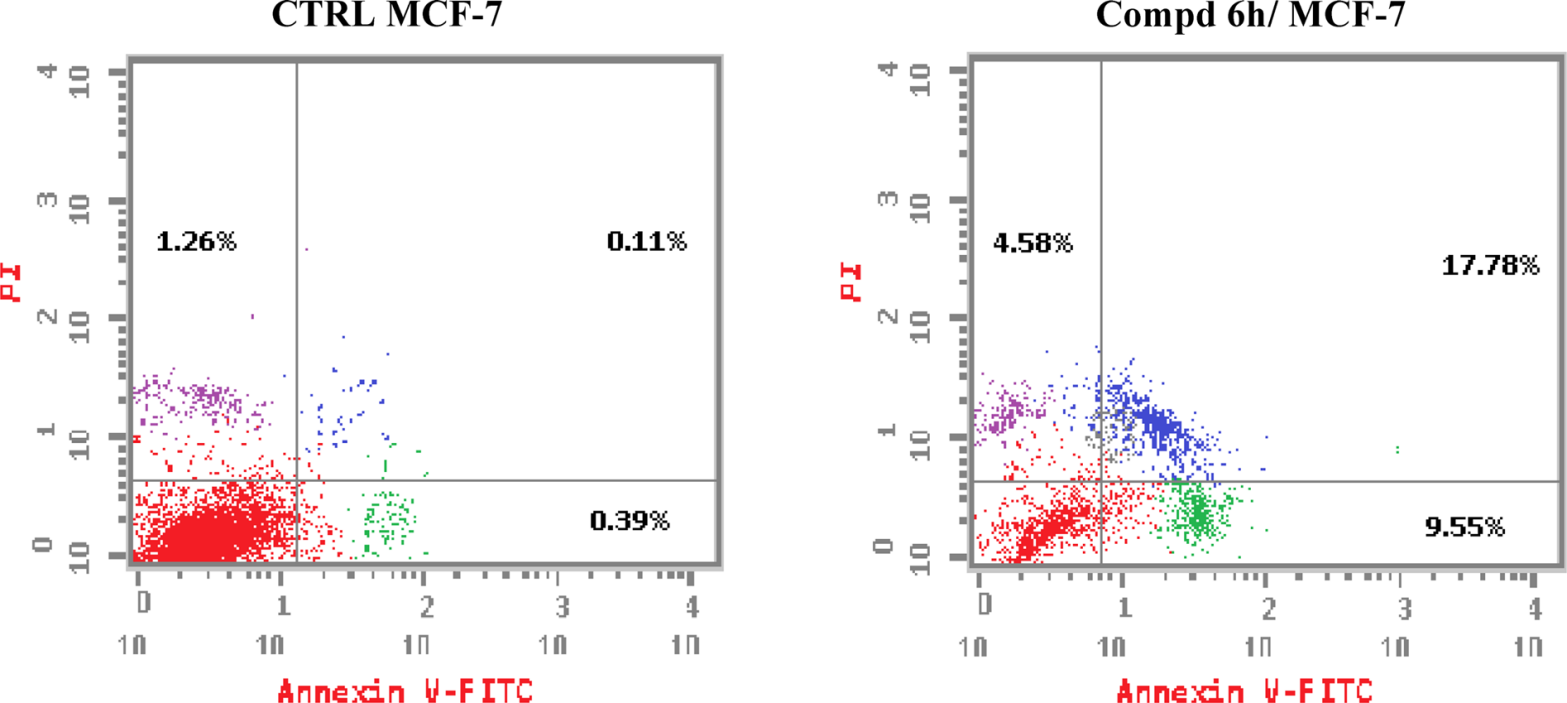
2-(Quinoline-4-carbonyl)hydrazide-3-(4-nitrophenyl)acrylamide 6h provoke apoptotic process in the examined MCF-7 cells after treatment with 2.71 μM of compound 6h and compared to DMSO controls.

To investigate whether compound 6h could induce apoptosis, the apoptosis impact of 2-(quinoline-4-carbonyl)hydrazide-3-(4-nitrophenyl)acrylamide 6h and DMSO (control untreated) were examined using Annexin V FITC/PI double colouring assay. MCF-7 cells received treatment with compound 6h at the concentration of 2.71 μM for 48 h, then labelled with Annexin V FITC and PI dyes and assessed by flow cytometry. The results of flow cytometric measurements of apoptotic distribution effect in MCF-7 cells treated with 2-(quinoline-4-carbonyl)hydrazide-3-(4-nitrophenyl)acrylamide 6h revealed that the tested compound could increase the percentage in the secondary cellular apoptosis from 0.11 to 17.78% compared to DMSO control. In addition, an increase in the primary apoptotic cells was also observed. It is noteworthy that, the percentage of primary apoptosis was rise from 0.39 to 9.55% as compared to DMSO control (Fig. 4). According to these findings, it could be concluded that this set of compounds could halt MCF-7 cells in G1 phase and provoke apoptotic cellular death.

#### 2.2.5. Apoptotic markers determination

**Fig. 5:**
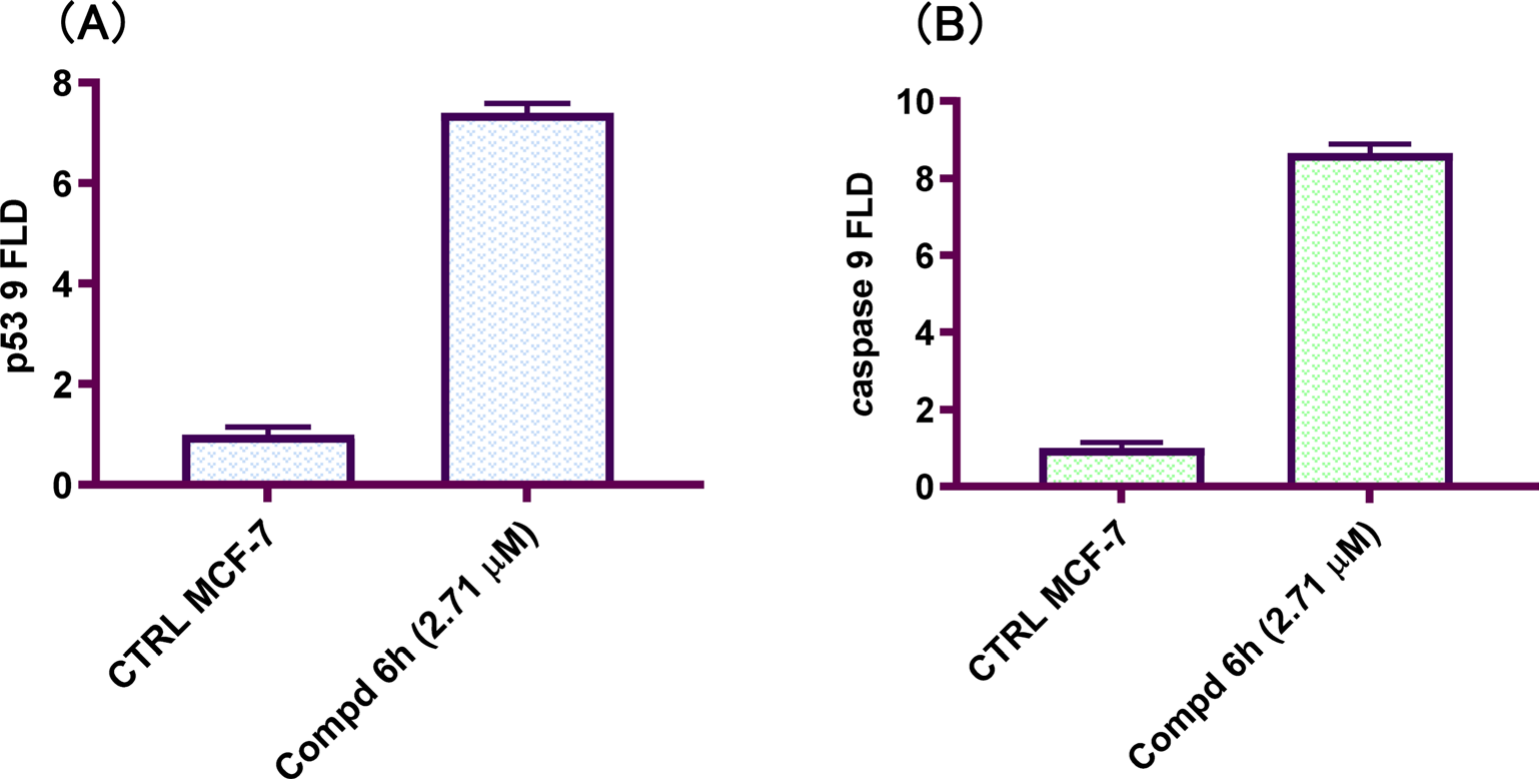
2-(Quinoline-4-carbonyl)hydrazide-3-(4-nitrophenyl)acrylamide 6h mediate p53 and caspase 9 expression. MCF-7 cells were treated with 2.71 μM of tested hybrid for 48 h before qRT-PCR measurement and compared to untreated controls. (A) p53 level; (B) caspase 9 level.

p53 is a transcription factor that both initiates and suppresses a number of target genes involved in cancer progression and apoptosis.^40^ The p53 protein level increase during cellular stress and cytotoxic drugs by increased transcription or increased stability due to post-translational modifications such as phosphorylation.^41^ The ability of p53 to modulate caspase activation and apoptosis induction is critical to its role as tumor suppressor.^42^ Coordinately, p53 activation promotes the loss of mitochondrial potential ultimately leading to caspase activation and apoptosis.^43^ To determine the downstream effects of EGFR tyrosine kinase inhibition by 2-(quinoline-4-carbonyl)hydrazide-3-(4-nitrophenyl)acrylamide 6h, the level of tumor suppressor p53 and initiator caspase 9 proteins was examined by qRT-PCR. An increase in the level of p53 was detected in the tested cell line compared with controls at 48 h. The increased expression of p53 level was coupled with increase in the level of caspase 9 in compound 6h-treated MCF-7 cell line at 48 h. The levels of p53 and caspase 9 were increased by 7.4- and 8.7-fold, respectively compared with controls (Fig. 5). In conclusions, 2-(quinoline-4-carbonyl)hydrazide-3-(4-nitrophenyl)acrylamide 6h showed a pro-apoptotic effect through increasing p53 protein level and the level of initiator caspase 9 which support the results obtained from cell cycle and apoptosis staining examinations.

## 3. Conclusion

In conclusions, a new set of 2-(quinoline-4-carbonyl)hydrazide scaffold bearing acrylamide derivatives 5 and 6a–i was synthesized and assessed for their *in vitro* antiproliferative activity against MCF-7 breast carcinoma cell line. Most tested hybrids revealed significant antiproliferative activity. Among them, three 2-(quinoline-4-carbonyl)hydrazide-acrylamide molecules; 6a, 6b and 6h presented potent antiproliferative activity against the examined cell line with IC_50_ results of 3.39, 5.94 and 2.71 μM, respectively, which were more potent than the reference compound Dox (IC_50_ = 6.18 μM). For the most potent hybrids, 2-(quinoline-4-carbonyl)hydrazide-3-(4-methylphenyl)acrylamide 6a, and 2-(quinoline-4-carbonyl)hydrazide-3-(4-nitrophenyl)acrylamide 6h were examined for their inhibitory action against EGFR kinase and results revealed the responsiveness of EGFR-kinase to the tested hybrids with IC_50_ value of 0.31 and 0.22 μM, respectively compared to Lapatinib (IC_50_ value of 0.18 μM). The cell cycle analysis gave evident that 2-(quinoline-4-carbonyl)hydrazide-3-(4-nitrophenyl)acrylamide 6h halted the MCF-7 at the G1 phase (1.3-fold more than DMSO controls) and provoke apoptosis (accumulation of cells by almost 18.1-fold compared with DMSO controls). As shown from the impacts on the gene expression of p53 and initiator caspase 9, compound 6h boosted the expression of p53 and caspase 9 by 7.4- and 8.7-fold, respectively compared to DMSO controls. On the basis of the aforementioned findings, 2-(quinoline-4-carbonyl)hydrazide-3-(4-nitrophenyl)acrylamide 6h could be considered as EGFR-TK inhibitor and fruitful clue for the development of novel antitumor agents against breast carcinoma.

## 4. Experimental

### 4.1. Chemistry

#### 4.1.1. Procedure for the synthesis of the (*Z*)-*N*-(1-aryl-3-(2-(2-(4-bromophenyl)quinoline-4-carbonyl)hydrazinyl)-3-oxoprop-1-en-2-yl)-4-methoxybenzamides 5 and 6a–i

To stirred suspension of 2-(quinoline-4-carbonyl)hydrazide 4 (3.42 g, 0.01 mol) in glacial acetic acid (25 mL), an equimolar quantity of respective ethyl (*Z*)-3-aryl-2-(4-methoxybenzamido)acrylate (0.01 mol) was refluxed along with anhydrous sodium acetate (1.07 g, 0.013 mol) for 18 h. After reaction completion, the mixture was placed into ice-cold water and vigorously agitated. Solid so obtained was filtered and rinsed three times with 5 mL of water each. The crude product was then dried in a desiccator and refined by flash chromatography with a mobile phase of ethyl acetate/*n*-hexane (4 : 1) to attain pure 2-(quinoline-4-carbonyl)hydrazide-acrylamide hybrids 5 and 6a–i.

##### 4.1.1.1. (*Z*)-*N*-(3-(2-(2-(4-Bromophenyl)quinoline-4-carbonyl)hydrazinyl)-1-(furan-2-yl)-3-oxoprop-1-en-2-yl)-4-methoxybenzamide (5)

Yield (4.40 g, 72%); m.p. 257–259 °C; C_31_H_23_BrN_4_O_5_ (611.44); ^1^H-NMR (400 MHz, DMSO-*d*_6_) *δ*: 10.79 (s, 1H, NH), 10.46 (s, 1H, NH), 9.84 (s, 1H, NH), 8.43 (d, *J* = 8.5 Hz, 1H, Ar), 8.27 (d, *J* = 8.2 Hz, 2H, Ar), 8.17 (d, *J* = 9.5 Hz, 2H, Ar), 8.08 (d, *J* = 8.2 Hz, 2H, Ar), 7.88 (t, *J* = 7.7 Hz, 1H, Ar), 7.82 (s, 2H, Ar), 7.80 (s, 1H, Ar), 7.76–7.66 (m, 1H, Ar), 7.31 (s, 1H, =CH), 7.09 (d, *J* = 8.3 Hz, 2H, Ar), 6.79 (d, *J* = 3.5 Hz, 1H, Ar), 6.63 (dd, *J* = 3.6, 1.9 Hz, 1H, Ar), 3.87 (s, 3H, Ar-OCH_3_). ^13^C-NMR (101 MHz, DMSO) *δ*: 166.11, 165.78, 164.60, 162.47, 155.01, 150.10, 148.30, 145.26, 142.01, 137.75, 132.46, 131.06, 130.42, 129.98, 129.75, 127.98, 126.56, 126.54, 126.03, 124.23, 124.06, 119.02, 117.15, 114.84, 113.99, 112.87, 55.92. MS (*m*/*z*, %): 611.50 (M^+^, 31.38), 213.48 (100). Anal. calcd (%): C, 60.89; H, 3.79; N, 9.16; found (%): C, 61.02; H, 3.93; N, 8.98.

##### 4.1.1.2. (*Z*)-*N*-(3-(2-(2-(4-Bromophenyl)quinoline-4-carbonyl)hydrazinyl)-3-oxo-1-*p*-tolylprop-1-en-2-yl)-4-methoxybenzamide (6a)

Yield (4.38 g, 69%); m.p. 246–248 °C; C_34_H_27_BrN_4_O_4_ (635.51); ^1^H-NMR (400 MHz, DMSO-*d*_6_) *δ* (ppm): 10.79 (s, 1H, NH), 10.46 (s, 1H, NH), 9.91 (s, 1H, NH), 8.45 (d, *J* = 8.4 Hz, 1H, Ar–H), 8.27 (d, *J* = 8.2 Hz, 2H, Ar–H), 8.17 (d, *J* = 8.1 Hz, 2H, Ar–H), 8.06 (d, *J* = 8.4 Hz, 2H, Ar–H), 7.88 (t, *J* = 7.7 Hz, 1H, Ar–H), 7.81 (d, *J* = 8.0 Hz, 2H, Ar–H), 7.72 (t, *J* = 7.8 Hz, 1H, Ar–H), 7.55 (d, *J* = 7.8 Hz, 2H, Ar–H), 7.39 (s, 1H, =CH), 7.21 (d, *J* = 7.8 Hz, 2H, Ar–H), 7.09 (d, *J* = 8.3 Hz, 2H, Ar–H), 3.86 (s, 3H, Ar-OCH_3_), 2.31 (s, 3H, Ar-CH_3_). ^13^C-NMR (101 MHz, DMSO) *δ*: 166.15, 165.97, 165.30, 162.48, 155.01, 148.31, 142.09, 139.17, 137.77, 132.46, 131.72, 131.05, 130.92, 130.40, 130.03, 129.98, 129.74, 129.66, 128.52, 127.96, 126.43, 126.05, 124.23, 124.09, 117.14, 113.99, 55.92, 21.40. MS (*m*/*z*, %): 635.98 (M^+^, 30.36), 335.21 (100). Anal. calcd (%): C, 64.26; H, 4.28; N, 8.82; found (%): C, 64.39; H, 4.17; N, 8.89.

##### 4.1.1.3. (*Z*)-*N*-(3-(2-(2-(4-Bromophenyl)quinoline-4-carbonyl)hydrazinyl)-1-(3,4-dimethoxyphenyl)-3-oxoprop-1-en-2-yl)-4-methoxybenzamide (6b)

Yield (4.56 g, 67%); m.p. 235–237 °C; C_35_H_29_BrN_4_O_6_ (681.53); ^1^H-NMR (400 MHz, DMSO-*d*_6_) *δ* 10.76 (s, 1H, NH), 10.40 (s, 1H, NH), 9.89 (s, 1H, NH), 8.45 (d, *J* = 8.4 Hz, 1H), 8.34–8.21 (m, 3H), 8.16 (d, *J* = 9.3 Hz, 1H), 7.88 (ddd, *J* = 8.4, 6.9, 1.5 Hz, 1H), 7.84–7.78 (m, 1H), 7.75–7.66 (m, 2H), 7.66–7.61 (m, 1H), 7.56 (d, *J* = 2.0 Hz, 1H), 7.39 (s, 1H, =CH), 7.24 (s, 1H), 7.20 (d, *J* = 8.6 Hz, 1H), 7.15–7.08 (m, 2H), 7.02–6.95 (m, 1H), 3.90 (s, 3H, OCH_3_), 3.89 (s, 3H, OCH_3_), 3.86 (s, 3H, OCH_3_). ^13^C-NMR (101 MHz, DMSO) *δ*: 167.74, 166.17, 166.04, 162.13, 160.31, 155.01, 153.83, 152.17, 149.50, 148.66, 137.76, 134.80, 132.46, 131.83, 131.29, 130.23, 129.73, 126.77, 126.06, 124.22, 122.65, 121.94, 117.76, 115.13, 114.59, 112.39, 111.38, 110.35, 56.34, 55.96, 55.72. MS (*m*/*z*, %): 681.38 (M^+^, 23.82), 84.38 (100). Anal. calcd (%): C, 61.68; H, 4.29; N, 8.22; found (%): C, 61.63; H, 4.16; N, 8.35.

##### 4.1.1.4. (*Z*)-*N*-(3-(2-(2-(4-Bromophenyl)quinoline-4-carbonyl)hydrazinyl)-3-oxo-1-(2,3,4-trimethoxyphenyl)prop-1-en-2-yl)-4-methoxybenzamide (6c)

Yield (4.12 g, 58%); m.p. 192–194 °C; C_36_H_31_BrN_4_O_7_ (711.56); ^1^H-NMR (400 MHz, DMSO-*d*_6_) *δ* 10.75 (s, 1H), 10.39 (s, 1H), 9.87 (s, 1H), 8.43 (d, *J* = 8.4 Hz, 1H), 8.29–8.26 (m, 1H), 8.15 (s, 1H), 8.06 (dd, *J* = 8.7, 6.2 Hz, 2H), 7.87 (t, *J* = 7.7 Hz, 1H), 7.81–7.79 (m, 1H), 7.71 (t, *J* = 7.8 Hz, 1H), 7.61 (d, *J* = 8.9 Hz, 2H), 7.39 (s, 1H), 7.20–7.17 (m, 1H), 7.10–7.07 (m, 2H), 7.01–6.90 (m, 2H), 3.89 (s, 3H, OCH_3_), 3.86 (s, 6H, 2OCH_3_), 3.78 (s, 3H, OCH_3_). MS (*m*/*z*, %): 711.05 (M^+^, 34.65), 252.08 (100). Anal. calcd (%): C, 60.988; H, 4.39; N, 7.87; found (%): C, 60.94; H, 4.26; N, 7.71.

##### 4.1.1.5. (*Z*)-*N*-(3-(2-(2-(4-Bromophenyl)quinoline-4-carbonyl)hydrazinyl)-3-oxo-1-(3,4,5-trimethoxyphenyl)prop-1-en-2-yl)-4-methoxybenzamide (6d)

Yield (4.55 g, 64%); m.p. 208–210 °C; C_36_H_31_BrN_4_O_7_ (711.56); ^1^H-NMR (400 MHz, DMSO-*d*_6_) *δ* 10.77 (s, 1H, NH), 10.46 (s, 1H, NH), 9.92 (s, 1H, NH), 8.44 (d, *J* = 8.4 Hz, 1H), 8.28–8.23 (m, 2H), 8.18–8.13 (m, 2H), 8.08 (d, *J* = 8.5 Hz, 2H), 7.87 (ddd, *J* = 8.4, 6.8, 1.5 Hz, 1H), 7.83–7.78 (m, 2H), 7.70 (ddd, *J* = 8.2, 6.8, 1.2 Hz, 1H), 7.43 (s, 1H, =CH), 7.09–7.04 (m, 2H), 7.02 (s, 2H), 3.84 (s, 3H, OCH_3_), 3.67 (s, 3H, OCH_3_), 3.63 (s, 6H, 2OCH_3_). ^13^C-NMR (101 MHz, DMSO) *δ*: 166.11, 165.99, 165.09, 162.49, 155.01, 153.07, 148.31, 142.09, 138.65, 137.77, 132.46, 131.50, 131.06, 130.37, 129.98, 129.82, 129.75, 128.41, 127.94, 126.30, 126.06, 124.22, 124.09, 117.12, 113.91, 107.70, 60.54, 56.09, 55.91. MS (*m*/*z*, %): 711.38 (M^+^, 19.20), 95.23 (100). Anal. calcd (%): C, 60.77; H, 4.39; N, 7.87; found (%): C, 60.94; H, 4.51; N, 7.66.

##### 4.1.1.6. (*Z*)-*N*-(3-(2-(2-(4-Bromophenyl)quinoline-4-carbonyl)hydrazinyl)-1-(4-fluorophenyl)-3-oxoprop-1-en-2-yl)-4-methoxybenzamide (6e)

Yield (4.86 g, 76%); m.p. 258–260 °C; C_33_H_24_BrFN_4_O_4_ (639.47); ^1^H-NMR (400 MHz, DMSO-*d*_6_) *δ* 10.78 (s, 1H, NH), 10.50 (s, 1H, NH), 9.95 (s, 1H, NH), 8.45 (d, *J* = 8.4 Hz, 1H), 8.30–8.24 (m, 2H), 8.17 (d, *J* = 8.1 Hz, 2H), 8.05 (d, *J* = 8.5 Hz, 2H), 7.88 (ddd, *J* = 8.5, 6.8, 1.4 Hz, 1H), 7.84–7.78 (m, 2H), 7.73 (d, *J* = 2.9 Hz, 1H), 7.71 (s, 1H), 7.69 (dd, *J* = 3.6, 2.3 Hz, 1H), 7.40 (s, 1H, =CH), 7.29–7.22 (m, 2H), 7.12–7.05 (m, 2H), 3.86 (s, 3H, OCH_3_). ^13^C-NMR (101 MHz, DMSO) *δ*: 166.11, 166.01, 165.10, 162.54, 161.29, 155.01, 148.31, 142.11, 137.77, 132.45, 132.12, 131.15, 131.04, 130.44, 129.98, 129.74, 129.52, 129.17, 127.95, 126.27, 126.06, 124.22, 124.09, 117.13, 116.16, 114.00, 55.93. MS (*m*/*z*, %): 639.18 (M^+^, 23.38), 486.00 (100). Anal. calcd (%): C, C, 61.98; H, 3.78; N, 8.76; found (%): C, 62.09; H, 3.88; N, 8.67.

##### 4.1.1.7. (*Z*)-*N*-(3-(2-(2-(4-Bromophenyl)quinoline-4-carbonyl)hydrazinyl)-1-(4-chlorophenyl)-3-oxoprop-1-en-2-yl)-4-methoxybenzamide (6f)

Yield (5.37 g, 82%); m.p. 276–278 °C; C_33_H_24_BrClN_4_O_4_ (655.93); ^1^H-NMR (400 MHz, DMSO-*d*_6_) *δ* 10.81 (s, 1H, NH), 10.54 (s, 1H, NH), 9.98 (s, 1H, NH), 8.44 (dd, *J* = 8.4, 1.4 Hz, 1H), 8.27 (d, *J* = 8.5 Hz, 2H), 8.17 (d, *J* = 8.5 Hz, 2H), 8.07–8.01 (m, 2H), 7.88 (t, *J* = 6.8 Hz, 1H), 7.80 (dd, *J* = 8.9, 2.3 Hz, 2H), 7.71 (t, *J* = 6.8 Hz, 1H), 7.68–7.63 (m, 2H), 7.51–7.44 (m, 2H), 7.36 (s, 1H, =CH), 7.12–7.05 (m, 2H), 3.86 (s, 3H, OCH_3_). ^13^C-NMR (101 MHz, DMSO) *δ*: 166.13, 165.98, 165.10, 162.57, 155.01, 148.30, 142.01, 137.75, 133.74, 133.53, 132.45, 131.59, 131.06, 130.46, 130.10, 129.98, 129.75, 129.10, 129.06, 127.98, 126.21, 126.01, 124.23, 124.07, 117.14, 114.02, 55.93. MS (*m*/*z*, %): 655.05 (M^+^, 10.76), 75.80 (100). Anal. calcd (%): C, 60.43; H, 3.69; N, 8.54; found (%): C, 60.40; H, 3.84; N, 8.68.

##### 4.1.1.8. (*Z*)-*N*-(1-(4-Bromophenyl)-3-(2-(2-(4-bromophenyl)quinoline-4-carbonyl)hydrazinyl)-3-oxoprop-1-en-2-yl)-4-methoxybenzamide (6g)

Yield (5.53 g, 79%); m.p. 261–263 °C; C_33_H_24_Br_2_N_4_O_4_ (700.38); ^1^H-NMR (400 MHz, DMSO-*d*_6_) *δ* 10.81 (s, 1H, NH), 10.54 (s, 1H, NH), 9.97 (s, 1H, NH), 8.43 (d, *J* = 8.4 Hz, 1H), 8.27 (d, *J* = 8.4 Hz, 2H), 8.17 (d, *J* = 8.6 Hz, 2H), 8.03 (d, *J* = 8.4 Hz, 2H), 7.88 (t, *J* = 7.7 Hz, 1H), 7.81 (d, *J* = 8.2 Hz, 2H), 7.72 (q, *J* = 8.4, 7.5 Hz, 2H), 7.60 (t, *J* = 6.7 Hz, 3H), 7.33 (s, 1H, =CH), 7.08 (d, *J* = 8.4 Hz, 2H), 3.86 (s, 3H, OCH_3_). ^13^C-NMR (101 MHz, DMSO) *δ*: 166.13, 165.98, 165.10, 162.57, 155.01, 148.30, 142.01, 137.75, 133.74, 133.53, 132.45, 131.59, 131.06, 130.46, 130.10, 129.98, 129.75, 129.10, 129.06, 127.98, 126.21, 126.01, 124.23, 124.07, 117.14, 114.02, 55.93. MS (*m*/*z*, %): 700.27 (M^+^, 13.24), 509.29 (100). Anal. calcd (%): C, 56.59; H, 3.45; N, 8.00; found (%): C, 56.48; H, 3.31; N, 7.86.

##### 4.1.1.9. (*Z*)-*N*-(3-(2-(2-(4-Bromophenyl)quinoline-4-carbonyl)hydrazinyl)-1-(4-nitrophenyl)-3-oxoprop-1-en-2-yl)-4-methoxybenzamide (6h)

Yield (5.33 g, 80%); m.p. 263–265 °C; C_33_H_24_BrN_5_O_6_ (666.48); ^1^H-NMR (400 MHz, DMSO-*d*_6_) *δ* 10.88 (s, 1H, NH), 10.68 (s, 1H, NH), 10.14 (s, 1H, NH), 8.54 (d, *J* = 8.5 Hz, 1H), 8.43 (d, *J* = 8.3 Hz, 1H), 8.35 (d, *J* = 8.5 Hz, 1H), 8.29–8.24 (m, 3H), 8.17 (d, *J* = 7.2 Hz, 2H), 8.02 (d, *J* = 8.4 Hz, 2H), 7.87 (d, *J* = 8.2 Hz, 2H), 7.81 (d, *J* = 8.2 Hz, 2H), 7.72 (t, *J* = 7.8 Hz, 1H), 7.38 (s, 1H, =CH), 7.08 (d, *J* = 8.4 Hz, 2H), 3.86 (s, 3H, OCH_3_). ^13^C-NMR (101 MHz, DMSO) *δ*: 166.13, 165.99, 164.88, 162.69, 155.02, 148.30, 147.17, 141.90, 141.66, 137.74, 132.83, 132.46, 131.08, 130.79, 130.54, 130.00, 129.75, 128.01, 127.04, 125.99, 124.24, 124.15, 124.04, 117.16, 115.55, 114.06, 55.96. MS (*m*/*z*, %): 666.71 (M^+^, 11.06), 552.28 (100). Anal. calcd (%): C, 59.47; H, 3.63; N, 10.51; found (%): C, 59.38; H, 3.74; N, 10.59.

##### 4.1.1.10. (*Z*)-2,6-Dibromo-4-(3-(2-(2-(4-bromophenyl)quinoline-4-carbonyl)hydrazinyl)-2-(4-methoxybenzamido)-3-oxoprop-1-enyl)phenyl acetate (6i)

Yield (5.94 g, 71%); m.p. 217–219 °C; C_35_H_25_Br_3_N_4_O_6_ (837.31); ^1^H NMR (400 MHz, DMSO-*d*_6_) *δ* 10.85 (s, 1H, NH), 10.62 (s, 1H, NH), 10.08 (s, 1H, NH), 8.44 (d, *J* = 8.4 Hz, 1H), 8.27 (dd, *J* = 8.6, 2.5 Hz, 2H), 8.17 (d, *J* = 7.9 Hz, 2H), 8.04 (s, 1H), 8.02 (d, *J* = 3.7 Hz, 3H), 7.88 (ddd, *J* = 8.5, 6.7, 1.5 Hz, 1H), 7.83–7.79 (m, 2H), 7.72 (t, *J* = 7.7 Hz, 1H), 7.35 (s, 1H), 7.09 (dd, *J* = 9.1, 2.5 Hz, 2H), 3.86 (s, 3H, OCH_3_), 2.41 (s, 3H, COCH_3_). ^13^C-NMR (101 MHz, DMSO) *δ*: 167.67, 166.22, 166.09, 164.60, 162.64, 155.02, 148.31, 145.70, 141.93, 137.75, 135.74, 133.45, 132.45, 131.75, 131.06, 130.41, 130.00, 129.75, 128.00, 126.84, 126.08, 125.98, 124.24, 124.06, 117.63, 117.15, 114.05, 55.94, 20.66. MS (*m*/*z*, %): 837.34 (M^+^, 17.66), 238.16 (100). Anal. calcd (%): C, 50.21; H, 3.01; N, 6.69; found (%): C, 50.04; H, 2.91; N, 6.83.

### 4.2. Biological studies

All the experimental procedure used in the biological studies were shown in the ESI.†

## Data availability

The authors agree to reproduce any published material (figures, schemes, tables, or any extract of a text) that does not fall into the public domain, or for which they do not hold the copyright.

## Conflicts of interest

No potential conflict of interest was reported by the author(s).

## Supplementary Material

RA-014-D4RA03963G-s001
