# Supplementary material for: Synthesis, characterization and biological research of novel 2-(quinoline-4-carbonyl)hydrazide-acrylamide hybrids as potential anticancer agents on MCF-7 breast carcinoma cells by targeting EGFR-TK
Source: RSC Adv. 2024 Jul 26;14(32):23495–504. doi: 10.1039/d4ra03963g (PMC11273260; doi:10.1039/d4ra03963g)
Supplement: RA-014-D4RA03963G-s001 [file RA-014-D4RA03963G-s001.pdf]

Electronic Supplementary Material (ESI) for RSC Advances.
This journal is © The Royal Society of Chemistry 2024

**Synthesis, Characterization and Biological Research of Novel 2-(Quinoline-4-carbonyl)hydrazide-acrylamide Hybrids as Potential Anticancer Agents on MCF-7 Breast Carcinoma Cells by targeting EGFR-TK**

Hany M. Abd El-Lateef [1,2,*], Duaa Abdullah Bafail [3], Noura hamdi yousef alhalees [4], Eslam E. M. Toson [5], Ali H. Abu Almaaty [6], El-Sherbiny H. El-Sayed [5], Islam Zaki [7,*], and Magdy M. Youssef [8]

[1] *Department of Chemistry, College of Science, King Faisal University, Al-Ahsa 31982, Saudi Arabia*

[2] *Department of Chemistry, Faculty of Science, Sohag University, Sohag 82524, Egypt, hmahmed@kfu.edu.sa*

[3] *Department of Clinical Pharmacology, Faculty of Medicine, King Abdulaziz University, Jeddah, Saudi Arabia*

[4] *Pharma. D (drug information supervisor) Ministry of health king abdullah medical complex Jeddah, Saudi Arabia*

[5] *Chemistry Department, Faculty of Science, Port Said University, Port Said 42526, Egypt*

[6] *Zoology Department, Faculty of Science, Port Said University, Port Said 42526, Egypt*

[7] *Pharmaceutical Organic Chemistry Department, Faculty of pharmacy, Port Said University, Port Said, Egypt, Eslam.Zaki@pharm.psu.edu.eg*

[8] *Biochemistry Division, Chemistry Department, Faculty of Science, Mansoura University, Mansoura, Egypt*

---

* *To whome correspondence should be addressed*

Islam Zaki, PhD. Pharmaceutical Organic Chemistry Department, Faculty of pharmacy, Port Said University, Port Said, Egypt.

***E-mail address***: Eslam.Zaki@pharm.psu.edu.eg **(I. Zaki)**

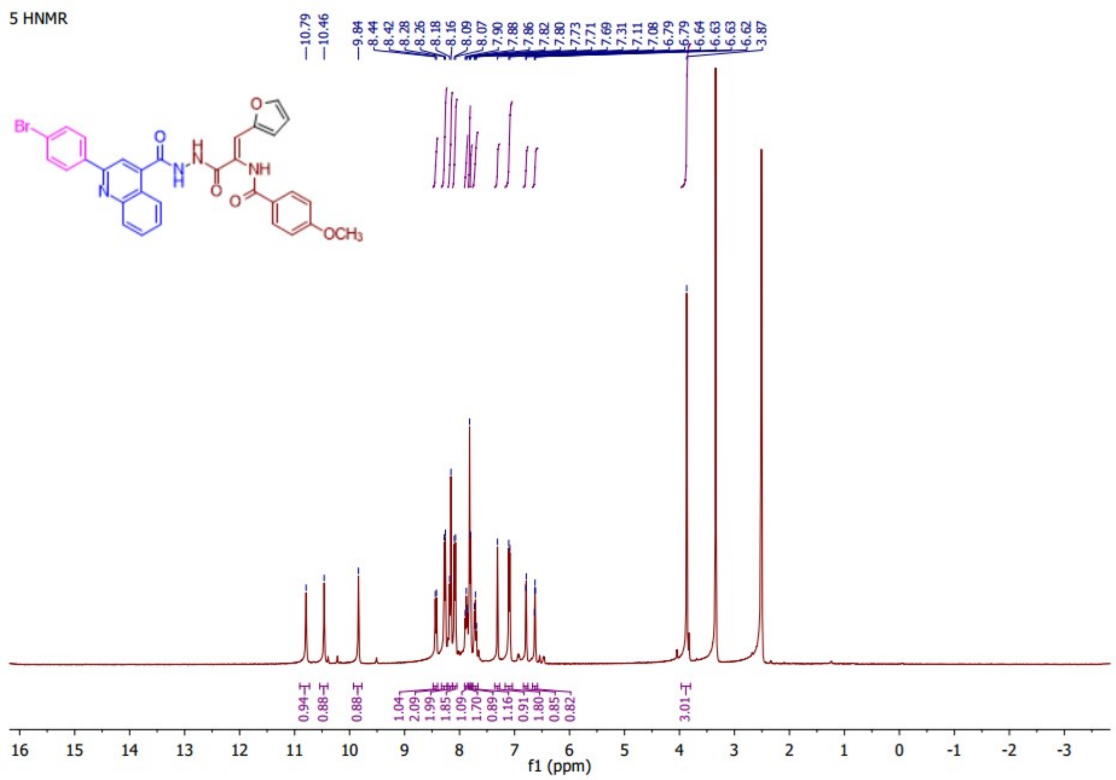

**Figure S1:** $^{1}$H-NMR spectrum of compound **5**

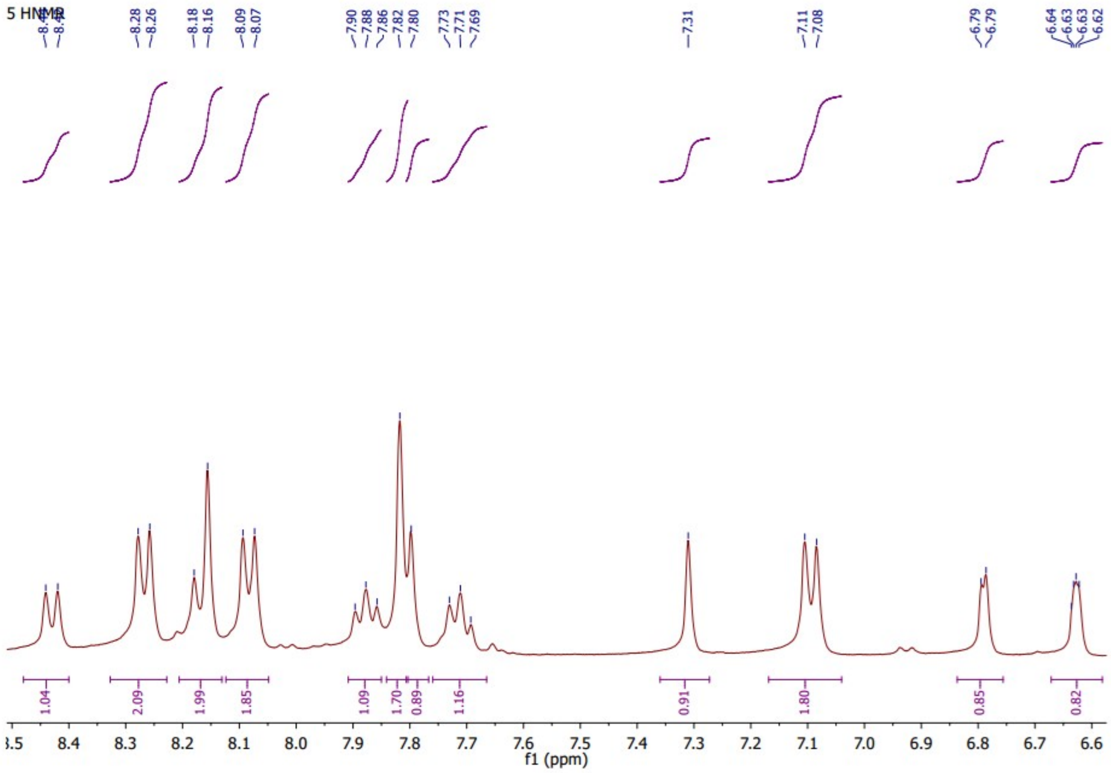

**Figure S2:** $^{1}$H-NMR spectrum of compound **5 (**zoom-in window**)**

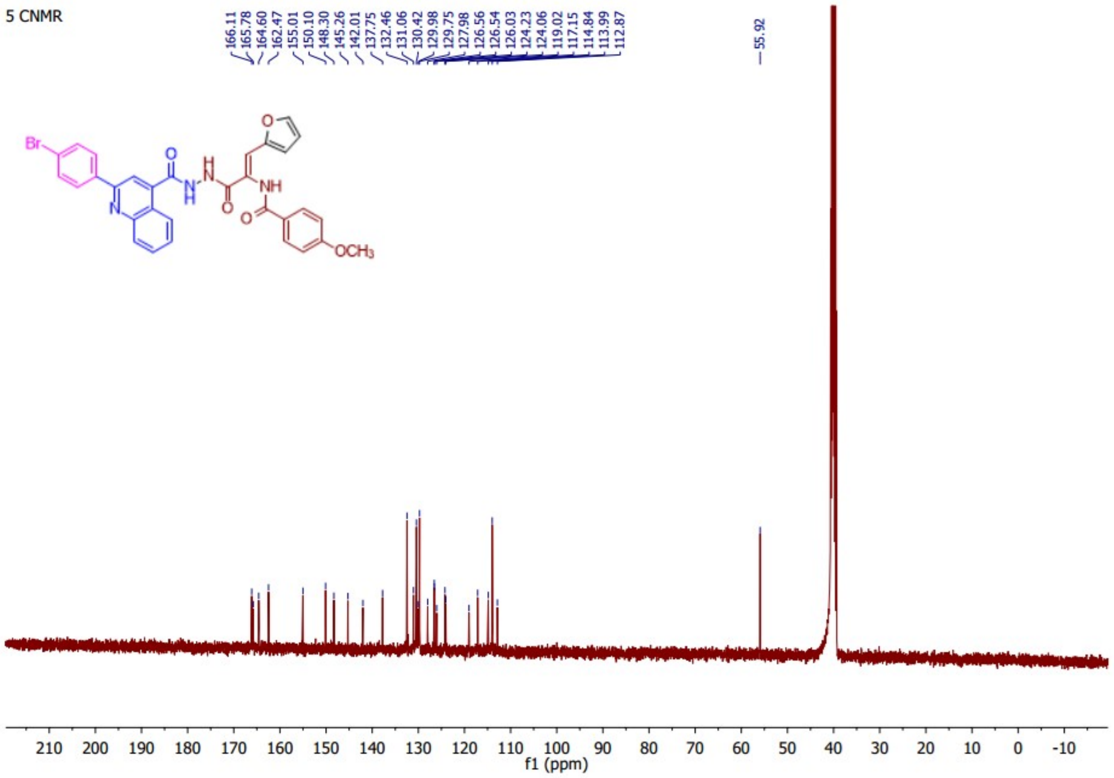


**Figure S3:** $^{13}$C-NMR spectrum of compound **5**

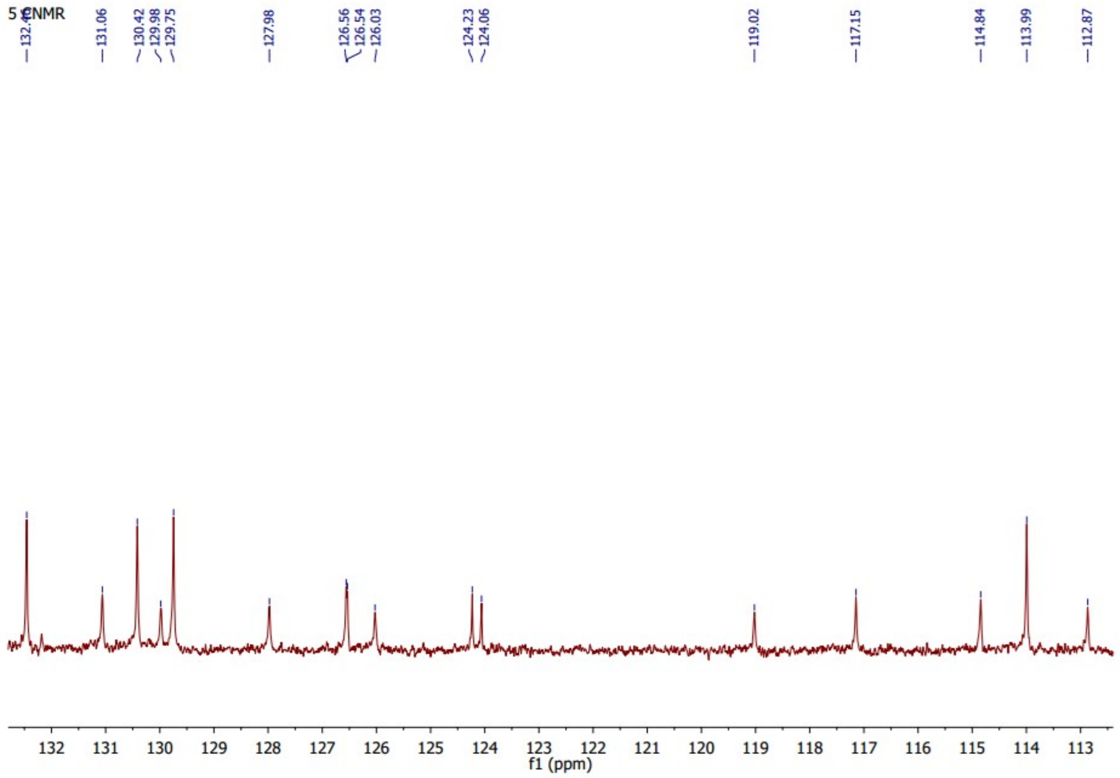


**Figure S4:** $^{13}$C-NMR spectrum of compound **5** (zoom-in window)

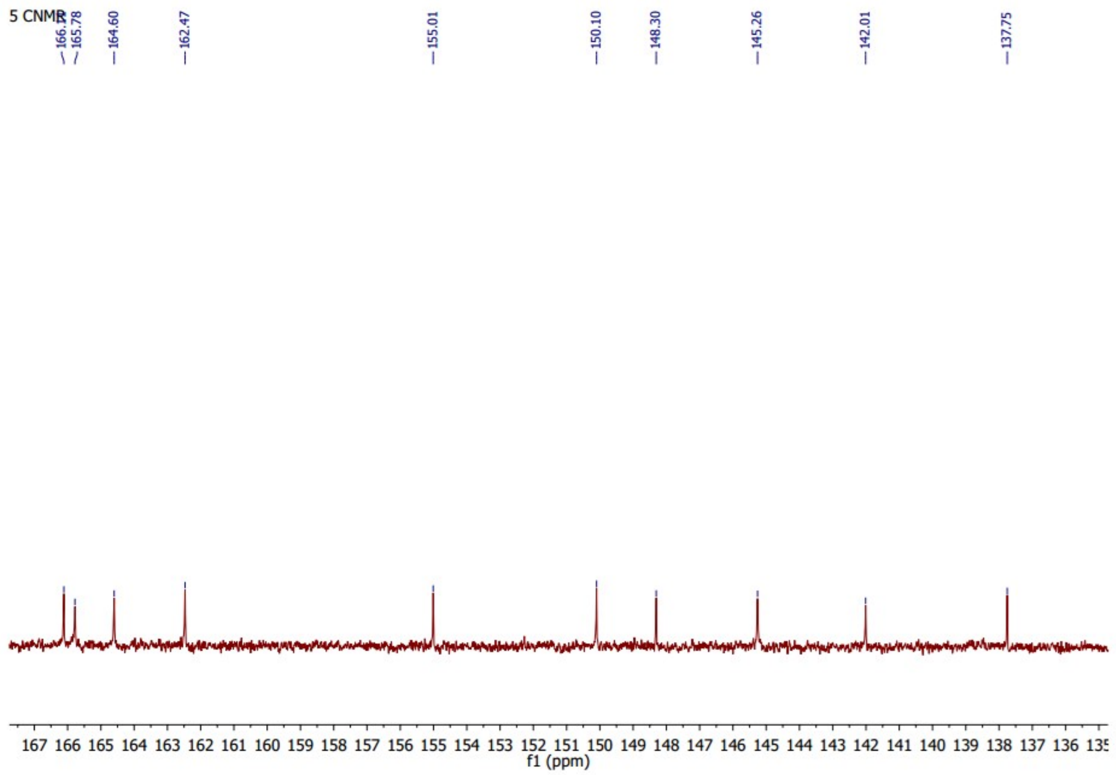


**Figure S5:** $^{13}$C-NMR spectrum of compound **5 (**zoom-in window**)**

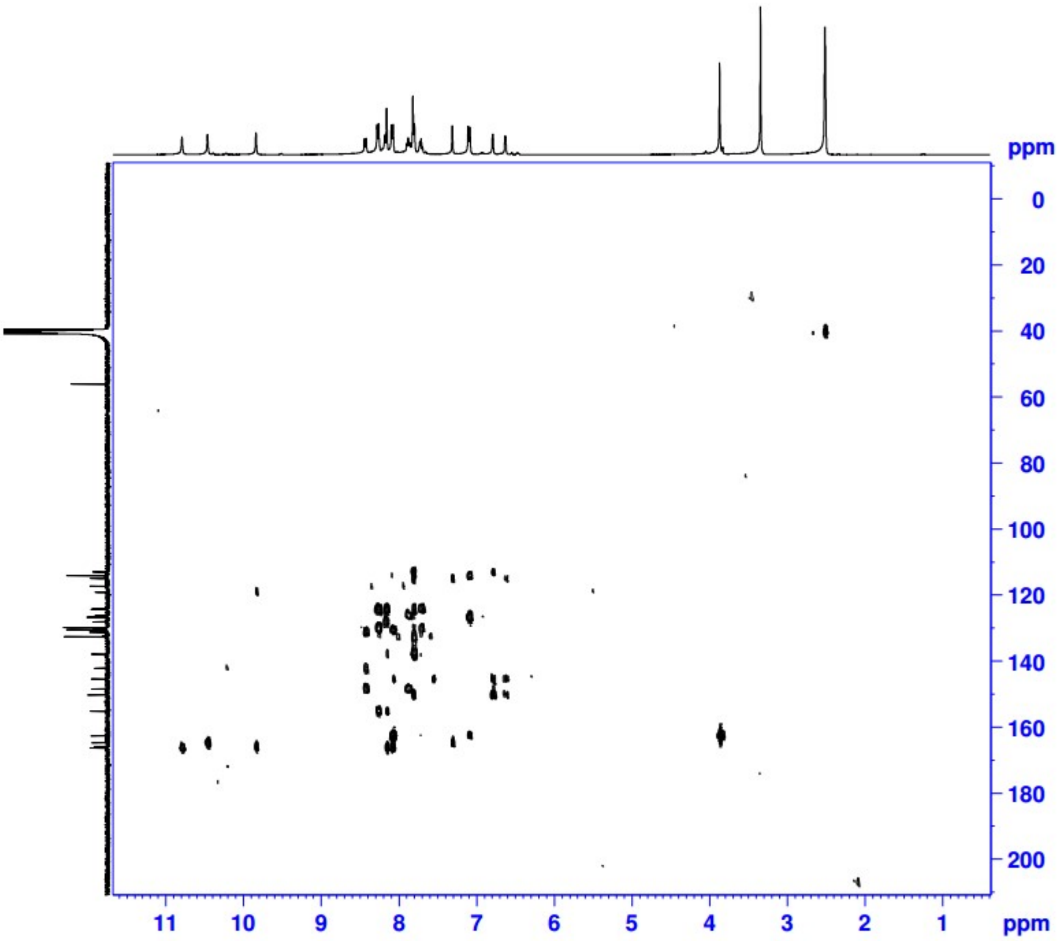


**Figure S6:** HMBC correlation spectrum of compound **5**

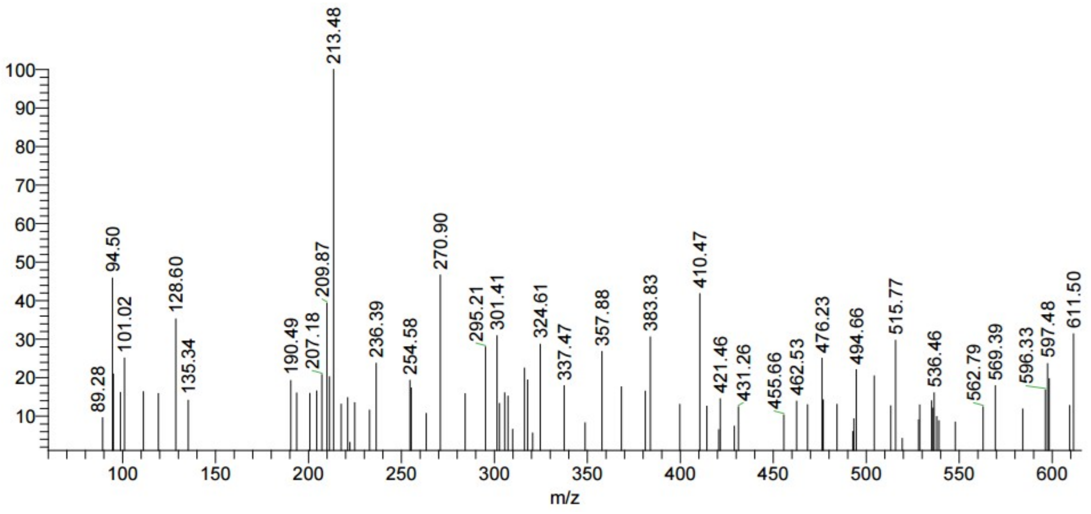


**Figure S7:** Mass spectrum of compound **5**

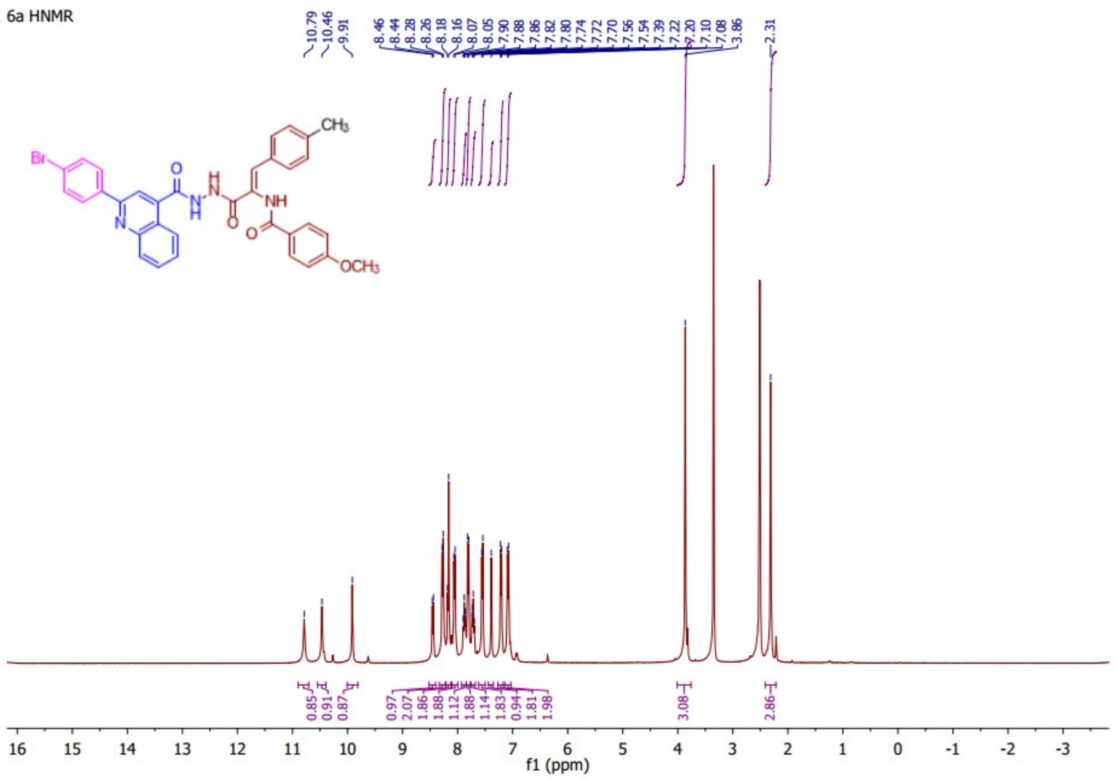


**Figure S8:** $^{1}$H-NMR spectrum of compound **6a**

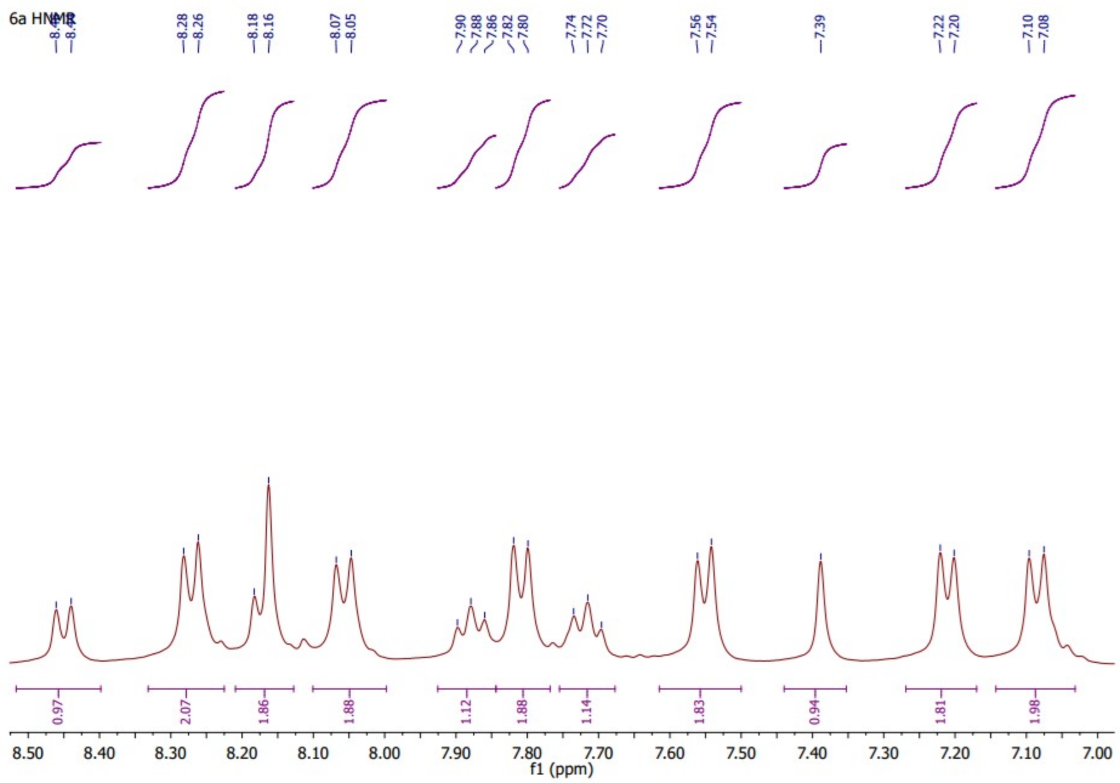

**Figure S9:** $^{1}$H-NMR spectrum of compound **6a (**zoom-in window**)**

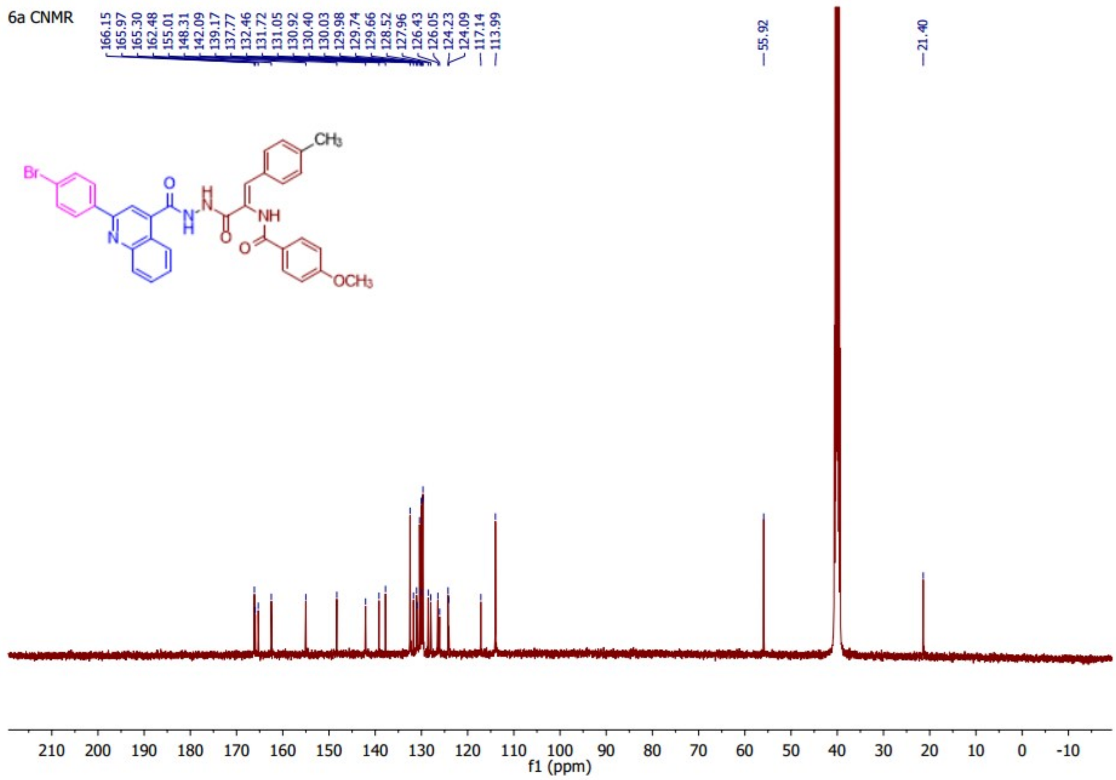


**Figure S10:** $^{13}$C-NMR spectrum of compound **6a**

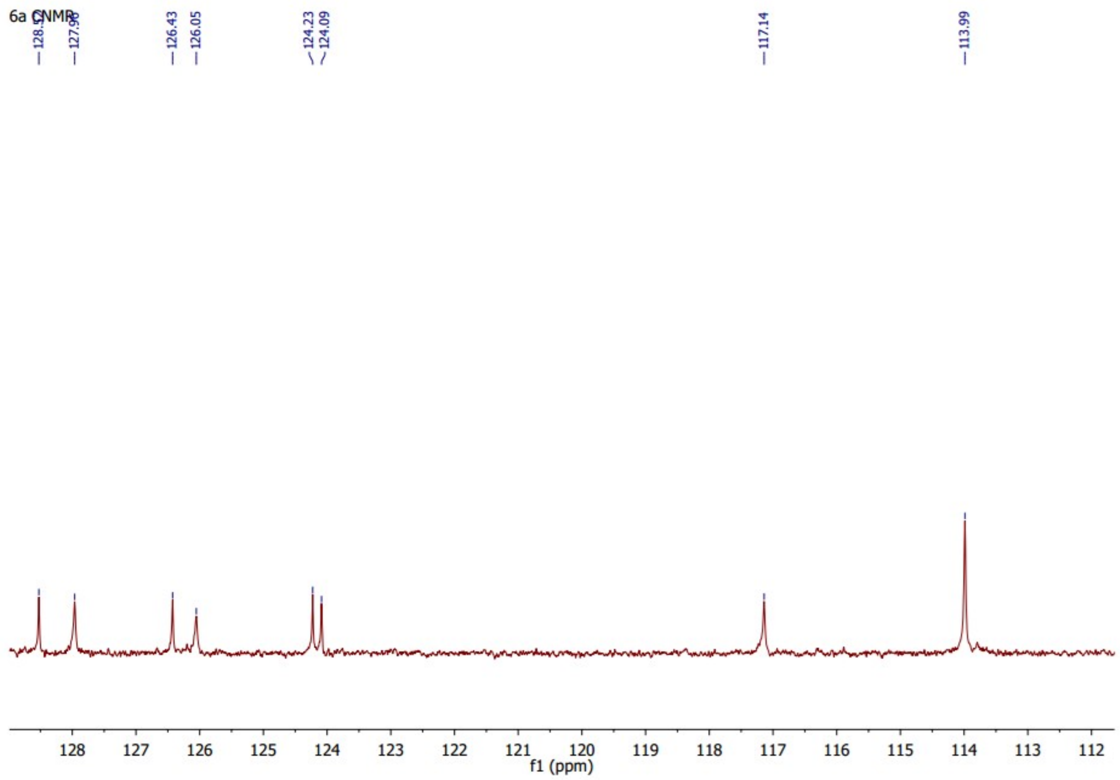


**Figure S11:** $^{13}$C-NMR spectrum of compound **6a (**zoom-in window**)**

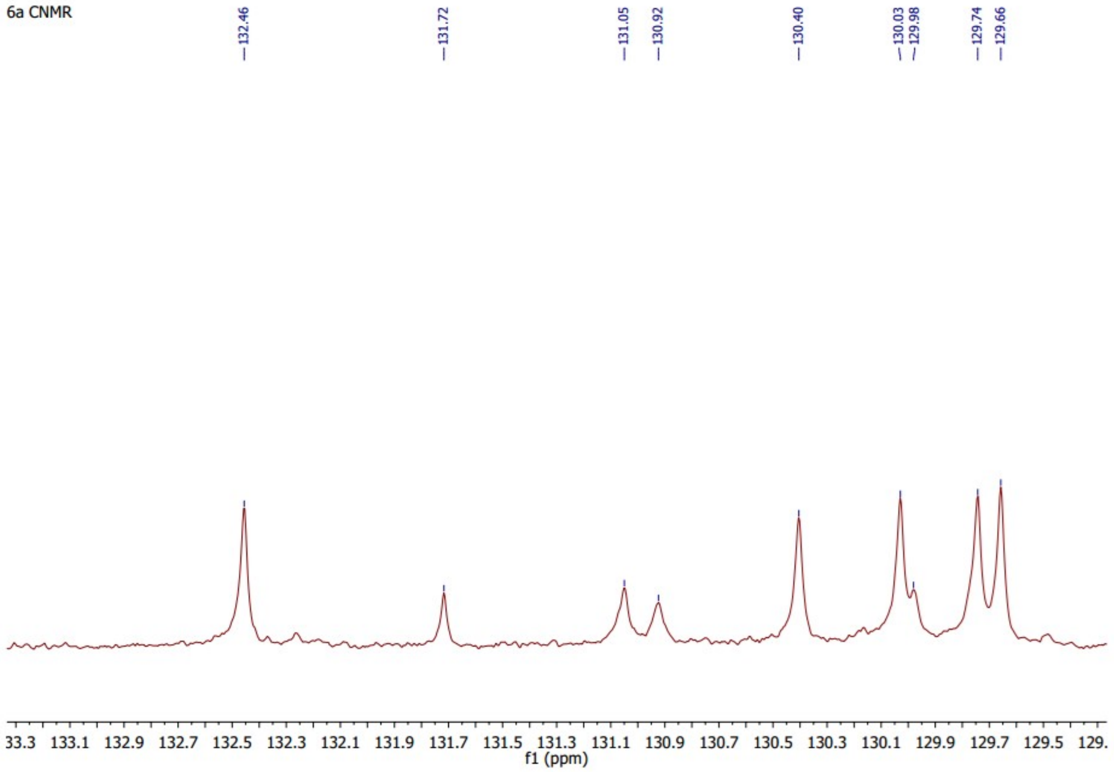


**Figure S12:** $^{13}$C-NMR spectrum of compound **6a (**zoom-in window**)**

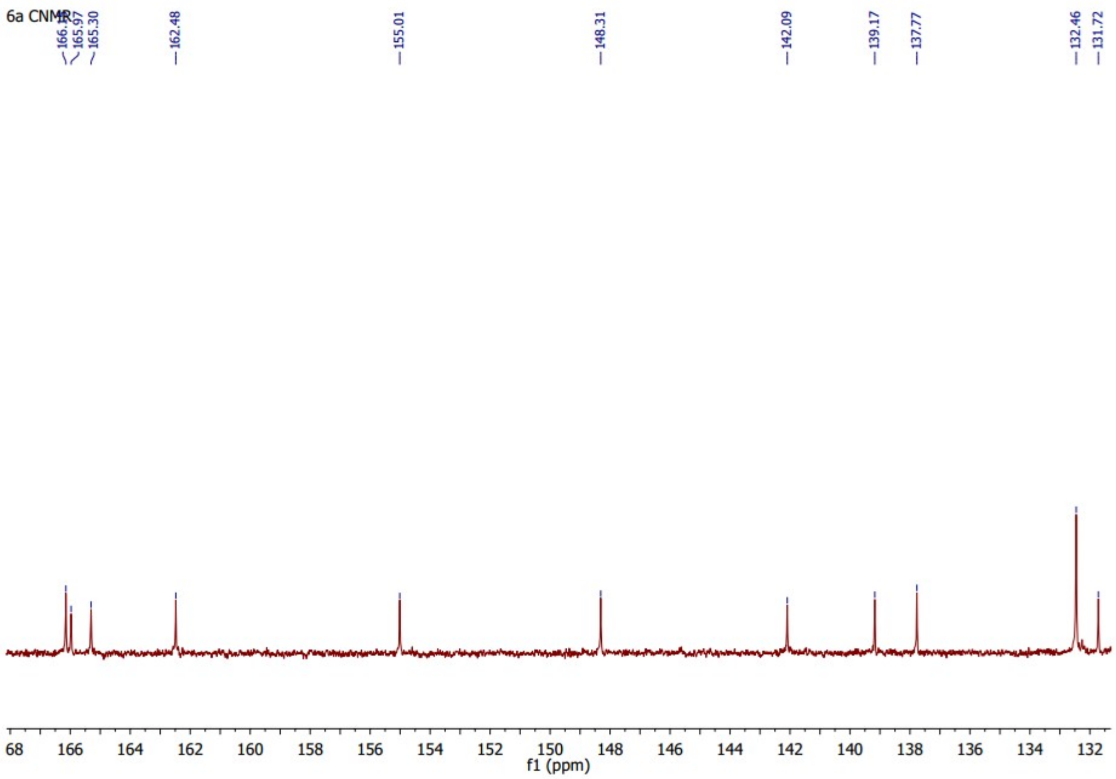


**Figure S13:** $^{13}$C-NMR spectrum of compound **6a** (zoom-in window)

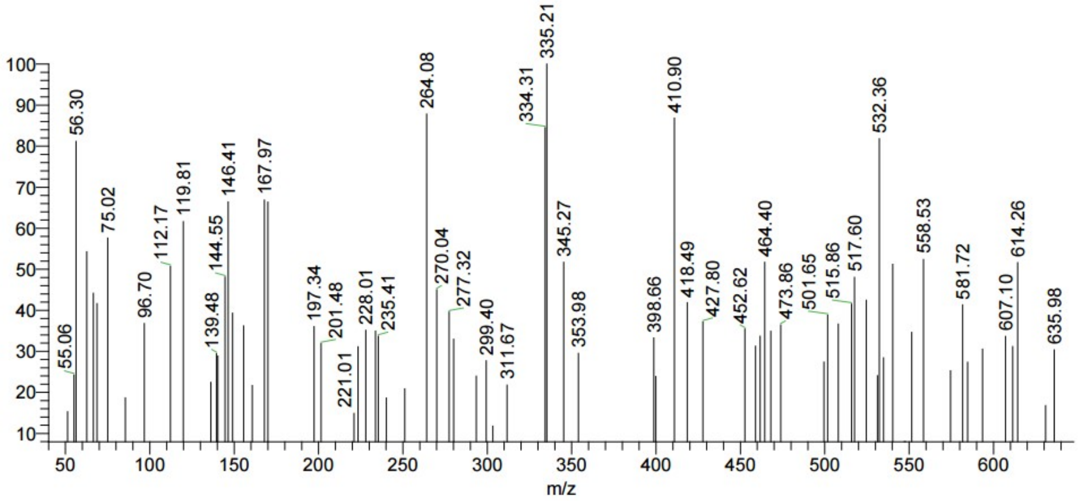


**Figure S14:** Mass spectrum of compound **6a**

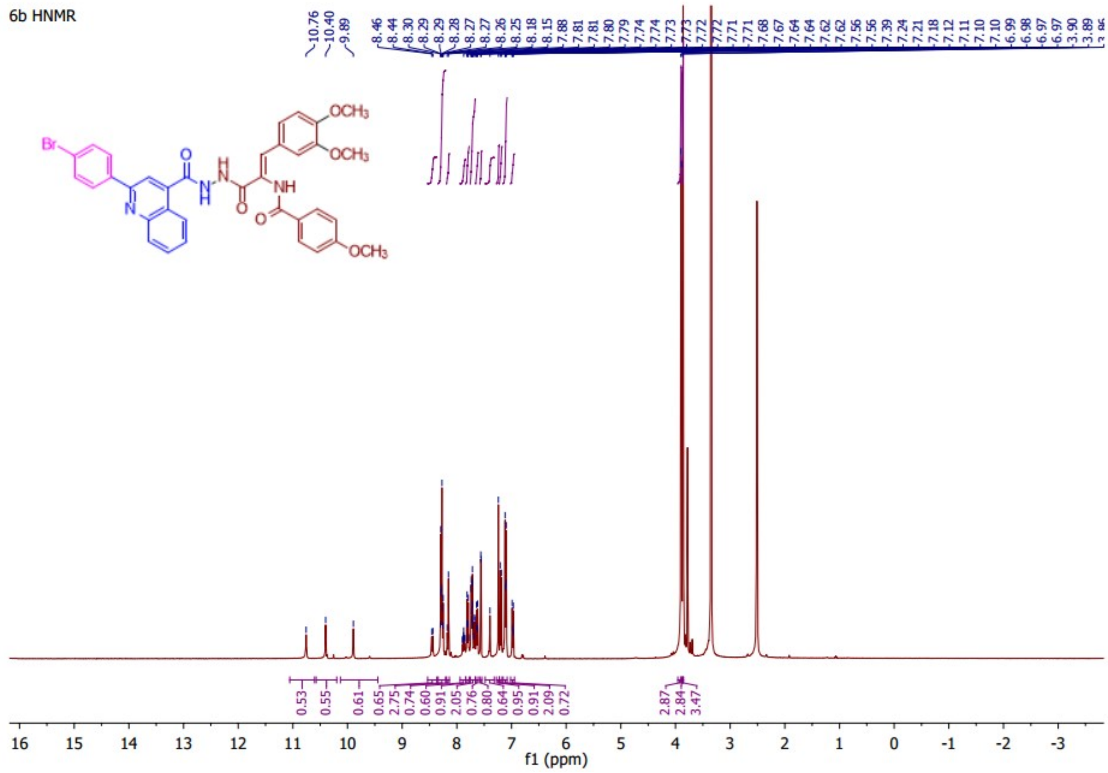

**Figure S15:** $^{1}$H-NMR spectrum of compound **6b**

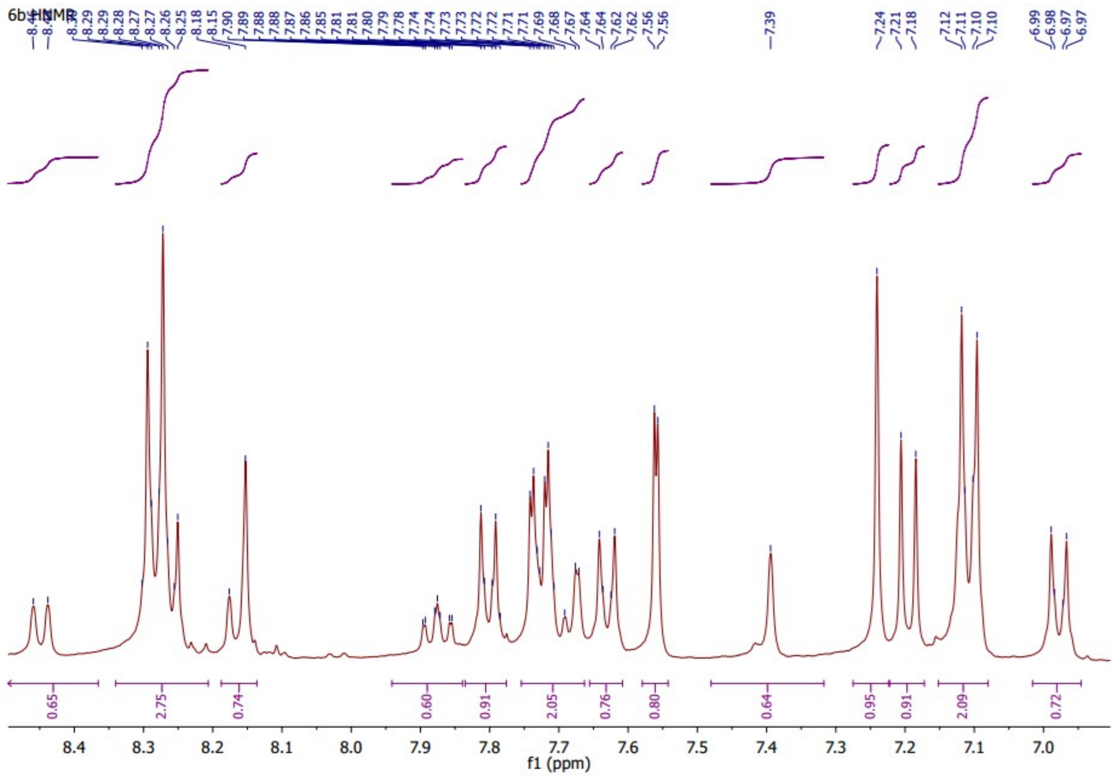

**Figure S16:** $^1$H-NMR spectrum of compound **6b** (zoom-in window)

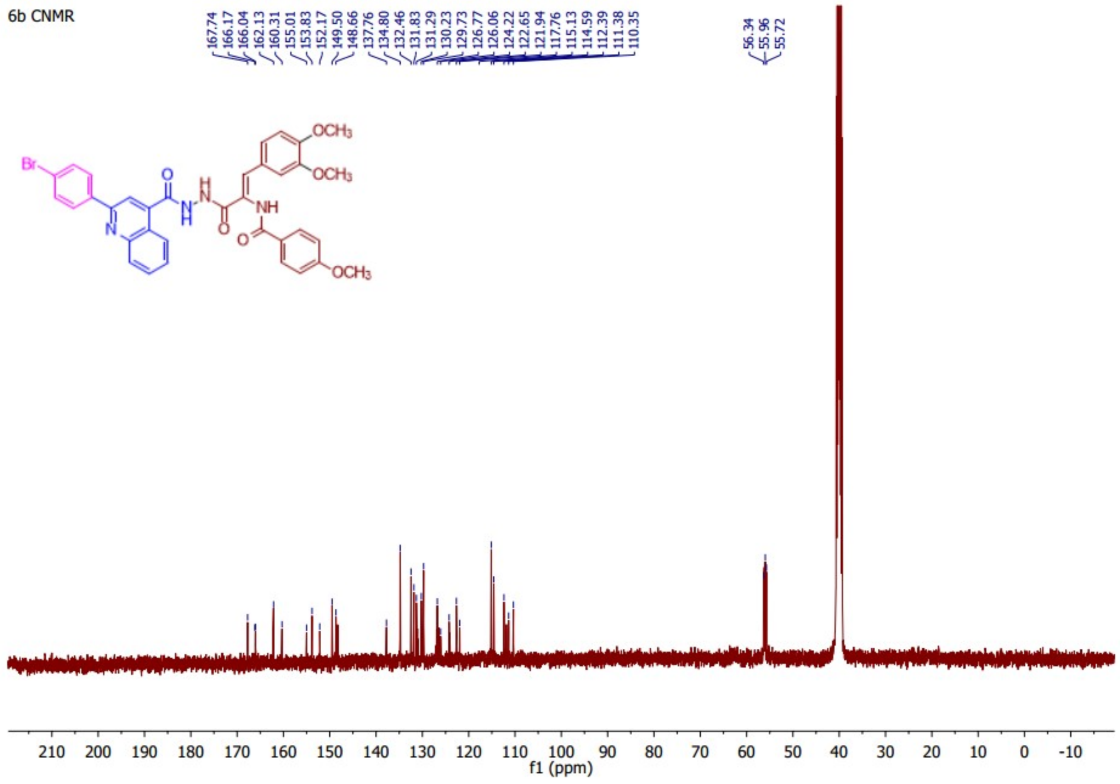


**Figure S17:** $^{13}$C-NMR spectrum of compound **6b**

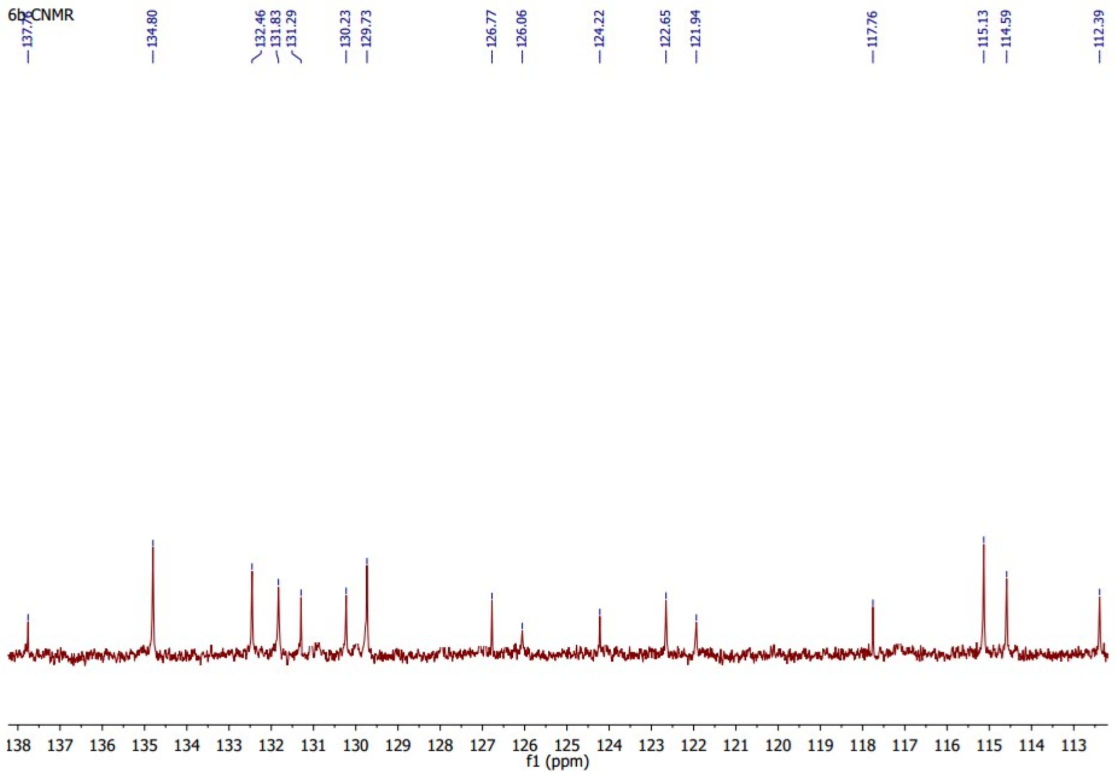


**Figure S18:** $^{13}$C-NMR spectrum of compound **6b (**zoom-in window**)**

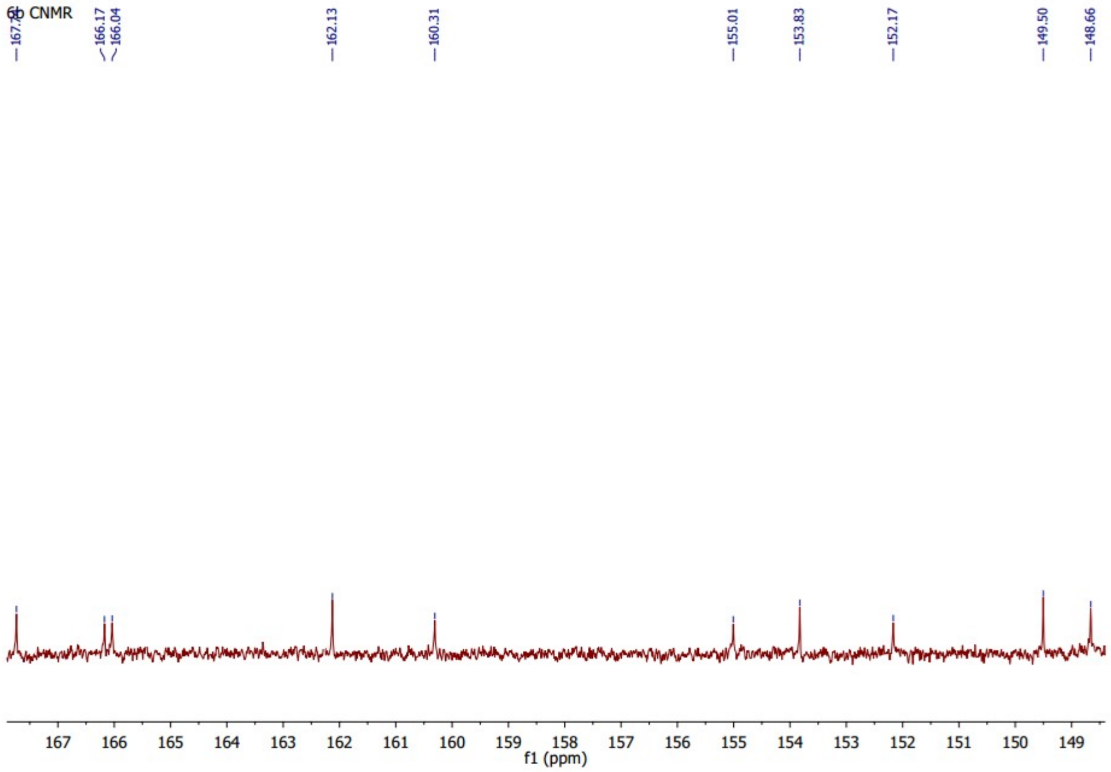


**Figure S19:** $^{13}$C-NMR spectrum of compound **6b (**zoom-in window**)**

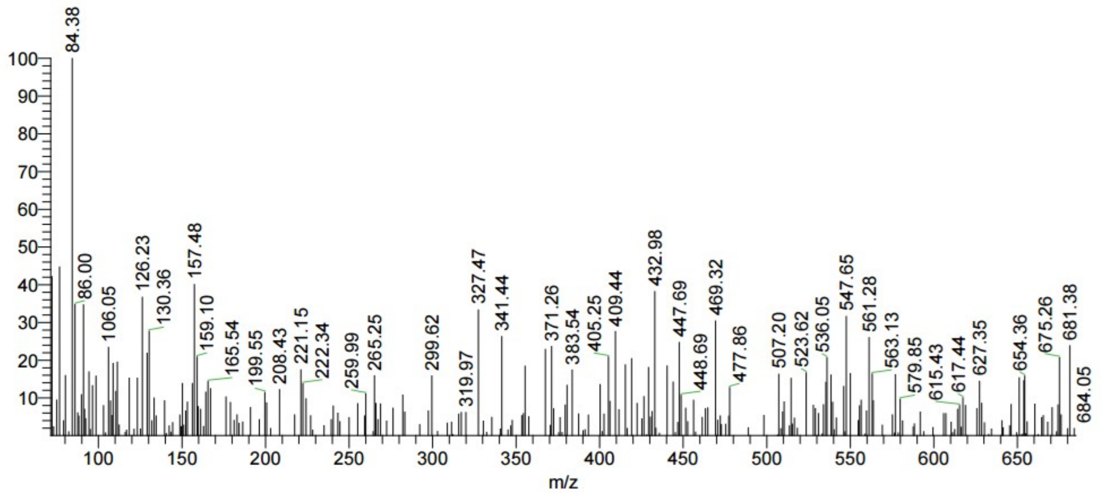


**Figure S20:** Mass spectrum of compound **6b**

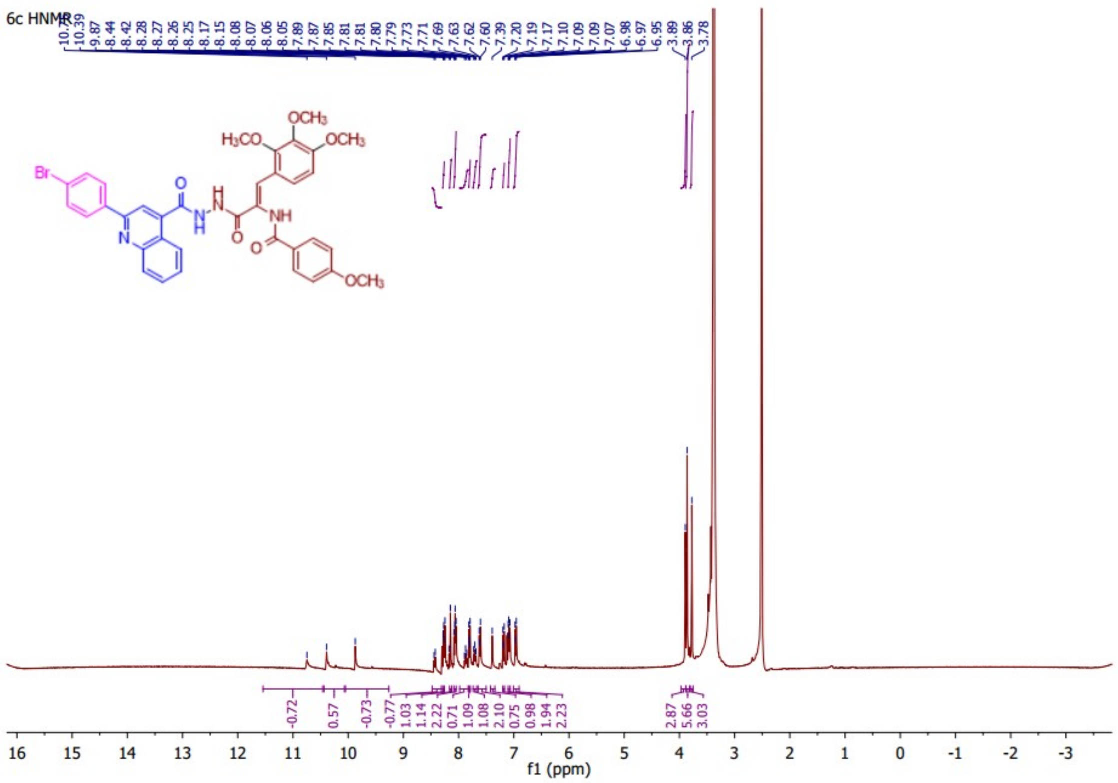

**Figure S21:** $^{1}$H-NMR spectrum of compound **6c**

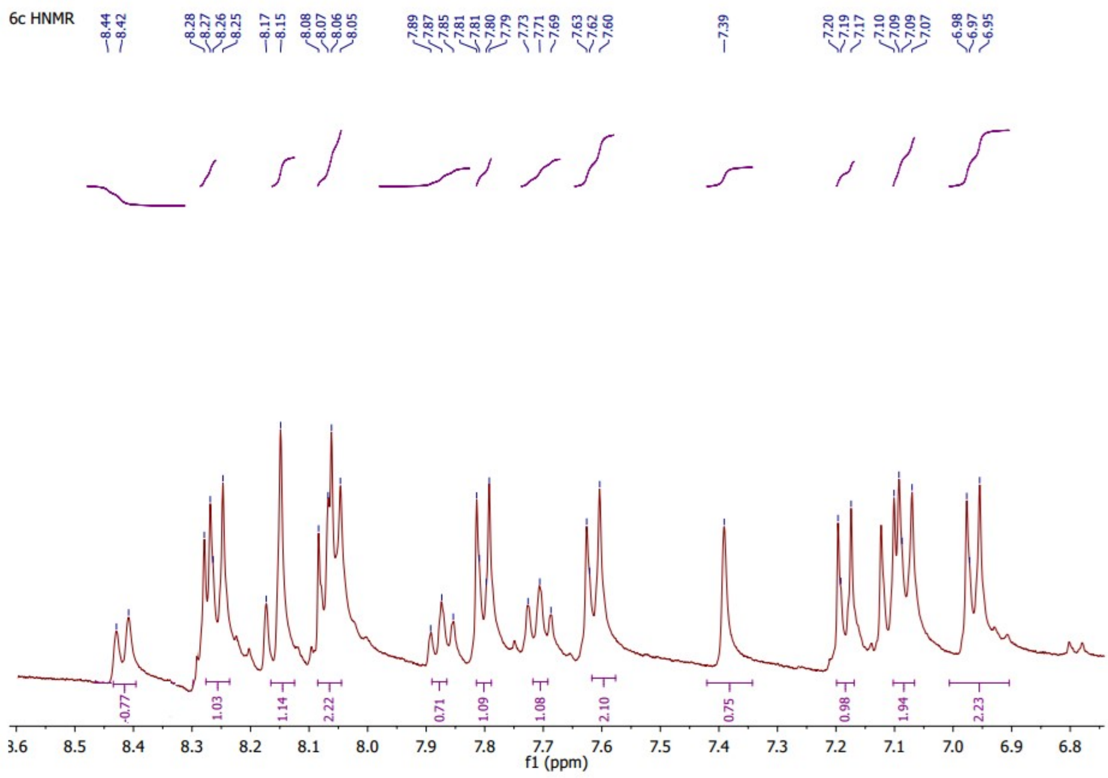


**Figure S22:** $^{1}$H-NMR spectrum of compound **6c (**zoom-in window**)**

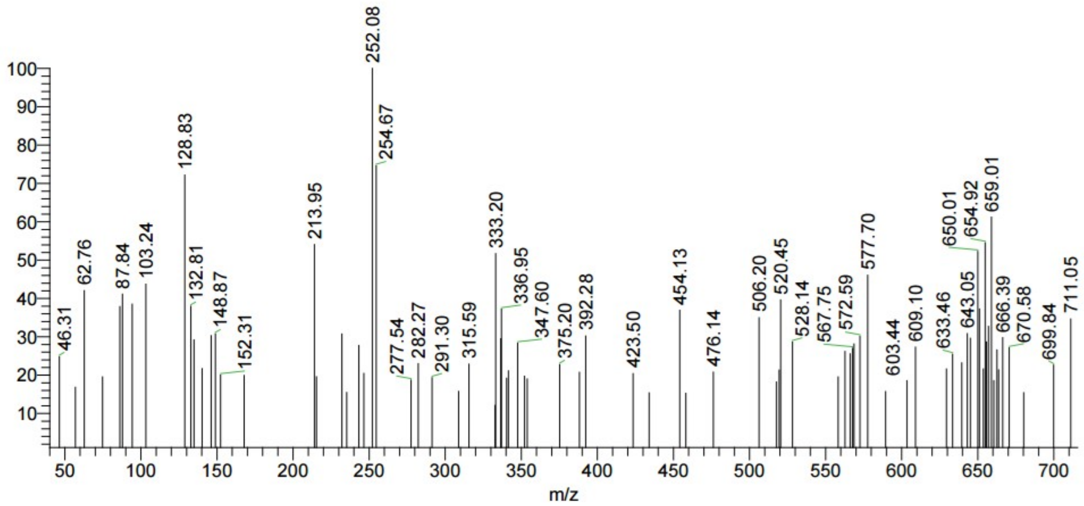


**Figure S23:** Mass spectrum of compound **6c**

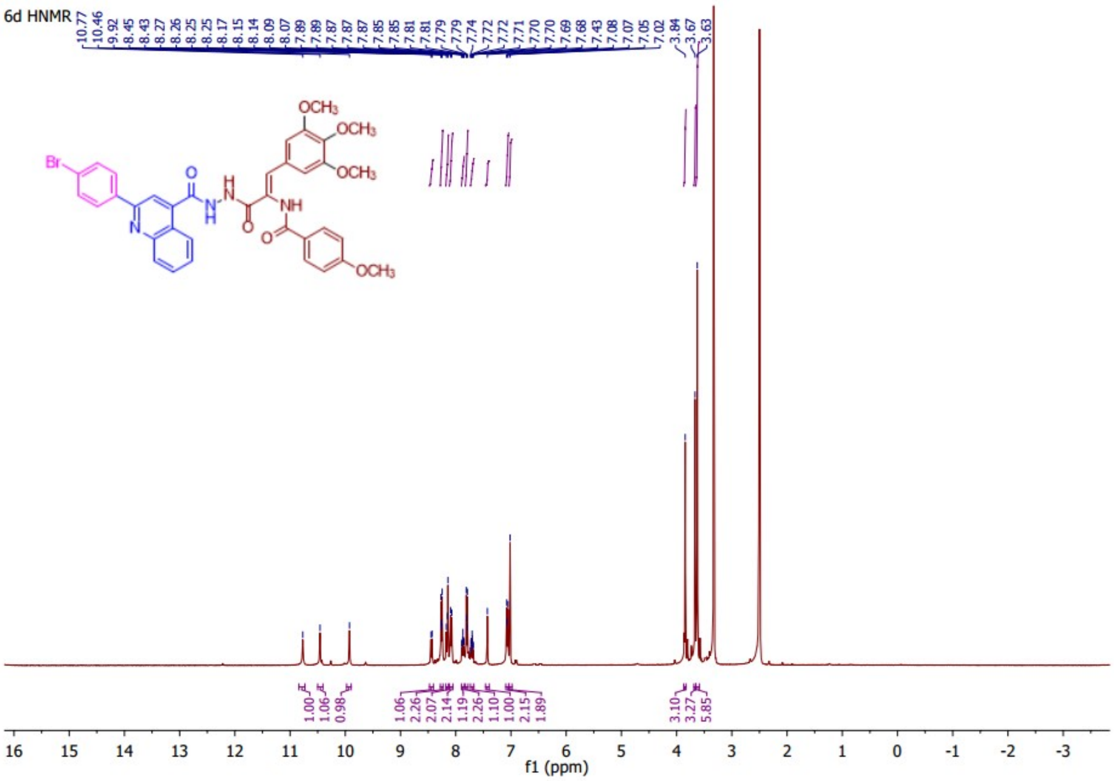


**Figure S24:** $^{1}$H-NMR spectrum of compound **6d**

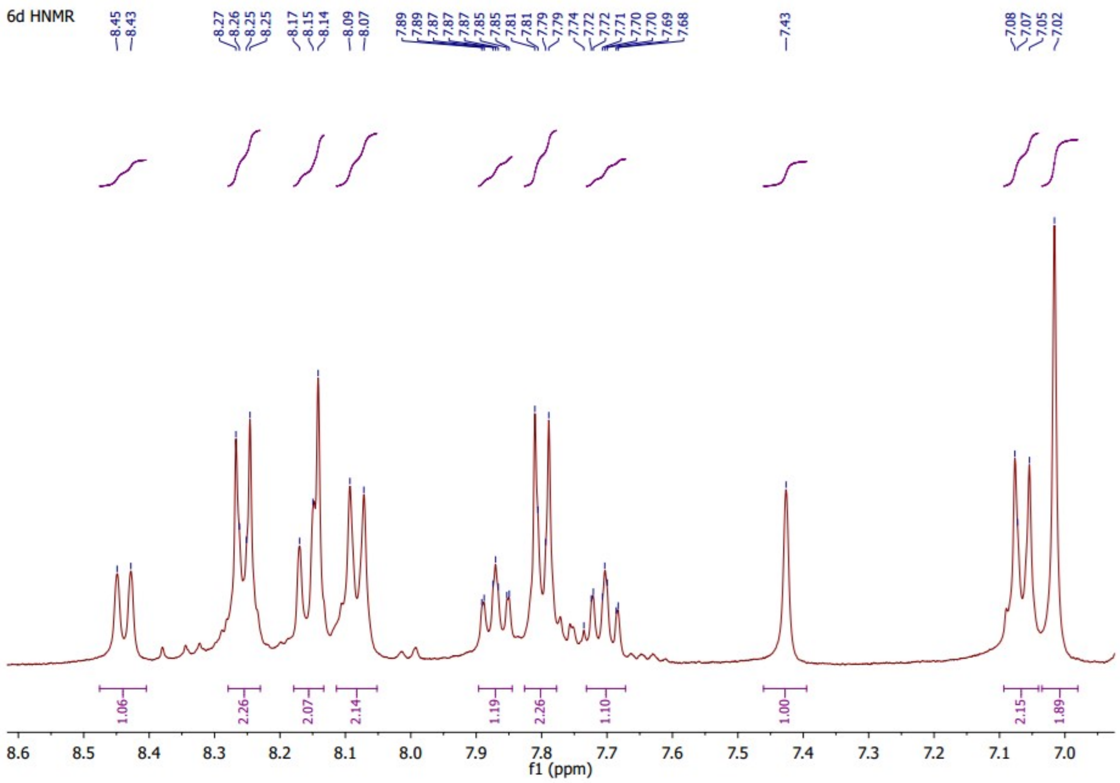


**Figure S25:** $^{1}$H-NMR spectrum of compound **6d (**zoom-in window**)**

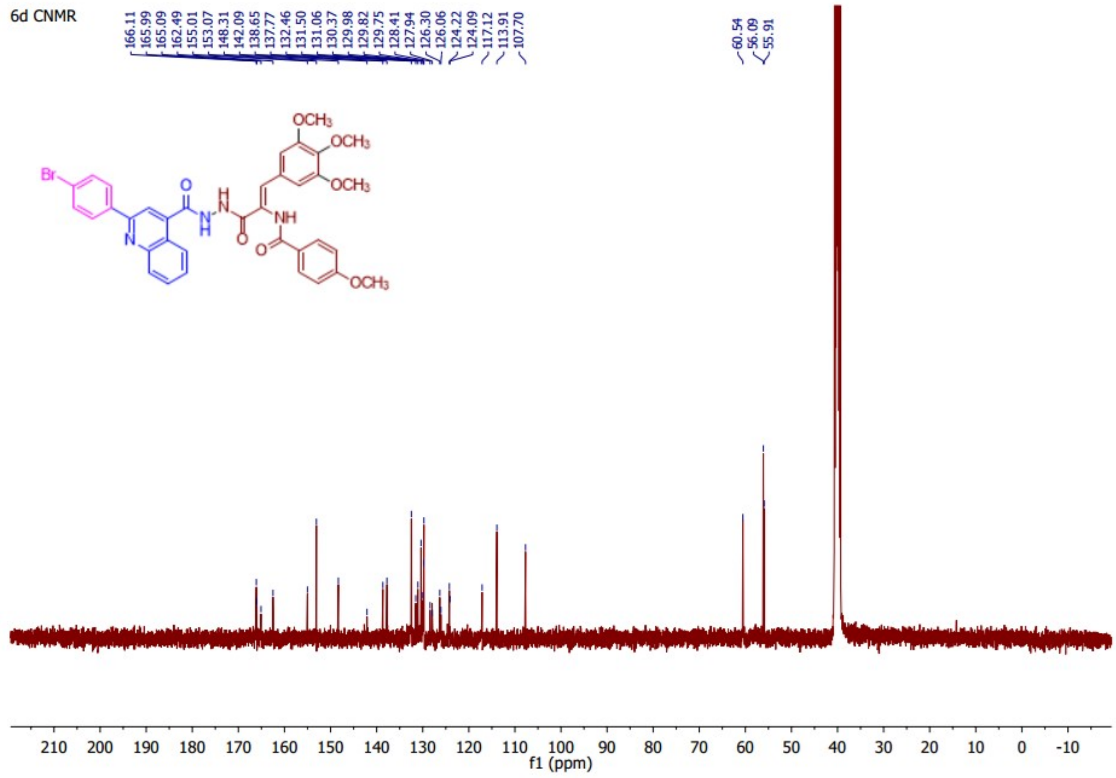


**Figure S26:** $^{13}$C-NMR spectrum of compound **6d**

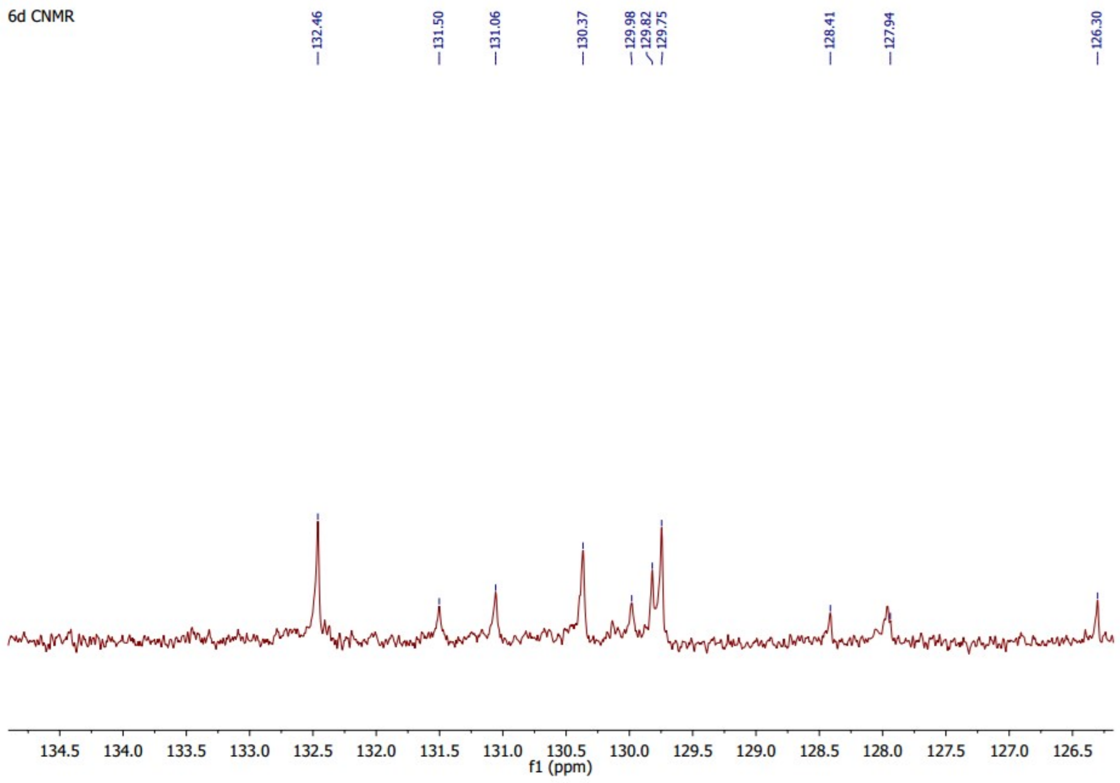


**Figure S27:** $^{13}$C-NMR spectrum of compound **6d** (zoom-in window)

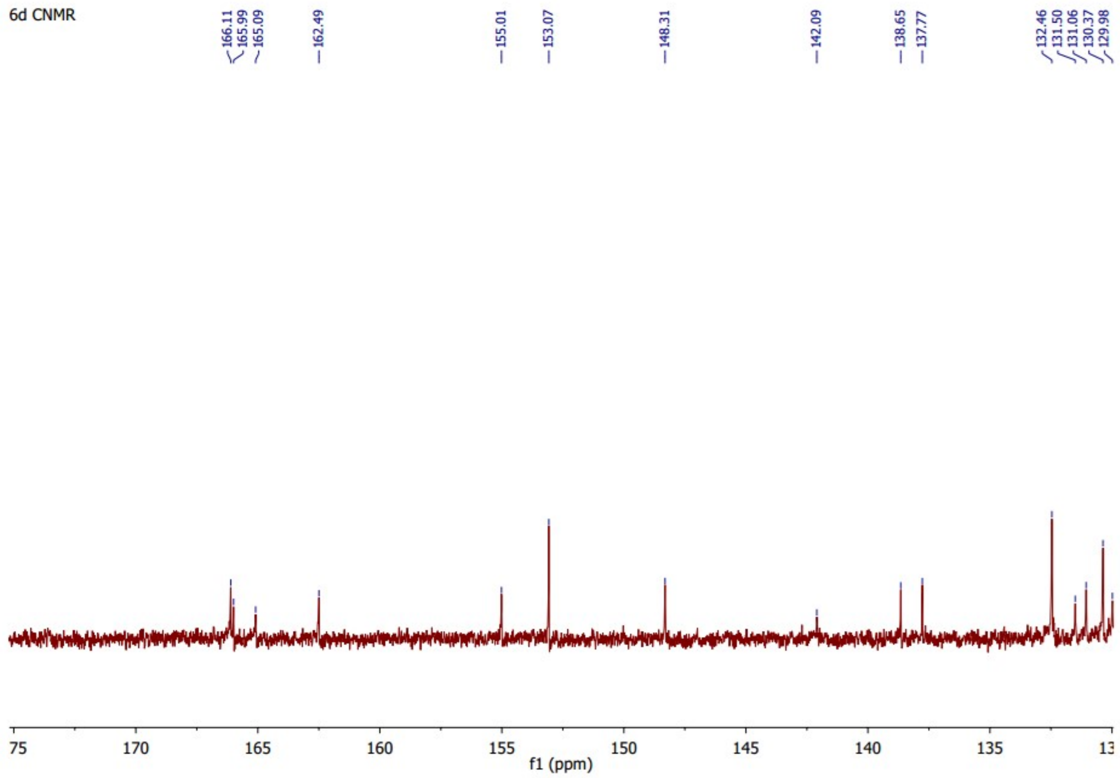


**Figure S28:** $^{13}$C-NMR spectrum of compound **6d (**zoom-in window**)**

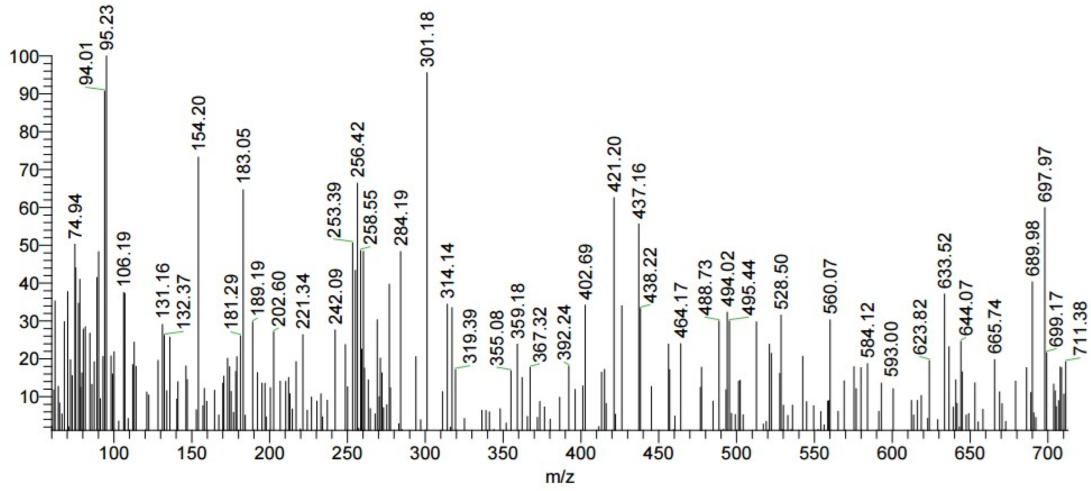


**Figure S29:** Mass spectrum of compound **6d**

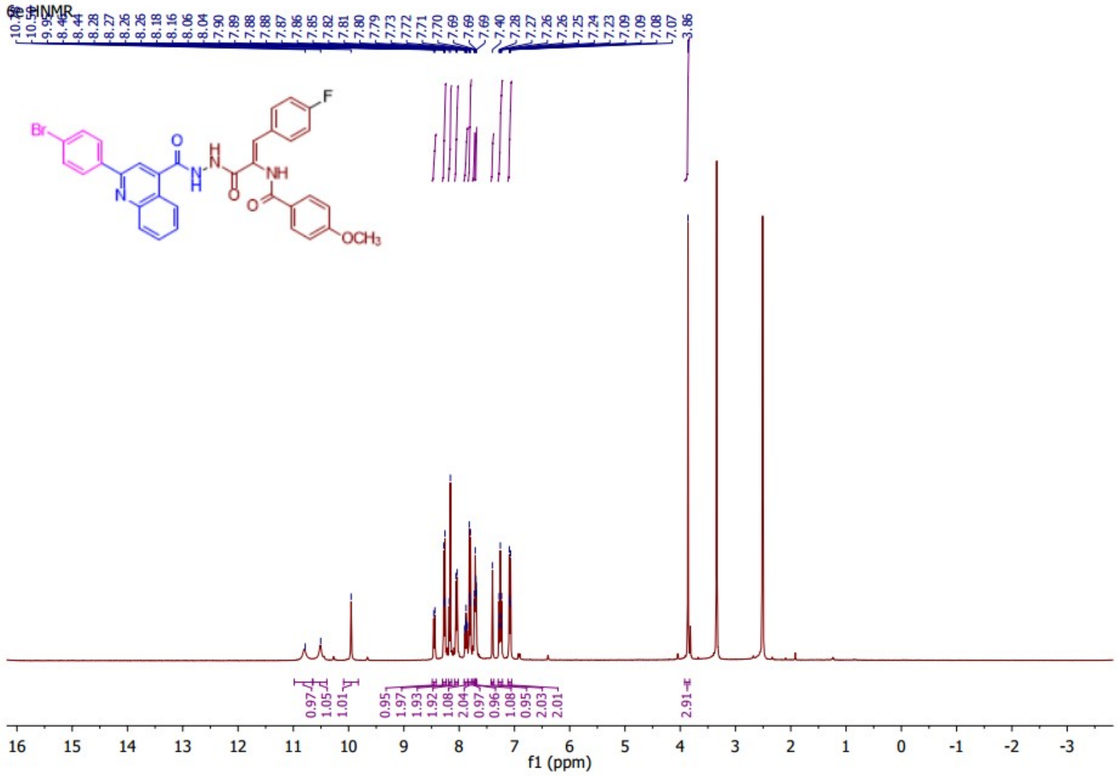


**Figure S30:** $^{1}$H-NMR spectrum of compound **6e**

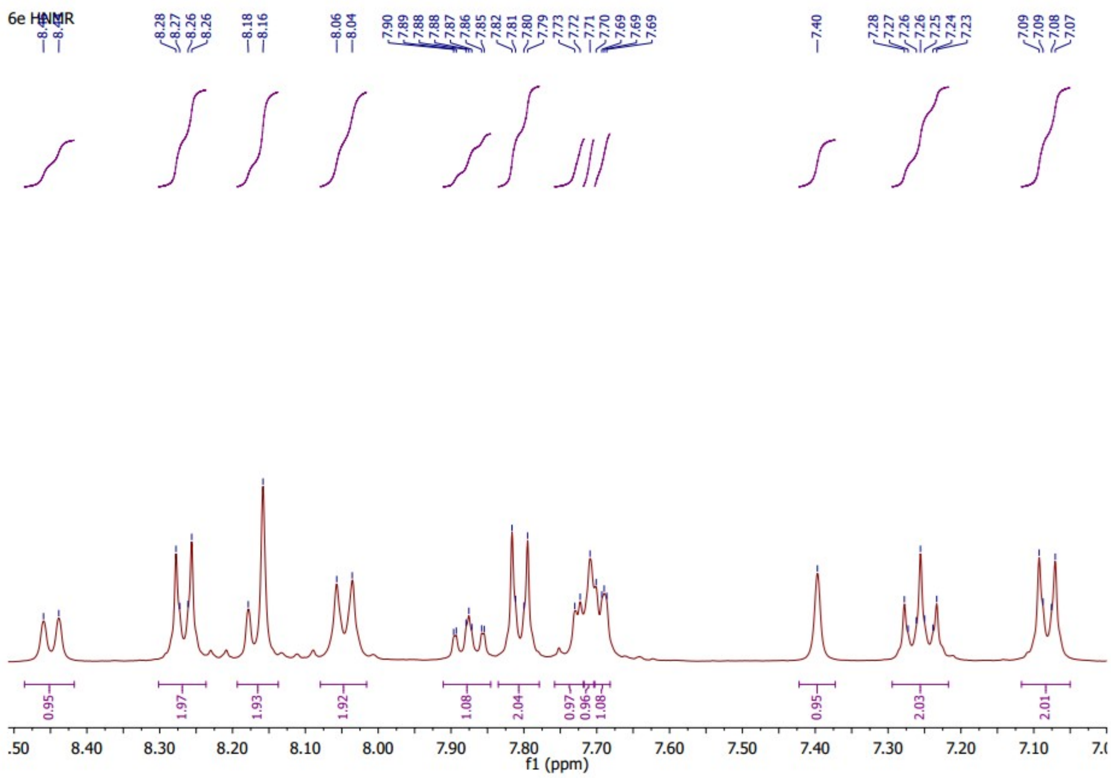

**Figure S31:** $^{1}$H-NMR spectrum of compound **6e** (zoom-in window)

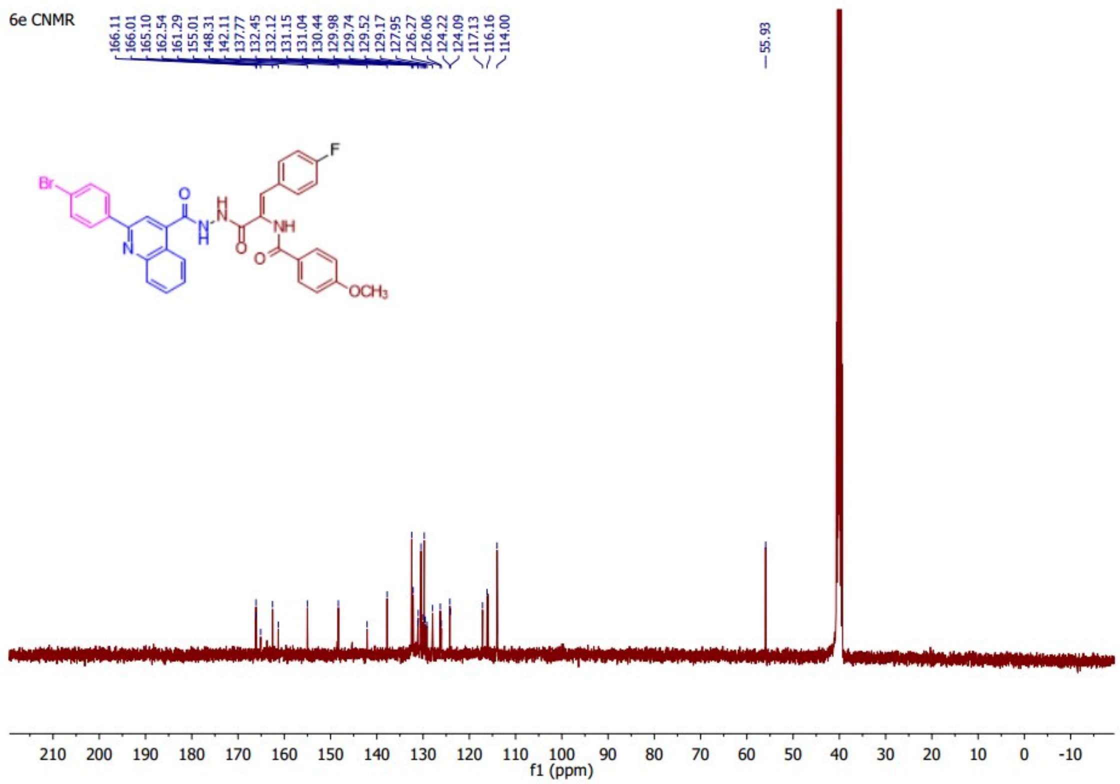


**Figure S32:** $^{13}$C-NMR spectrum of compound **6e**

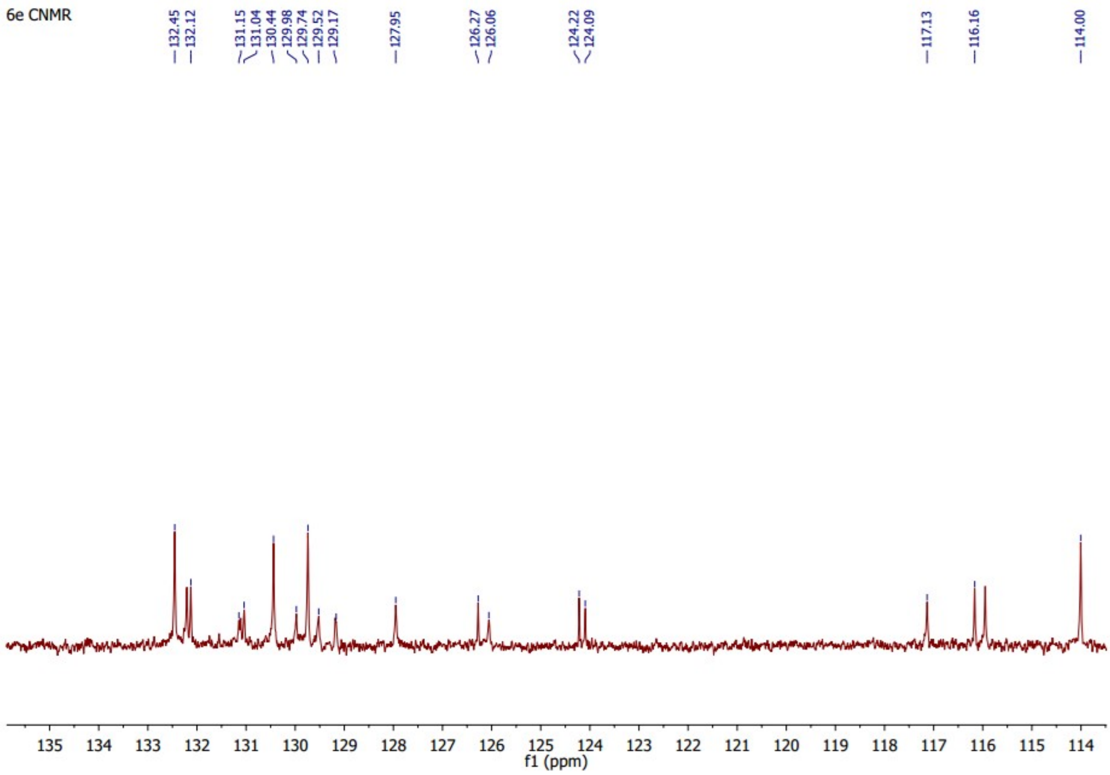


**Figure S33:** $^{13}$C-NMR spectrum of compound **6e (**zoom-in window**)**

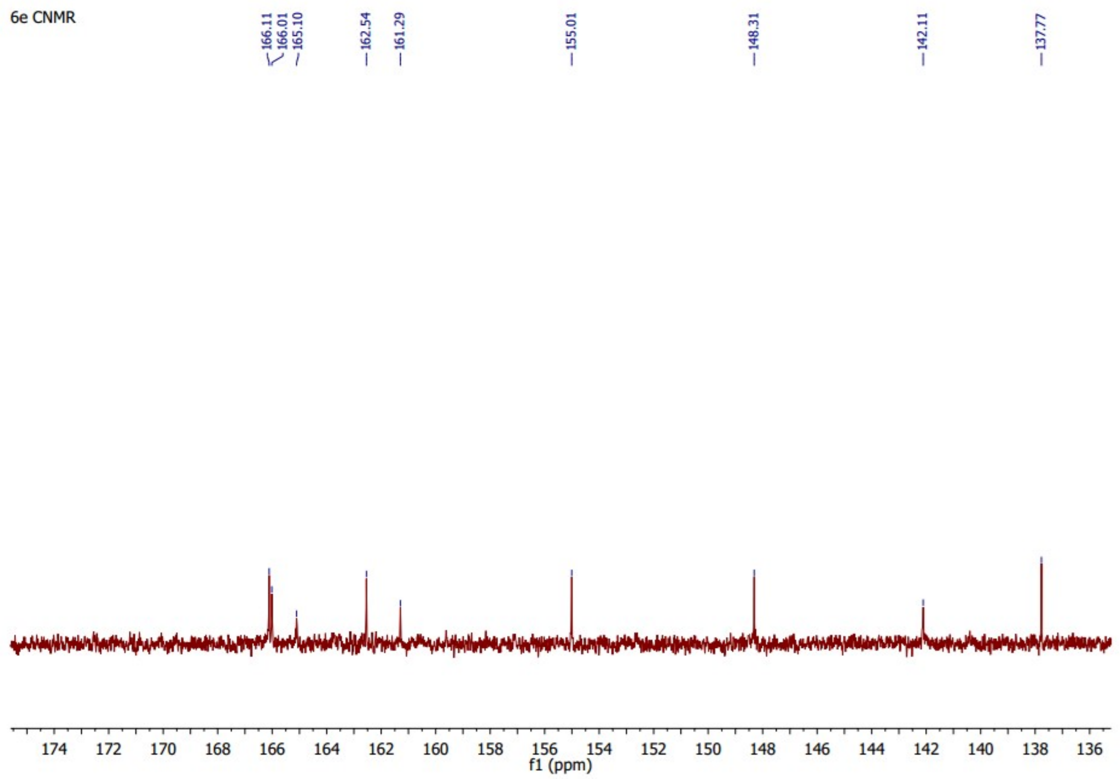


**Figure S34:** $^{13}$C-NMR spectrum of compound **6e (**zoom-in window**)**

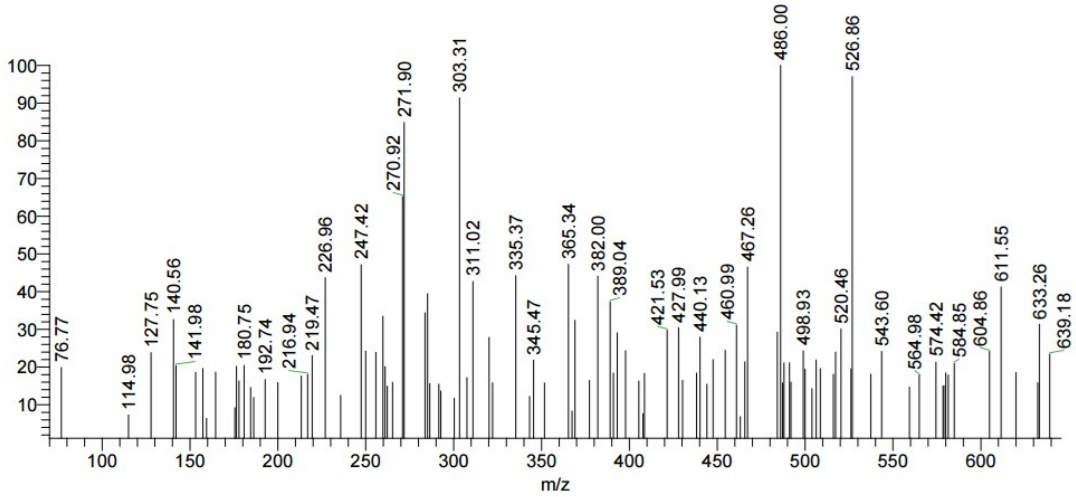


**Figure S35:** Mass spectrum of compound **6e**

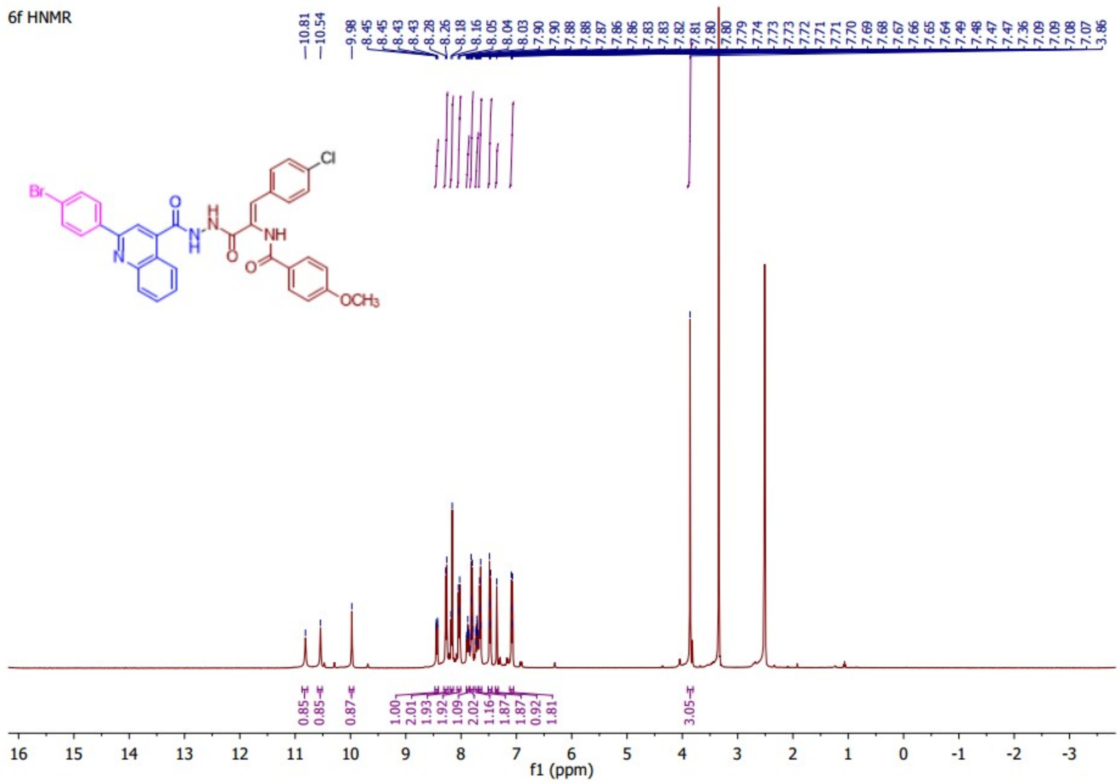


**Figure S36:** $^{1}$H-NMR spectrum of compound **6f**

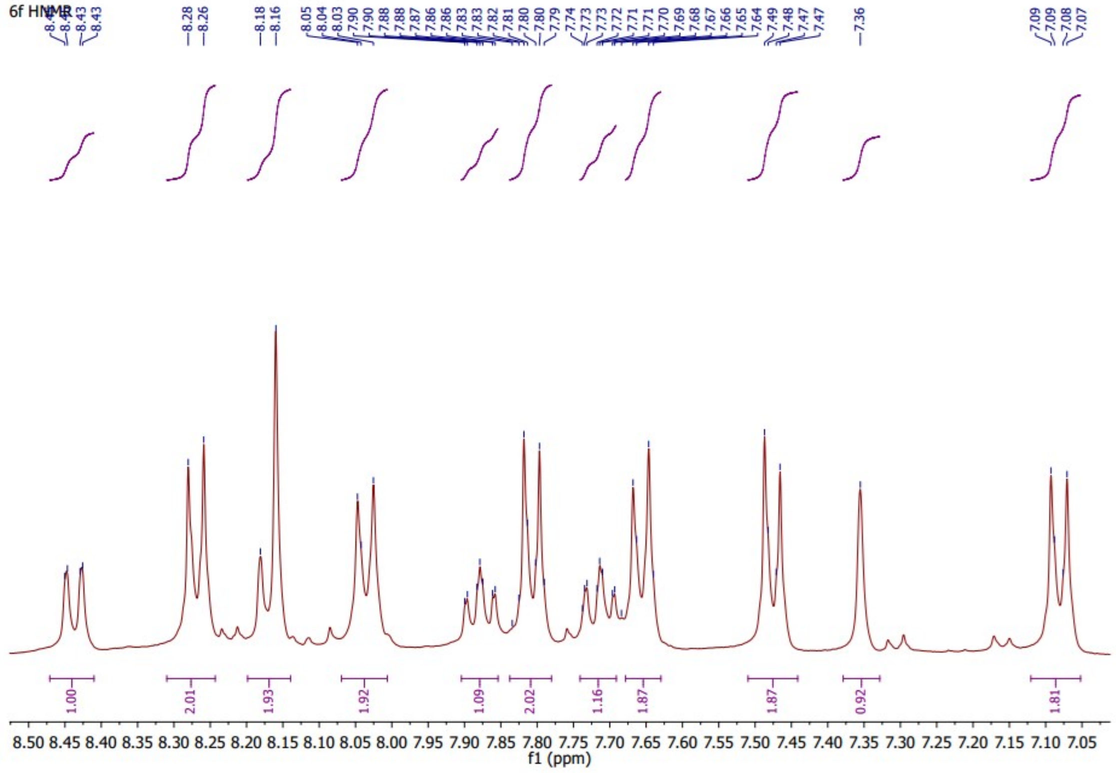

**Figure S37:** $^{1}$H-NMR spectrum of compound **6f** (zoom-in window)

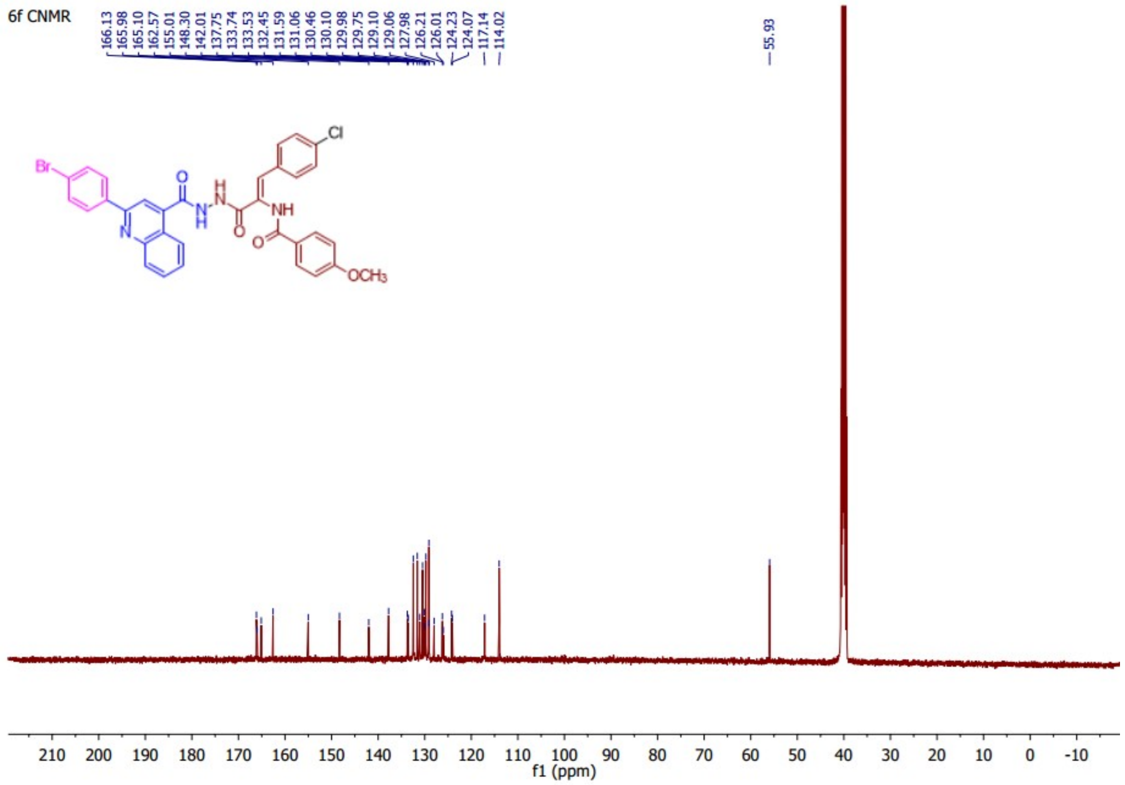


**Figure S38:** $^{13}$C-NMR spectrum of compound **6f**

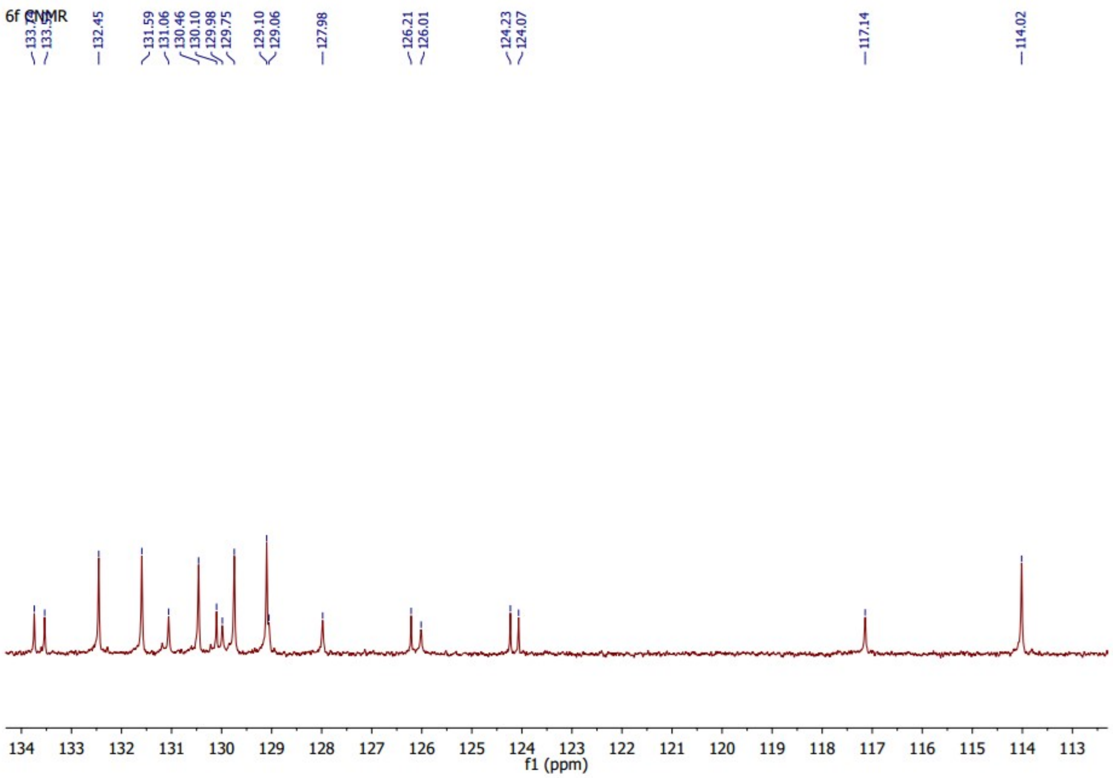

**Figure S39:** $^{13}$C-NMR spectrum of compound **6f (**zoom-in window**)**

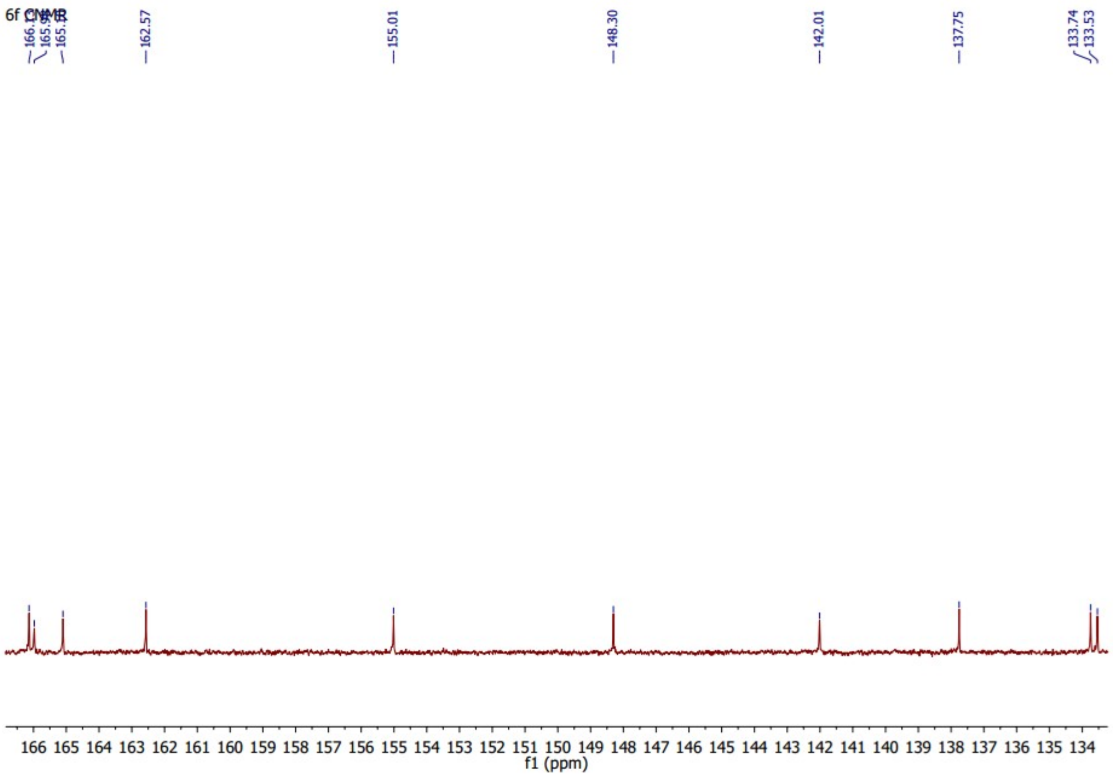

**Figure S40:** $^{13}$C-NMR spectrum of compound **6f (**zoom-in window**)**

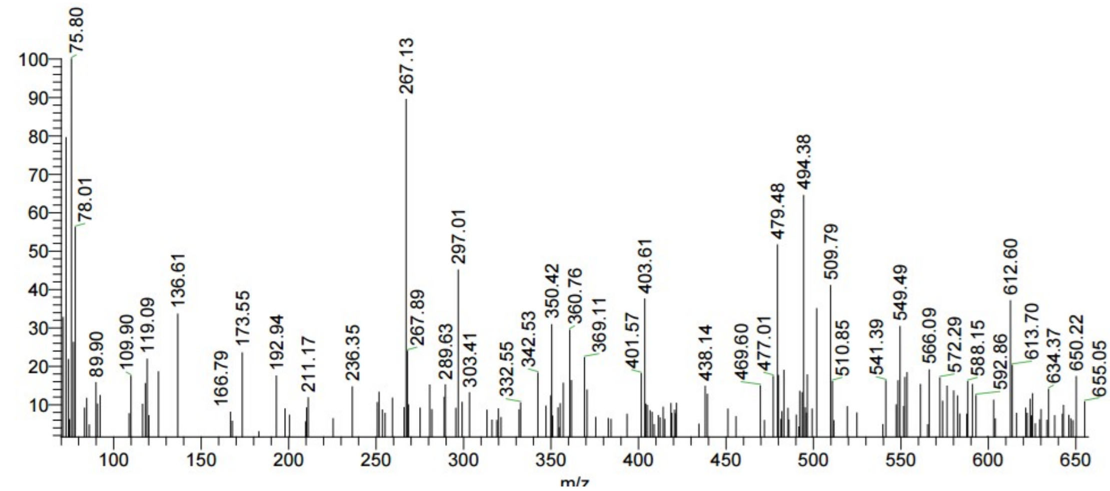


**Figure S41:** Mass spectrum of compound **6f**

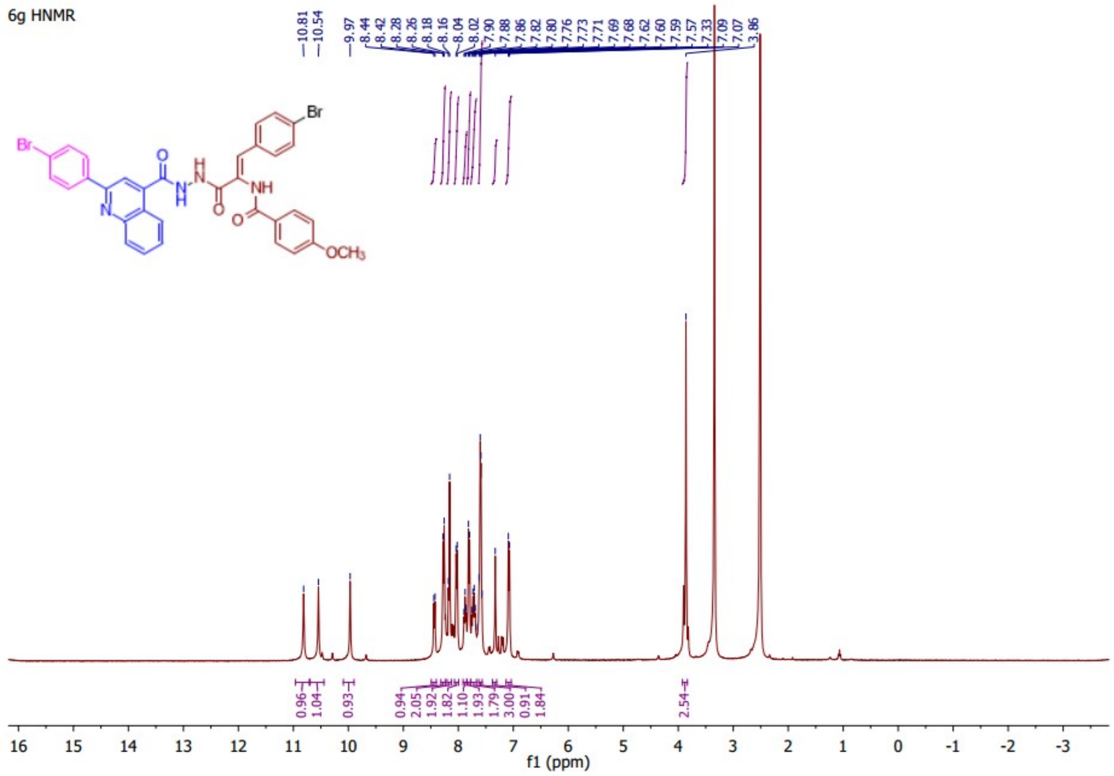

**Figure S42:** $^{1}$H-NMR spectrum of compound **6g**

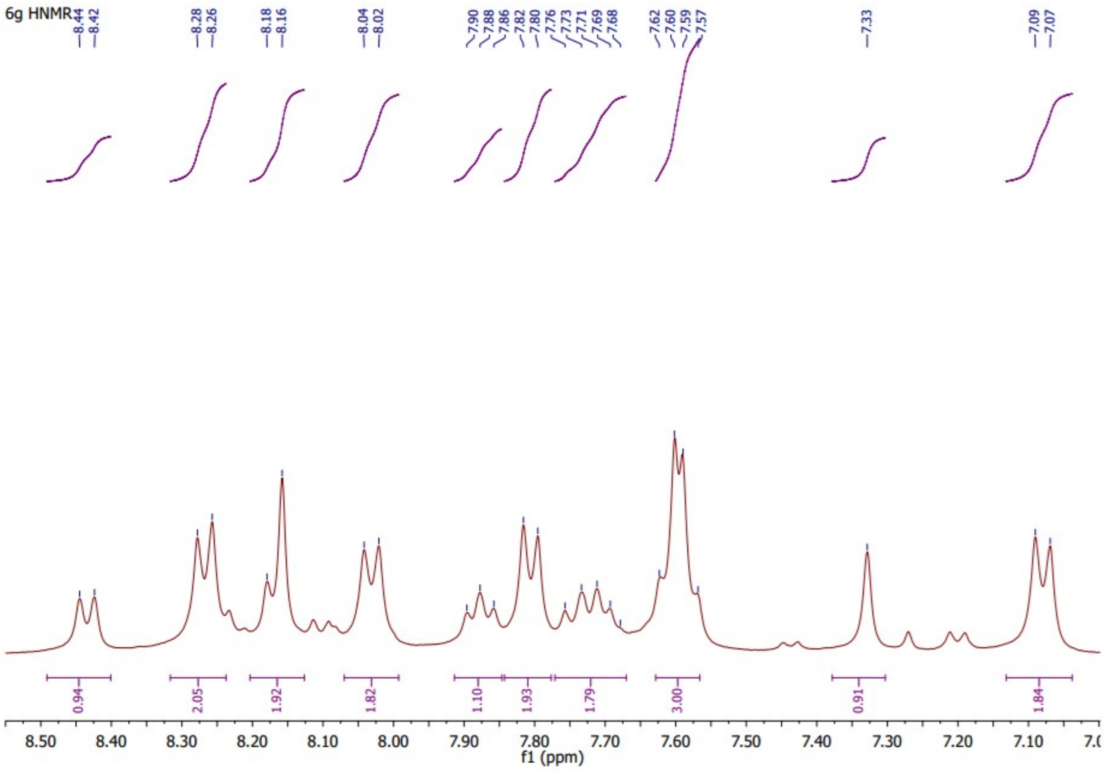


**Figure S43:** $^{1}$H-NMR spectrum of compound **6g (**zoom-in window**)**

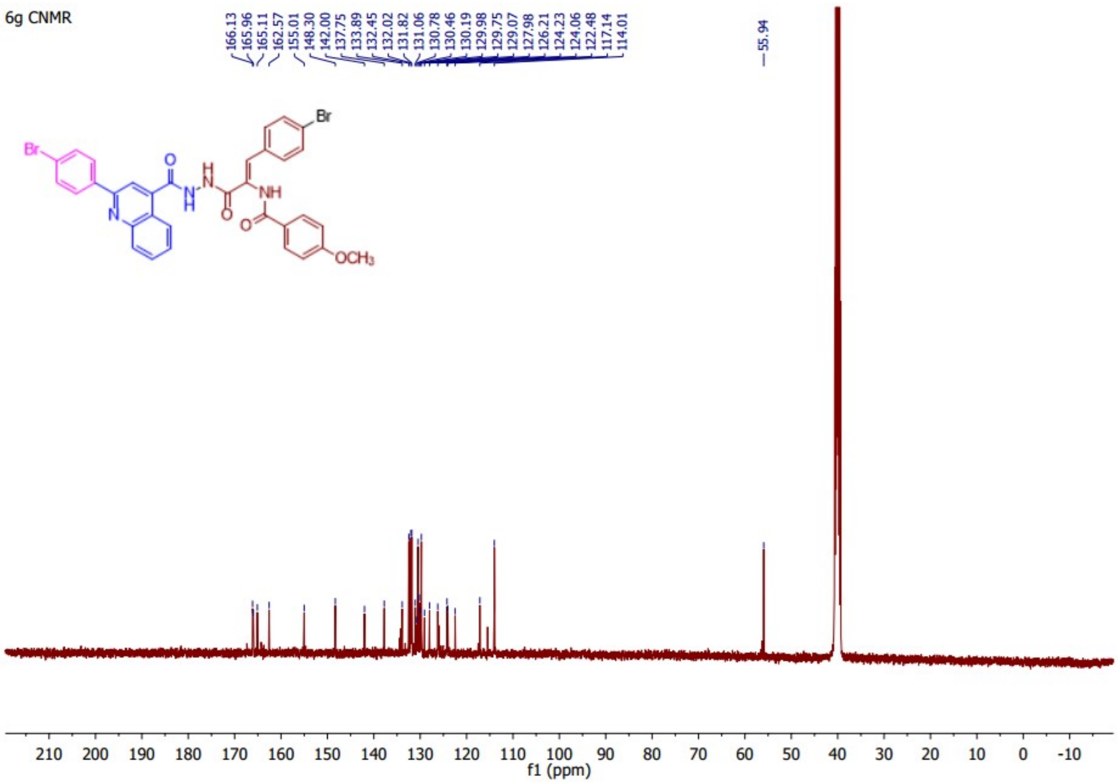


**Figure S44:** $^{13}$C-NMR spectrum of compound **6g**

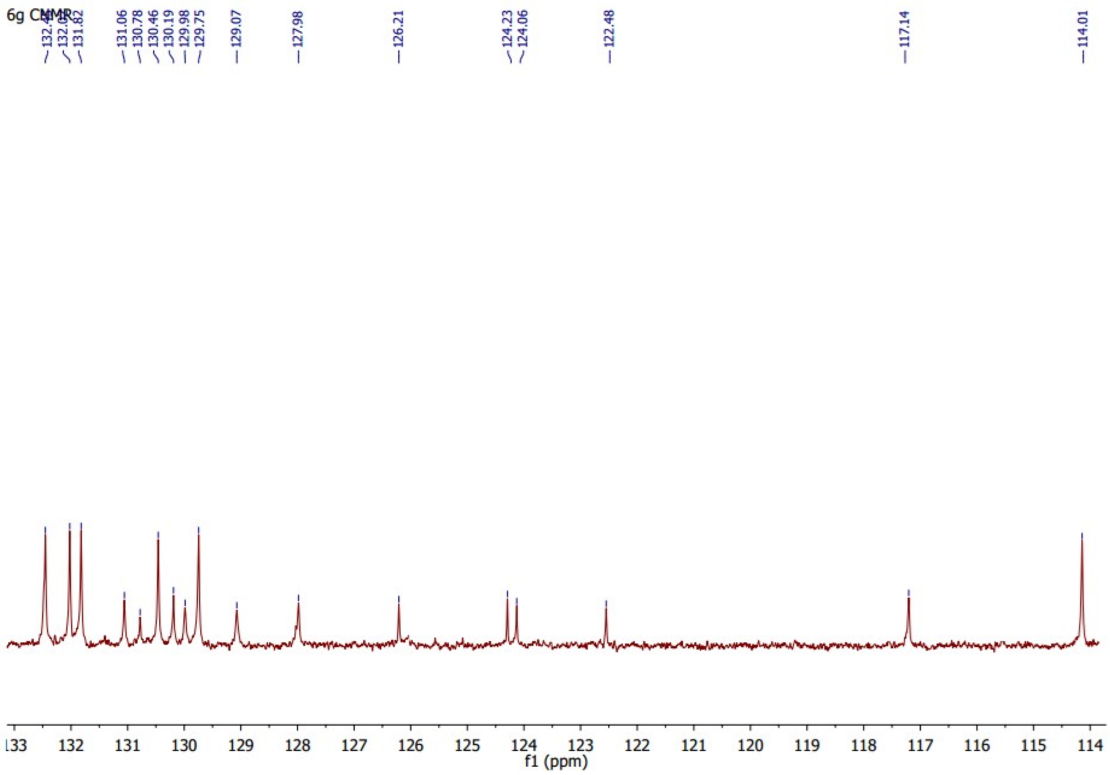


**Figure S45:** $^{13}$C-NMR spectrum of compound **6g (**zoom-in window**)**

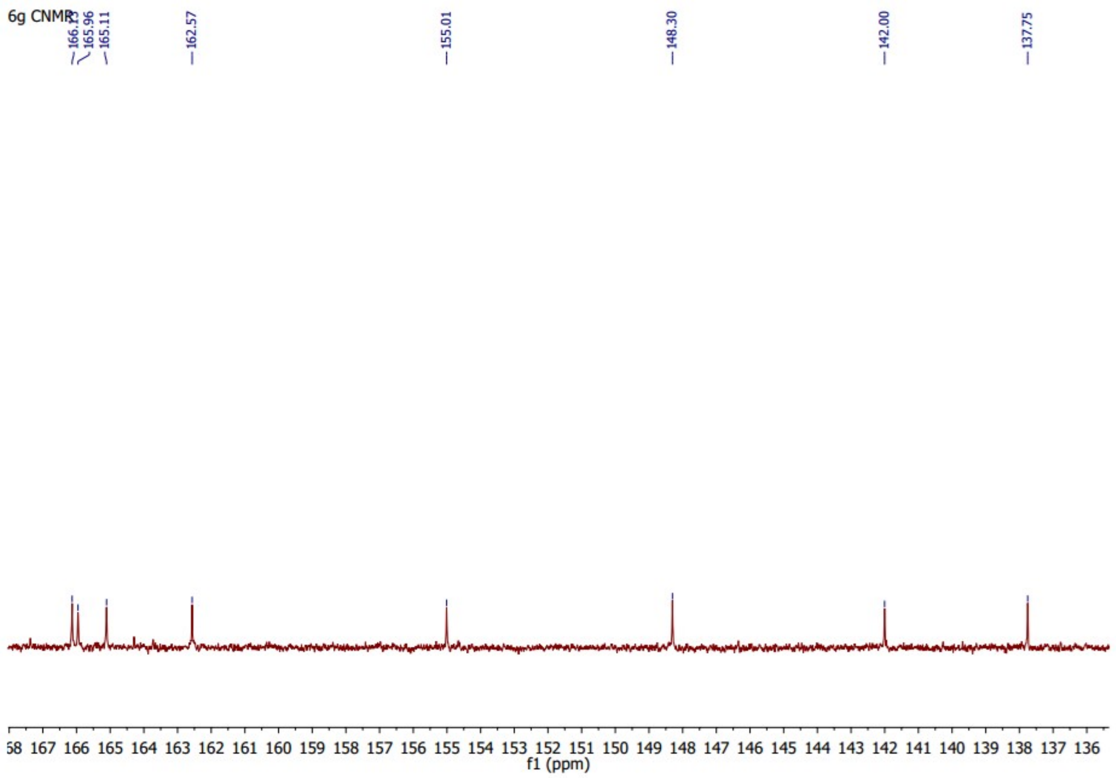


**Figure S46:** $^{13}$C-NMR spectrum of compound **6g (**zoom-in window**)**

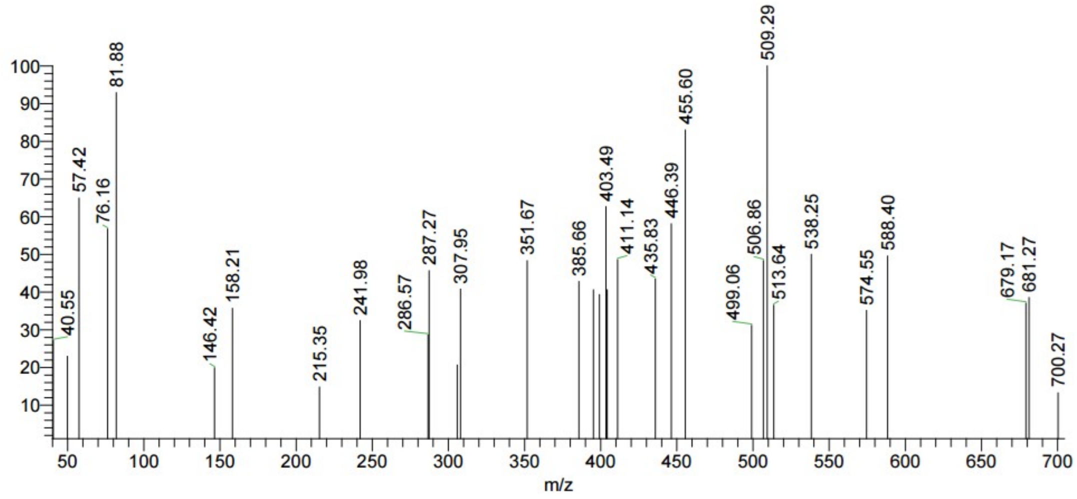


**Figure S47:** Mass spectrum of compound **6g**

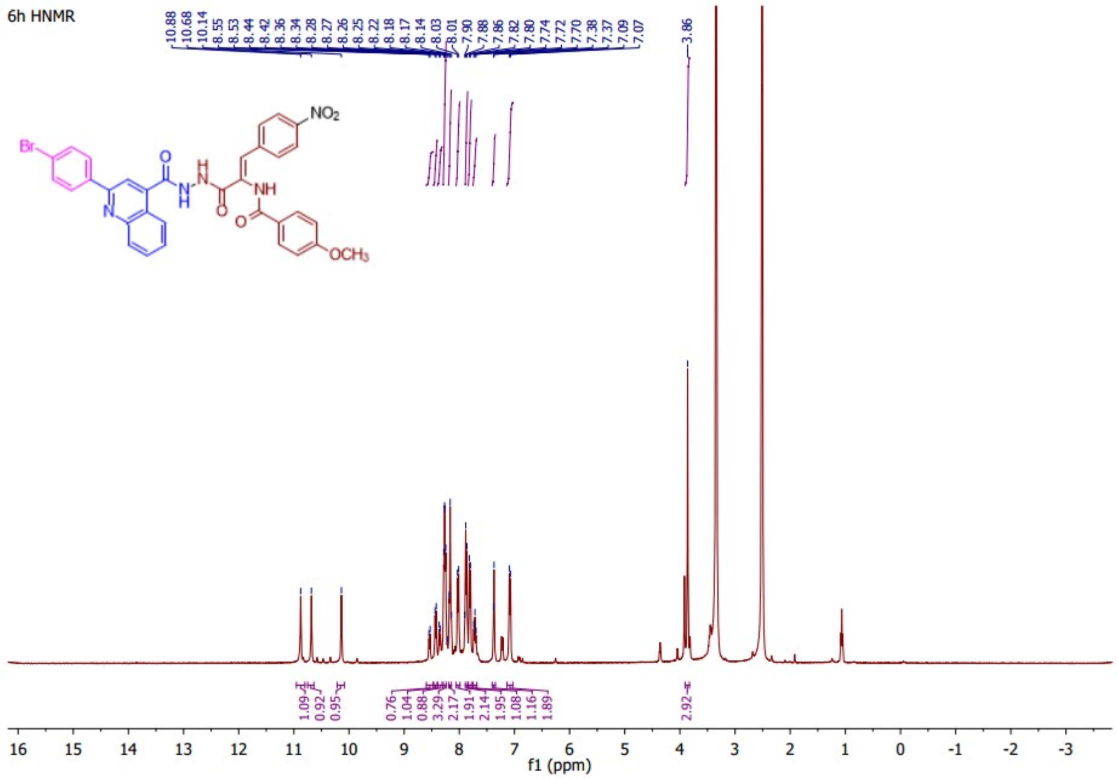


**Figure S48:** $^{1}$H-NMR spectrum of compound **6h**

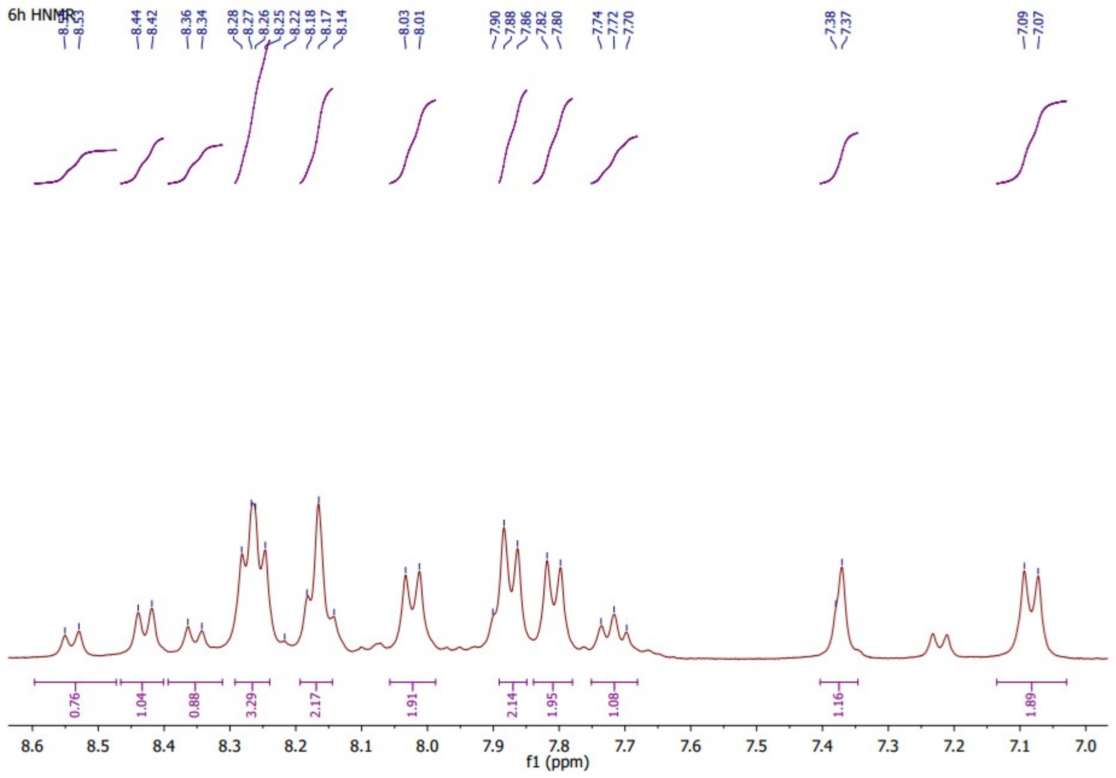

**Figure S49:** $^{1}$H-NMR spectrum of compound **6h** (zoom-in window)

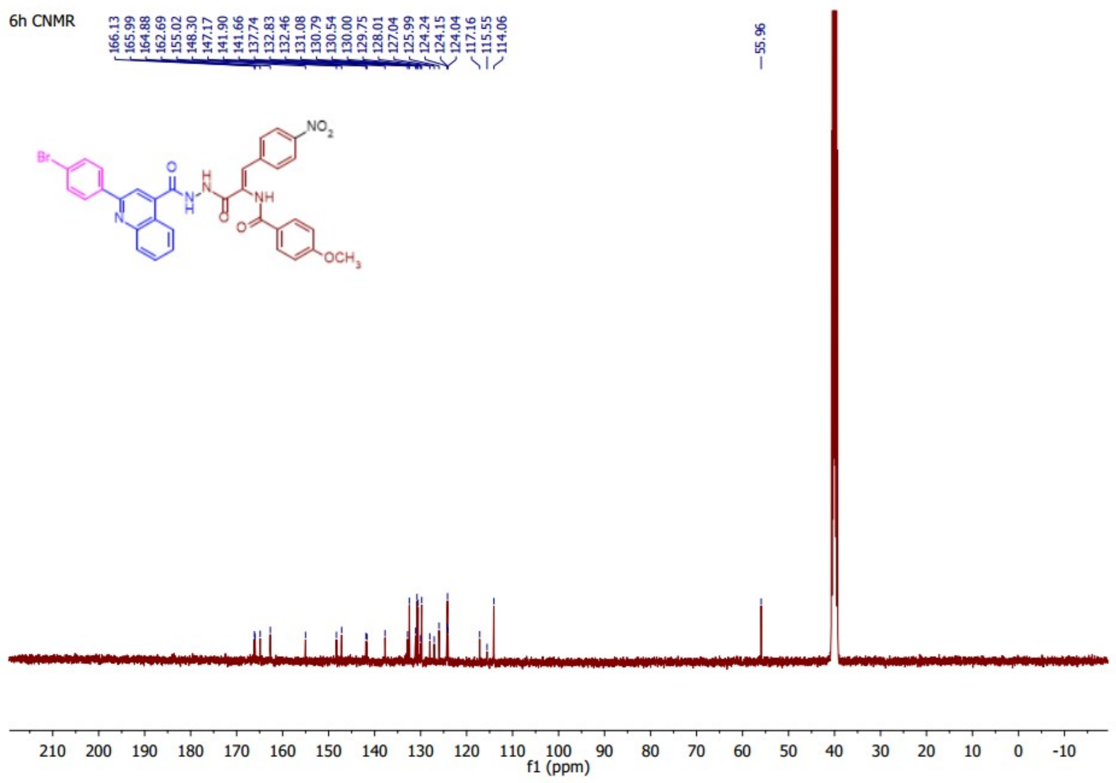


**Figure S50:** $^{13}$C-NMR spectrum of compound **6h**

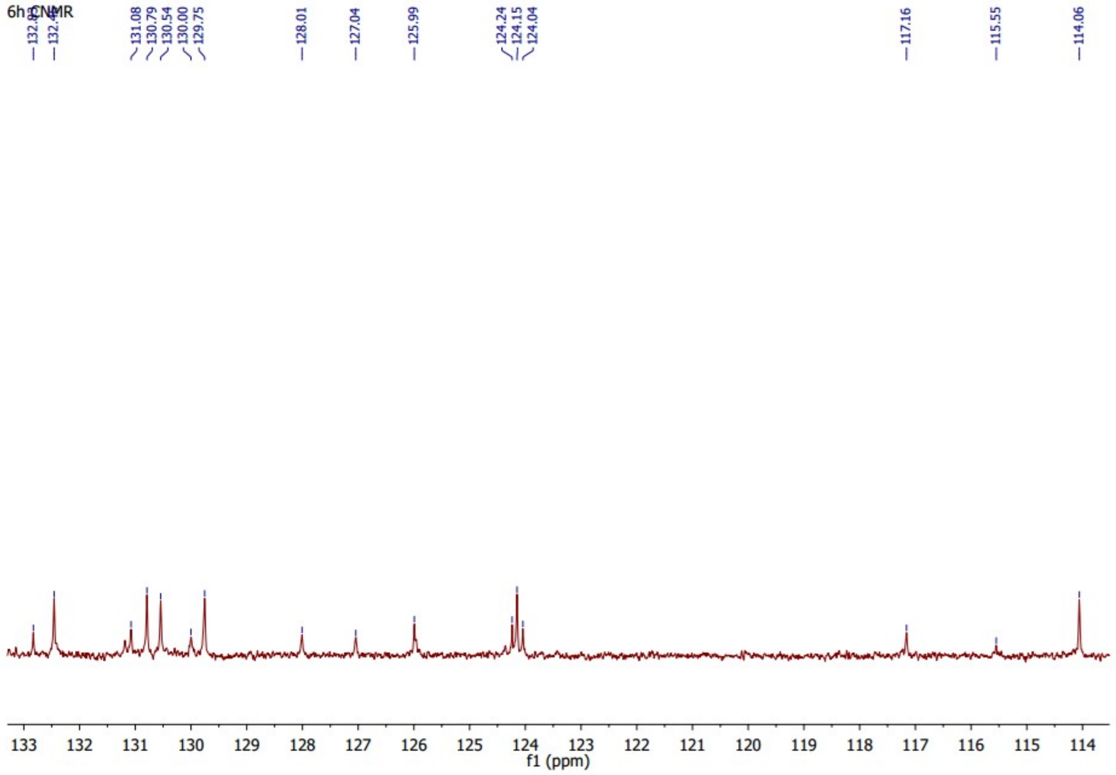


**Figure S51:** $^{13}$C-NMR spectrum of compound **6h(**zoom-in window**)**

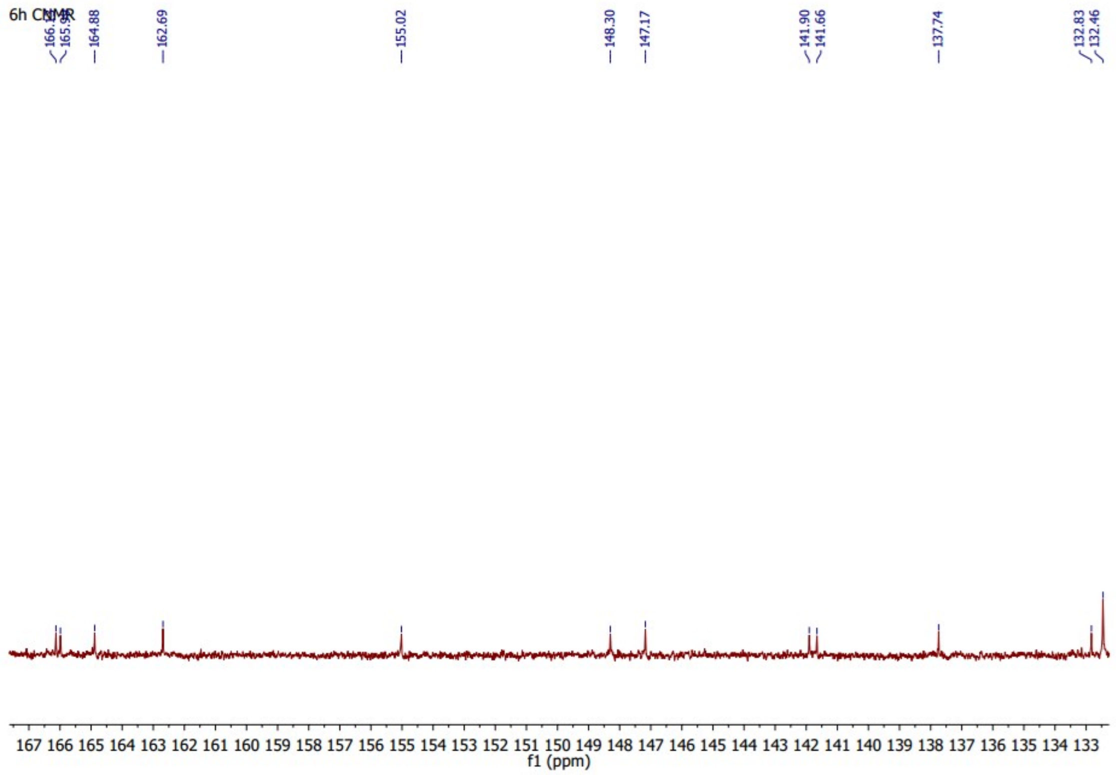


**Figure S52:** $^{13}$C-NMR spectrum of compound **6h (**zoom-in window**)**

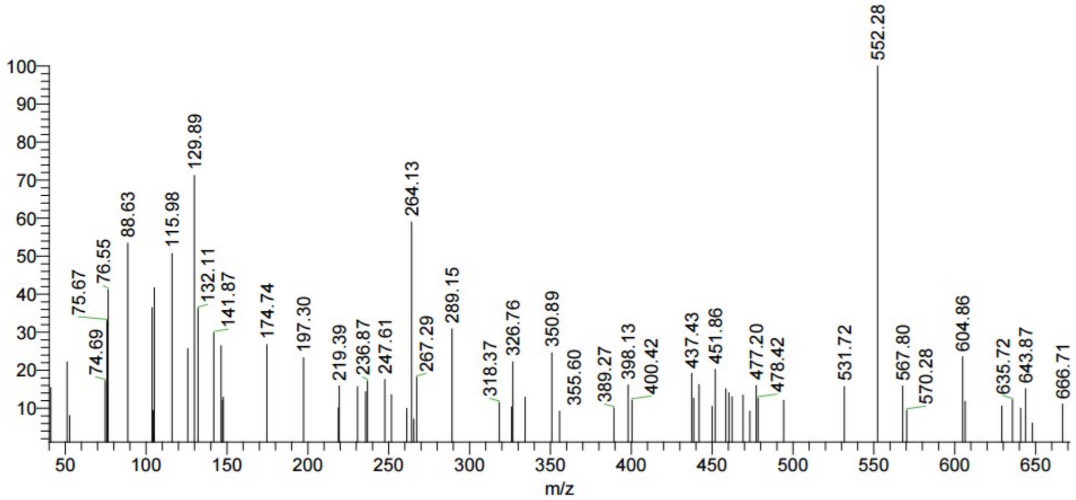


**Figure S53:** Mass spectrum of compound **6h**

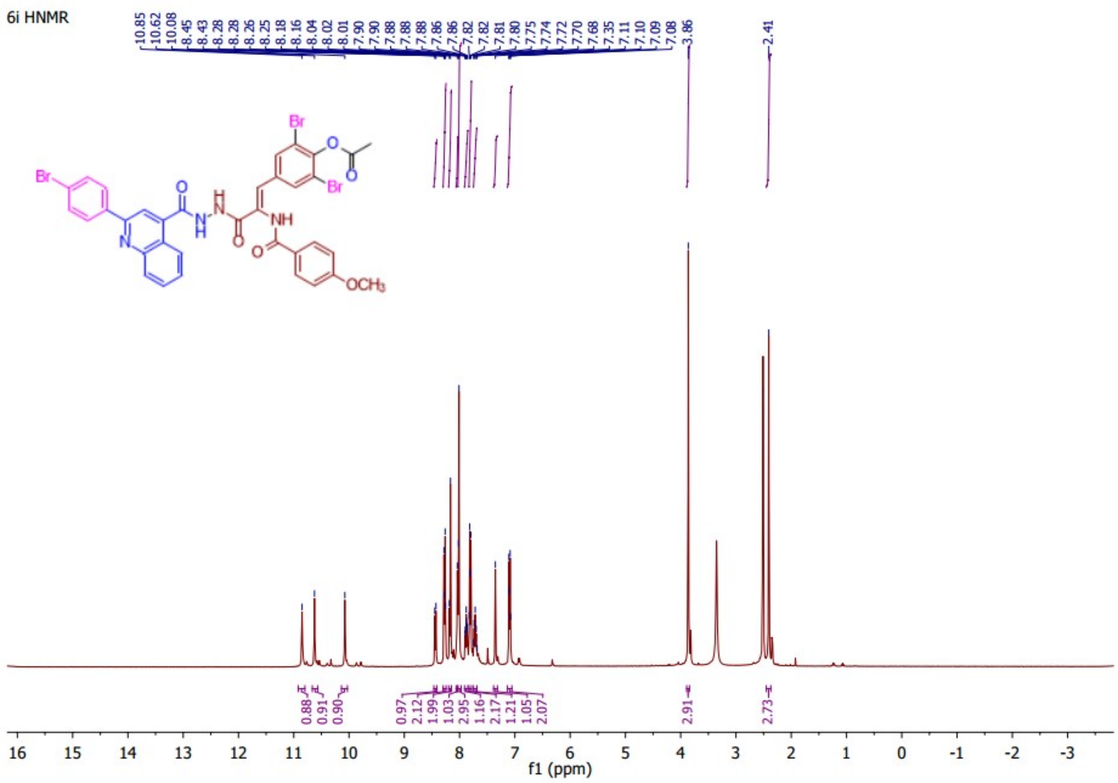

**Figure S54:** $^{1}$H-NMR spectrum of compound **6i**

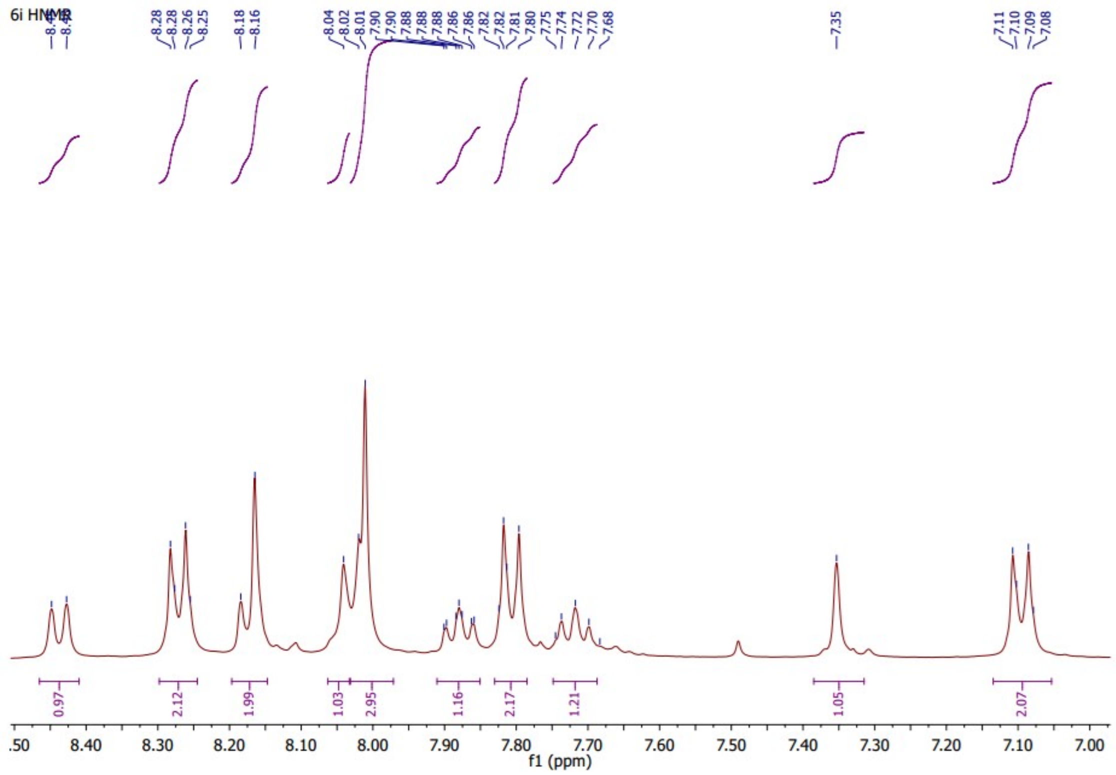

**Figure S55:** $^{1}$H-NMR spectrum of compound **6i (**zoom-in window**)**

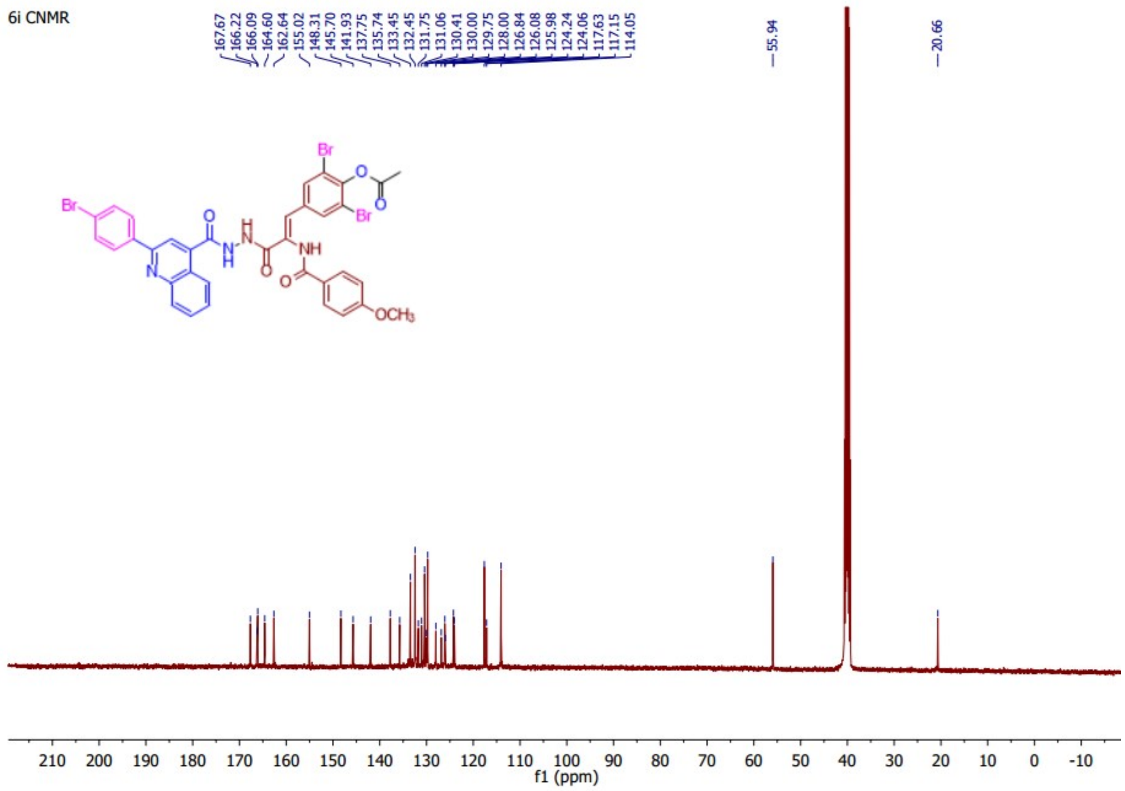


**Figure S56:** $^{13}$C-NMR spectrum of compound **6i**

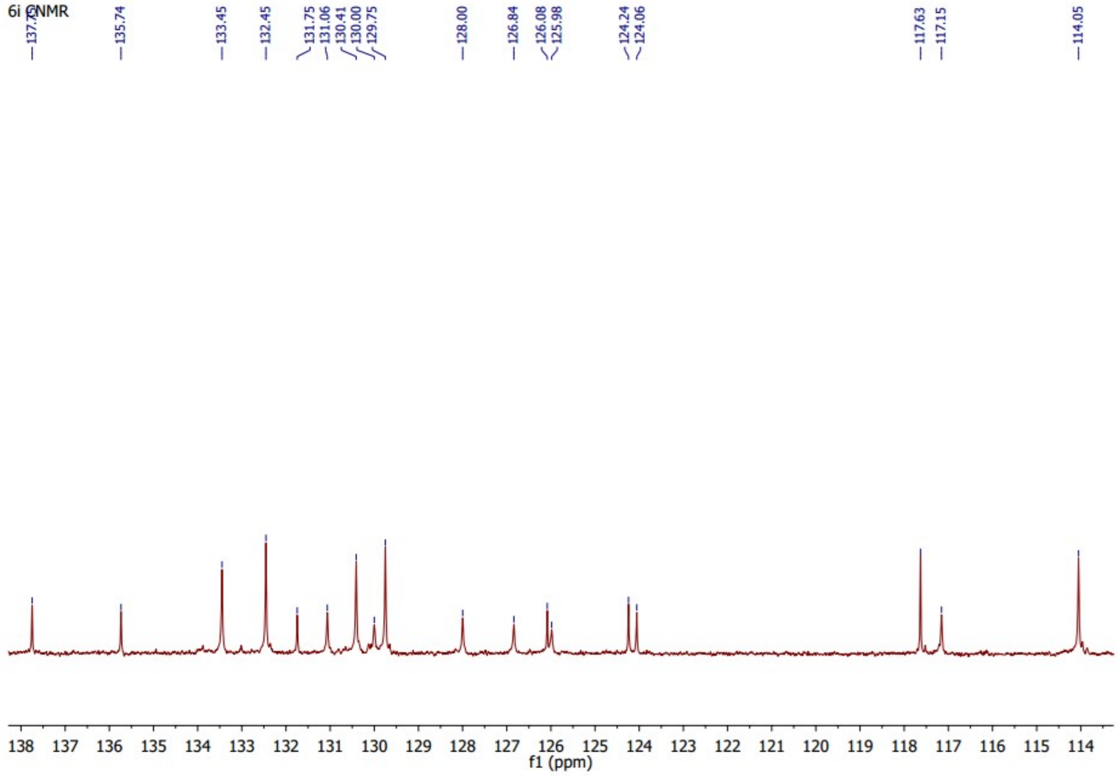


**Figure S57:** $^{13}$C-NMR spectrum of compound **6i (**zoom-in window**)**

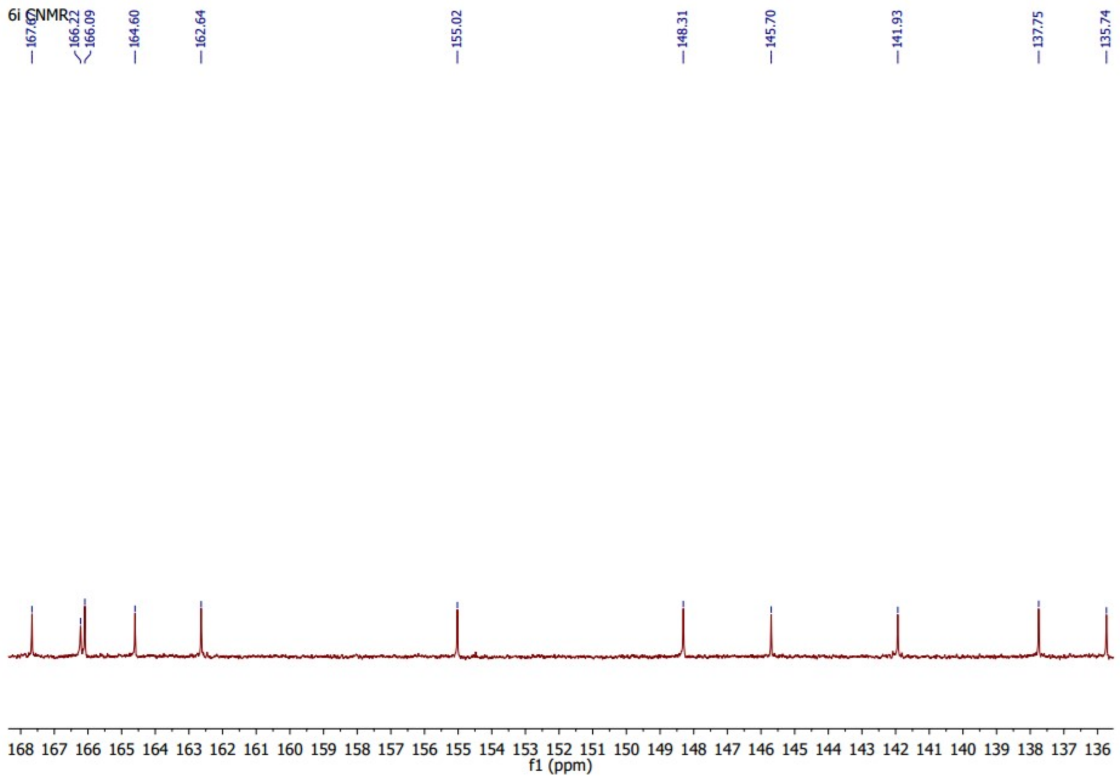


**Figure S58:** $^{13}$C-NMR spectrum of compound **6i (**zoom-in window**)**

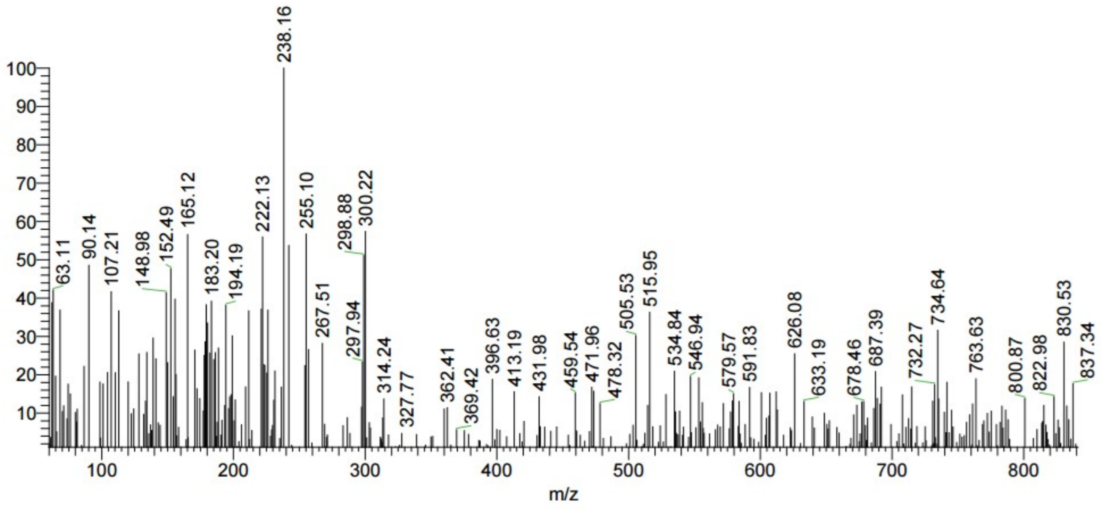


**Figure S59:** Mass spectrum of compound **6i**

## Appendix A

### S4.2. Biological Studies

#### S4.2.1. Cytotoxic activity evaluation

To measure the cytotoxic actvity of the prepared derivatives **5** and **6a-i** in breast adenocarcinoma (MCF-7) cell line. Cell viability assay was assessed using MTT assay method. Cells at density of 1 x $10^4$ were seeded in a 96-well plate at 37 °C for 24 h under 5% $CO_2$. After incubation, the cells were treated with different concentrations of the test hybrid **5** and **6a-i** and incubated for 24 h, then 20 µl of MTT solution at 5 mg/mL was applied and incubated for 4 h at 37 °C. Dimethyl sulphoxide (DMSO) in volume of 100 µl was added to each well to dissolve the purple formazan that had formed. The color intensity of the formazan product, which represents the growth condition of the cells, is quantified by using an ELISA plate reader (EXL 800, USA) at 570 nm absorbance. The experimental conditions were carried out with at least three replicates, and the experiments were repeated at least three times.

#### S4.2.2. EGFR kinase Assay

Compounds **6a**, **6h** and Lapatinib were evaluated for their EGFR kinase inhibitory activity according to manufacturer's instructions using # BPS Bioscience *EGFR Kinase Assay Kit* Catalog # 40321.

BPS Bioscience

6042 Cornerstone Court W, Ste B
San Diego, CA 92121
**Tel:** 1.858.829.3082
**Fax:** 1.858.481.8694
**Email:** info@bpsbioscience.com

## Data Sheet

### *EGFR Kinase Assay Kit*

**Catalog # 40321**

**DESCRIPTION:** The epidermal growth factor receptor (EGFR; ErbB-1; HER1) is the cell-surface receptor for members of the epidermal growth factor family. Overexpression and/or hyperactivation of EGFR kinase is associated with several human cancers such as lung, glioblastoma, and epithelian tumors of the neck and head, leading to the development of anticancer therapeutics targeting EGFR. The *EGFR Kinase Assay Kit* is designed to measure EGFR Kinase activity for screening and profiling applications using Kinase-Glo® MAX as a detection reagent. The EGFR Kinase Assay Kit comes in a convenient 96-well format, with enough purified recombinant EGFR enzyme, EGFR substrate, ATP and kinase assay buffer for 100 enzyme reactions.

**COMPONENTS:**

| Catalog # | Reagent | Amount | Storage | |
|---|---|---|---|---|
| 40187 | EGFR (wild type) | 2 µg | -80°C | ***Avoid multiple freeze/ thaw cycles!*** |
| | 5x Kinase assay buffer | 1.5 ml | -20°C | |
| | ATP (500 µM) | 100 µl | -20°C | |
| 40217 | 50x PTK substrate Poly(Glu:Tyr 4:1) | 100 µl | -20°C | |
| | 96-well plate, white | 1 | Room Temp. | |

**MATERIALS OR INSTRUMENTS REQUIRED BUT NOT SUPPLIED:**
Kinase-Glo MAX (Promega #V6071)
Dithiothreitol (DTT, 1 M; optional)
Microplate reader capable of reading luminescence
Adjustable micropipettor and sterile tips
30°C incubator

**APPLICATIONS:** Useful for studying enzyme kinetics and screening small molecular inhibitors for drug discovery and HTS applications.

**STABILITY:** Up to 6 months when stored as recommended.

**REFERENCE:**

Nakamura, J.L. *Expert Opin. Ther. Targets* **11(4)**:463-472 (2007)

### S4.2.3. Cell cycle analysis of compound 6h

Cell cycle analysis in MCF-7 cells was investigated using fluorescent Annexin V-FITC/ PI detection kit (*BioVision* EZCell$^{TM}$ Cell Cycle Analysis Kit Catalog #K920) by flow cytometry assay. MCF-7 cells at a density of $2 \times 10^5$ per well were harvested and washed twice in PBS. After that, the cells were incubated at 37 °C and 5% $CO_2$. The medium was incubated with the tested compound **6h** at the $IC_{50}$ (µM) for 48 h, washed twice in PBS, fixed with 70% ethanol, rinsed again with PBS. Afterward, medium was stained with DNA fluorochrome PI for 15 min at 37 °C. The samples were immediately analyzed using *Facs Calibur* flow cytometer (Becton and Dickinson, Heidelberg, Germany).

### S4.2.4. Apoptosis assay for compound 6h

Apoptosis in MCF-7 cells was investigated using fluorescent Annexin V-FITC/ PI detection kit (*BioVision* Annexin V-FITC Apoptosis Detection Kit, Catalog #: K101) by flow cytometry assay. MCF-7 cells at a density of $2 \times 10^5$ per well were treated with compound **6h** at the $IC_{50}$ (μM) for 48 h, then the cells were harvested and stained with Annexin V-FITC/ PI dye for 15 min in the dark at 37 °C. The samples were immediately analyzed using *FACS Calibur* flow cytometer (Becton and Dickinson, Heidelberg, Germany).
